# Chromosomal-level assembly of *Juglans sigillata* genome using Nanopore, BioNano, and Hi-C analysis

**DOI:** 10.1093/gigascience/giaa006

**Published:** 2020-02-26

**Authors:** De-Lu Ning, Tao Wu, Liang-Jun Xiao, Ting Ma, Wen-Liang Fang, Run-Quan Dong, Fu-Liang Cao

**Affiliations:** 1 Central South University of Forestry and Technology, 498 Shaoshan South Rd, Changsha 410004, China; 2 Institute of Economic Forest, Yunnan Academy of Forestry and Grassland, 2 Lan'an Rd, Kunming 650201, China; 3 Yunnan Laboratory for Conservation of Rare, Endangered & Endemic Forest Plants, Public Key Laboratory of the State Forestry Administration; Yunnan Provincial Key Laboratory of Cultivation and Exploitation of Forest Plants, 2 Lan'an Rd, Kunming 650201, China; 4 Co-Innovation Center for the Sustainable Forestry in Southern China, Nanjing Forestry University, 159 Longpan Rd, Nanjing 210037, China

**Keywords:** *Juglans sigillata*, genome assembly, annotation, evolution

## Abstract

**Background:**

*Juglans sigillata*, or iron walnut, belonging to the order Juglandales, is an economically important tree species in Asia, especially in the Yunnan province of China. However, little research has been conducted on *J. sigillata* at the molecular level, which hinders understanding of its evolution, speciation, and synthesis of secondary metabolites, as well as its wide adaptability to its plateau environment. To address these issues, a high-quality reference genome of *J. sigillata* would be useful.

**Findings:**

To construct a high-quality reference genome for *J. sigillata*, we first generated 38.0 Gb short reads and 66.31 Gb long reads using Illumina and Nanopore sequencing platforms, respectively. The sequencing data were assembled into a 536.50-Mb genome assembly with a contig N50 length of 4.31 Mb. Additionally, we applied BioNano technology to identify contacts among contigs, which were then used to assemble contigs into scaffolds, resulting in a genome assembly with scaffold N50 length of 16.43 Mb and contig N50 length of 4.34 Mb. To obtain a chromosome-level genome assembly, we constructed 1 Hi-C library and sequenced 79.97 Gb raw reads using the Illumina HiSeq platform. We anchored ∼93% of the scaffold sequences into 16 chromosomes and evaluated the quality of our assembly using the high contact frequency heat map. Repetitive elements account for 50.06% of the genome, and 30,387 protein-coding genes were predicted from the genome, of which 99.8% have been functionally annotated. The genome-wide phylogenetic tree indicated an estimated divergence time between *J. sigillata* and *Juglans regia* of 49 million years ago on the basis of single-copy orthologous genes.

**Conclusions:**

We provide the first chromosome-level genome for *J. sigillata*. It will lay a valuable foundation for future research on the genetic improvement of *J. sigillata*.

## Data Description

### Background

Walnut is an important nut fruit with high nutritive value and is grown in temperate climates. The 2 most widely cultivated species of walnuts for commercial nut production in the world are the English or Persian walnut (*Juglans regia*) and the iron walnut (*Juglans sigillata*). *J. regia* is the globally cultivated well-known species, but *J. sigillata* (NCBI:txid224355) is still mostly unknown in Western scientific research despite being grown for its nuts in Yunnan province, China [1, 2], for many centuries. In southwest China, *J. sigillata* is an important edible nut crop and is also cultivated for its wood. The name refers to the many seal-like depressions (sigillatae) in the shell, and with its thick shell the species has been termed the “iron walnut” [2]. It is commonly distributed in the eastern Himalayas and western China, especially Yunnan, both in the wild and in cultivation. No less than 80 authorized or approved cultivars of *J. sigillata* have been produced after successful implementation of grafting technology, such as "Yangpao," "Santai," and "Xixiang" [3]. China is the largest producer of walnuts in the world, producing nearly half of the global walnut supply in 2017 [4]. Domestically, Yunnan is the nation's number 1 walnut producer, its acreage and yield making up >2,860,000 hectares and 945,330 tones, accounting for one-half and one-fourth, respectively, of China's crop in 2016 [5].

All species of the genus *Juglans* are diploid with 2n = 2x = 32 chromosomes [6]. *J. regia* is a sister member of *J. sigillata* in section Dioscaryon Dode. It is native to the mountainous regions of central Asia, but it has become the most widespread tree nut cultivated in the world [7]. Although walnut has been cultivated for centuries, walnut breeding has only started recently and only a few systematic molecular studies on walnut have been reported [8]. Because of its commercial value and acreage, far more gene sequences are available for *J. regia* than *J. sigillata* and other members of the same genus. A team from the University of California–Davis sequenced the Persian walnut variety "Chandler" in 2016 [9]. In this study the iron walnut variety "Yangpao" was used for the genome sequencing because it is one of the most popular varieties in Yunnan. Walnut genome sequence information obtained here might be beneficial for accelerating its rate of breeding and variety improvement.

### Sampling and sequencing

All samples at the vegetative growth stage were collected from a *J. sigillata* specimen collected in Guangming town, Yangbi Yi autonomous county, Yunnan province, China. For sequencing on the Oxford Nanopore GridION X5, genomic DNA was isolated and extracted from leaves of a single plant using the Plant Genomic DNA kit (Qiagen, Hilden, Germany) based on the manufacturer's instructions. The DNA sample was further purified using a Zymo Genomic DNA Clean and Concentrator-10 column (Zymo Research, Irvine, CA, USA). The purified DNA was then prepared for sequencing following the protocol provided with the genomic sequencing kit SQK-LSK108 (Oxford Nanopore Technologies [ONT], Oxford, UK). Single-molecule real-time sequencing of long reads was conducted on a GridION X5 platform (ONT, Oxford, UK) with 16 flow cells [10]. A total of 66.31 Gb of raw data (4.14 Gb per cell) with an average pass read length of 15.60 kb was generated after quality filtering, the longest of which was 283 kb (Supplementary Table S1). Compared with other sequencing platforms, Nanopore sequencing has more advantages in read length. In addition, a separate paired-end DNA library with an insert size of 400 bp was constructed and sequenced using the Illumina HiSeq X Ten platform to enable a genome survey and genome accuracy correction, and a total of 37.99 Gb of raw data were produced (Supplementary Table S2).

### Genome survey

The genome size of *J. sigillata* was estimated by the *k*-mer method [11] using sequencing data from the Illumina DNA library. Quality-filtered reads were subjected to 17-mer frequency distribution analysis using the Jellyfish program (Jellyfish, RRID:SCR_005491) [11]. The genome size (*G*) of *J. sigillata* was estimated using the following formula: *G* = (*N_k_*_-mer_−*N*_error__*_k_*_-mer_)/*D*, where *N_k_*_-mer_ is the number of *k*-mers, *N*_error__*_k_*_-mer_ is the number of *k*-mers with the depth of 1, and *D* is the *k*-mer depth. The count distribution of 17-mers followed a Poisson distribution, with the highest peak occurring at a depth of 51 (Supplementary Table S3 and Fig. S1). The estimated genome size was ∼618,792,510 bp. And the heterozygosity of the genome was evaluated using the *Arabidopsis thaliana* genome data fitting method [12, 13]. From this the heterozygosity rate of the *J. sigillata* genome was estimated to be ∼1.0% (Supplementary Fig. S2), which is a moderate level among the related species (Table 1 and Additional File 1).

**Table 1:** Genome summary of *J. sigillata* and closely related species

Parameter	*Carya illinoinensis*[18]	*Carya cathayensis*[18]	*Quercus lobata*[19]	*Betula pendula*[20]	*Juglans regia* [21]	* Juglans microcarpa* [21]	*Quercus robur* [22]	*Juglans sigillata*
Estimated genome size (Mb)	649.75	721.33	730	440	NR	NR	736	618.79
Heterozygosity rate	1.46	0.77	1.25	NR	NR	NR	1.52	1.0
Total assembly (Mb)	651.31	706.43	1,170	436	534.67	572.90	750	574.62
Contig N50 (kb)	77.23	101.58	24.31	49.45	15,066.22	11,553.27	69.35	4336.69
Scaffold N50 (Mb)	1.08	1.22	278.07	0.24	35.20	35.63	1.34	16.43
Contigs/scaffolds	61,935/43,503	53,100/40,425	NR/94,394	27,582/5,644	127/73	208/154	22,615/1,409	913/749
Proportion of gaps	NR	NR	NR	NR	NR	NR	2.94	5.65
Rate of the anchored assemblies (%)	NR	NR	NR	89	99	99	96	93
Protein-coding genes	31,075	32,907	61,773	28,153	31,425	29,496	25,808	30,387
Repeat sequence (%)	50.43	53.67	52	49.23	44.15	43.88	53.30	50.06
BUSCO (%)	90.5	91.3	88.9	89.5	96.0	95.2	89.2	93.1

NR: parameter not reported.

BUSCO v3 was used to assess genome assembly completeness. And datasets based on embryophyta_odb9 (1,440 single-copy orthologs).

### Genome assembly

ONT long reads were corrected with Canu v1.6 (Canu, RRID:SCR_015880) [14] (overlapper = mhap utgReAlign = true corMinCoverage = 5 minReadLength = 2000 minOverlapLength = 1000) and assembled with WTDBG v1.2.8 (WTDBG, RRID:SCR_017225) [15] (–tidy-reads 5000 -fo dbg -k 0 -p 21 -S 3 –rescue-low-cov-edges); the initial assembly was ∼531.62 Mb in length, with a contig N50 size of 4.25 Mb (Supplementary Table S4). Nanopolish 0.11.0 (Nanopolish, RRID:SCR_016157) used the quality-controlled Nanopore sequencing reads for improving the assembled genome [16]. After that, the assembly contigs were polished twice with Pilon 1.22 (Pilon, RRID:SCR_014731) using Illumina whole-genome shotgun data [17]. After 2 rounds of Pilon polishing, the corrected genome was ∼536.50 Mb in size, with a Contig N50 size of 4.31 Mb (Supplementary Table S5).

### Scaffolding with BioNano optical mapping

The purifed genomic DNA of *J. sigillata* was embedded in an agarose layer, digested with Nt.BspQI enzyme, and labeled. The molecules were counterstained using the protocol provided with the SaphyrPrep Reagent Kit (BioNano Genomics, San Diego, CA, USA). Samples were then loaded into SaphyrChips and imaged on a Saphyr imaging instrument (BioNano Genomics). After filtering using a molecule length cut-off of <150 kb, a molecule SNR of <2.75, a label SNR (signal-to-noise ratio) of <2.75, and a label intensity of >0.8, 149.64 Gb of BioNano clean data were obtained, with the N50 size of the labeled single molecules being 264.04 kb (Supplementary Table S6).

A molecular quality report was generated by aligning the BioNano library sequences to the Nanopore genome assembly, yielding a map rate of 80.7%. Using the Nanopore genome assembly data as a reference, a reference genome assembly was conducted on the basis of the clean BioNano data. A genome map consisting of 824 consensus maps was assembled, yielding a genome size of 570.94 Mb with an N50 size of 9.94 Mb. To obtain a longer scaffold, the *de novo* assembly of Nanopore reads was then mapped to the BioNano single-molecule genomic map using the Bionano Access 1.1.2 and Bionano Solve 3.2 hybrid-scaffolding pipeline with hybrid scaffolding parameters (non-haplotype without extend and split). After scaffolding, the contig assembly contained 899 scaffolds with a scaffold N50 of 9.94 Mb, gap number was 177, and the proportion of gaps accounted for 6.03% of the whole genome.

To fill the gaps in the scaffolds, the pipline [23] (-minMatch 8 -sdpTupleSize 8 -minPctIdentity 75 -bestn 1 -nCandidates 10 -maxScore -500 –noSplitSubreads) was used to map the Nanopore long reads to the genome assembly scaffolding with BioNano optical mapping. Reads from the Illumina DNA library (400 bp) were then aligned against the genome assembly using the BWA (BWA, RRID:SCR_010910) and the genome was polished using Pilon 1.22 once again with default parameters, yielding a final draft genome of ∼574.62 Mb, with only 164 gaps, gap length for 5.65% of the genome, and contig and scaffold N50 sizes of 4.34 and 16.43 Mb, respectively (Supplementary Table S7). Because of the advantages of Nanopore sequencing technology and BioNano sequencing technology, the assembly quality of the *J. sigillata* genome assembly is currently far superior to reference genomes of its close relatives (Table 1).

### Genome quality evaluation

To assess the completeness of the assembled *J. sigillata* genome, we performed BUSCO (BUSCO, RRID:SCR_015008) analysis [24] by searching against the embryophyta BUSCO (version 3.0). Among 1,440 total BUSCO groups searched, 1,341 and 19 BUSCO core genes were completed and partially identified, respectively, leading to a total of 93.1% BUSCO genes in the *J. sigillata* genome (Supplementary Table S8). In concert we checked whether the high duplication rate (10.5%) indicated allelic duplications in the assembled genome, using BWA to align and counting up the coverage statistics from the Illumina short reads [25]. The sequencing coverage of the duplicated genes is almost the same as that of single-copy genes (Supplementary Fig. S3), showing that these duplicated genes likely exist as independent and distinct copies in the genome.

### Chromosome assembly using Hi-C data

To further generate a chromosomal-level assembly of the genome, we took advantage of sequencing data from the Hi-C library [26, 27]. We performed quality control of Hi-C raw data using HiC-Pro v. 2.8.0 (HiC-Pro, RRID:SCR_017643) [28]. First, we used bowtie2 v. 2.2.5 (Bowtie, RRID:SCR_005476) [29] to compare the raw reads to the draft assembled sequence, and then low-quality reads were filtered out to build raw inter/intra-chromosomal contact maps. Our final valid data set was 21.31 Gb (37.13×), accounting for 28.46% of the total Hi-C sequencing data. We then used the LACHESIS pipeline (LACHESIS, RRID:SCR_017644) [30] to scaffold the *J. sigillata* genome to 16 pseudochromosomes with length ranging from 10.00 to 55.29 Mb. The total length of pseudochromosomes consisted of 93.0% of all genome sequences (Supplementary Fig. S4, Supplementary Table S9).

### Genome annotation

**Figure 1:**
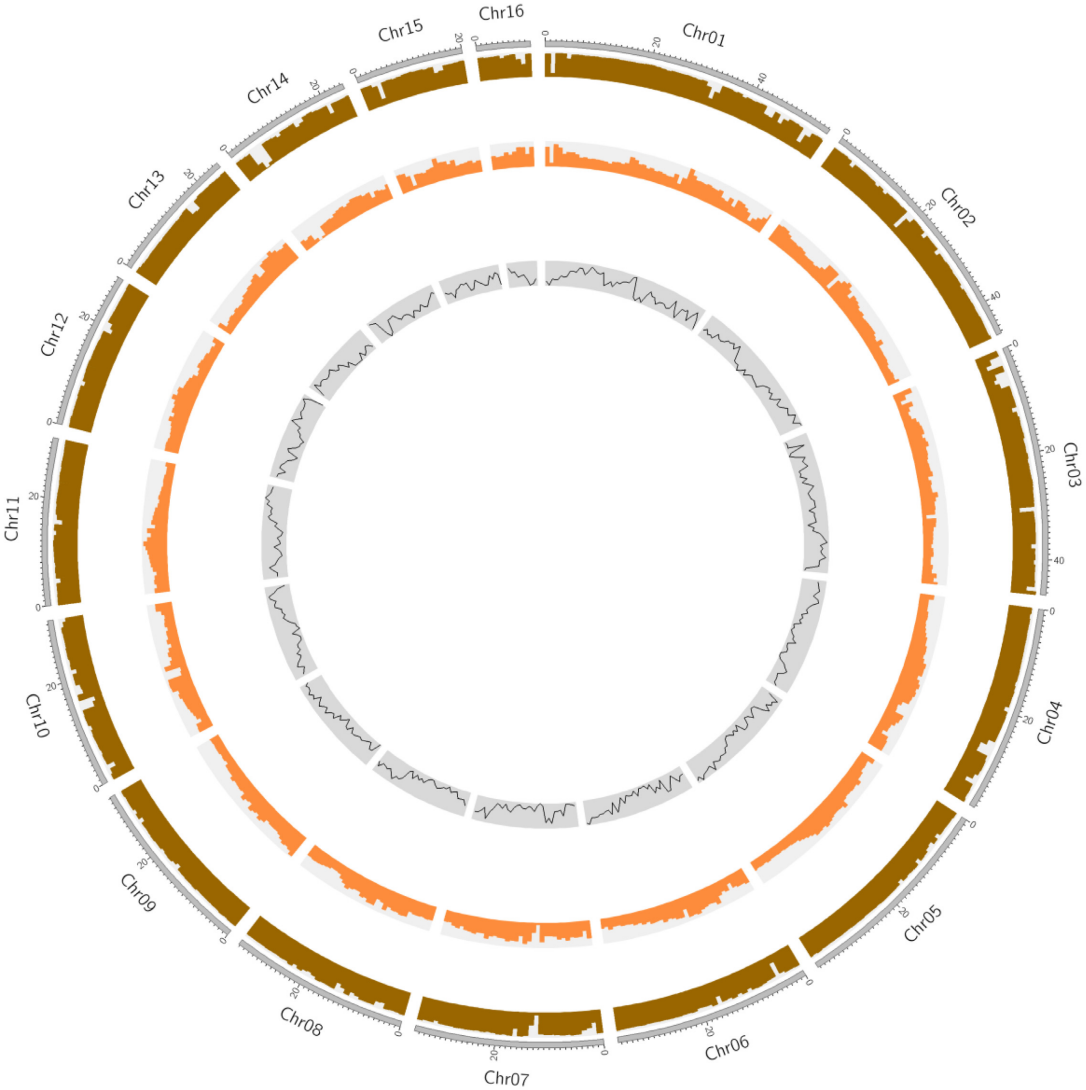
Circular diagram depicting the characteristics of the *J. sigillata* genome. The tracks from outer to inner circles indicate guanine-cytosine density, repeat density, and gene density.

To identify known transposable elements (TEs) in the *J. sigillata* genome, RepeatMasker (RepeatMasker, RRID:SCR_012954) [31] was used to screen the assembled genome against the Repbase (v. 22.11) [32] and Mips-REdat libraries [33]. In addition, *de novo* repeat annotation was performed using RepeatModeler v. 1.0.11 (RepeatModeler, RRID:SCR_015027) [28]. The combined results of the homology-based and *de novo* predictions indicated that repeated sequences account for 50.06% of the *J. sigillata* genome assembly, with long terminal repeats accounting for the greatest proportion (21.42%) (Supplementary Table S10 and Fig. 1).

Homology-based non-coding RNA annotation was performed by mapping plant ribosomal RNA (rRNA), microRNA, and small nuclear RNA genes from the Rfam database (release 13.0) [34] to the *J. sigillata* genome using BLASTN (BLASTN, RRID:SCR_001598) [35] (E-value ≤ 1e−5). tRNAscan-SE v1.3.1 (tRNAscan-SE, RRID:SCR_010835) [36] was used (with default parameters for eukaryotes) for transfer RNA (tRNA) annotation. RNAmmer v1.2 [37] was used to predict rRNAs and their subunits. These analyses identified 311 microRNAs, 807 tRNAs, 151 rRNAs, and 1,171 small nuclear RNAs (Supplementary Table S11).

To annotate genes in the *J. sigillata* genome, gene prediction was performed with homology-based, *de novo*, and transcriptome sequencing-based methods. For homology-based predictions, protein sequences from 5 species (*A. thaliana, Elaeis guineensis, Olea europaea, J. regia, Populus trichocarpa*) were mapped onto the *J. sigillata* genome using tBLASTn with an E-value of 1e−5; the aligned sequences and the corresponding query proteins were then filtered and passed to GeneWise v2.4.1 (GeneWise, RRID:SCR_015054) [38] to search for accurately spliced alignments. For the *de novo* predictions, we first randomly selected 1,000 full-length genes from the homology-based predictions to train model parameters for Augustus v3.0 (Augustus: Gene Prediction, RRID:SCR_008417) [39], Genemark [40], and GlimmerHMM (GlimmerHMM, RRID:SCR_002654) [41]. Augustus v3.0, Genemark, and GlimmerHMM were then used to predict genes based on the training set. We also used next-generation sequencing transcriptome short reads aligned on the *J. sigillata* genome using the TopHat (TopHat, RRID:SCR_013035) package [42]. Finally, EVidenceModeler v1.1.1 [43] was used to integrate the predicted genes and generate a consensus gene set. Genes with TEs were discarded using the TransposonPSI [44] package. Low-quality genes consisting of <50 amino acids and/or exhibiting premature termination (by aligning codons 1 by 1, the fragments with termination codons in the middle) were also removed from the gene set, yielding a final set of 30,387 genes. The final set's average transcript length, average CDS length, exon number per gene, average exon length, and average intron length were 4687.32 bp, 1257.18 bp, 5.49, 228.82 bp, and 763.25 bp, respectively (Supplementary Table S12 and Fig.   1).

The annotations of the predicted genes of *J. sigillata* were screened for homology against the Uniprot database (accessed 31 January 2018), KEGG database (accessed 87 July 2018), and InterPro database (5.21–60.0) using BLASTX (E value setting of 1e−5, coverage ≥50%, and identity ≥30% in BLAST v. 2.7.1+) [45], KAAS [46], and InterProScan package (release 5.2–45.0) [47]. In total, most (30,339) of the 30,387 genes were annotated by ≥1 database, representing 99.8% of the total genes (Supplementary Table S13).

### Phylogenetic tree construction and divergence time estimation

**Figure 2:**
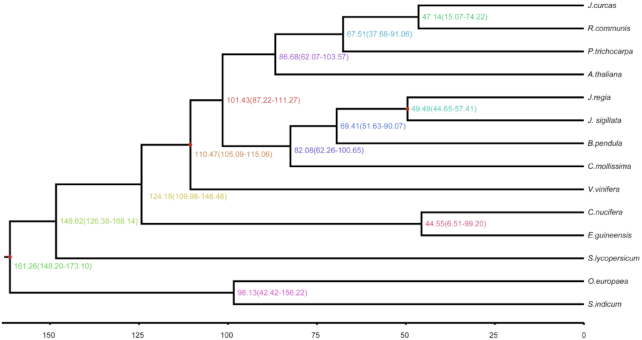
Inferred phylogenetic tree across 14 plant species. The estimated divergence time (Mya) is shown at each node. Numbers in parentheses indicate 95% confidence interval.

The detected *J. sigillata* genes were clustered in families using the OrthoMCL (v2.0.9) pipeline (OrthoMCL DB: Ortholog Groups of Protein Sequences, RRID:SCR_007839) [48], with an E-value cutoff of 1e−5, and Markov chain clustering with a default inflation parameter in an all-to-all BLASTP analysis of entries for 13 species (*A. thaliana, B. pendula, Castanea mollissima, Cocos nucifera, E. guineensis, Jatropha curcas, J. regia, O. europaea, P. trichocarpa, Ricinus communis, Sesamum indicum, Solanum lycopersicum, Vitis vinifera*). Gene family clustering identified 16,438 gene families containing 26,539 genes in *J. sigillata*. Of these, 141 gene families were unique to *J. sigillata* (Supplementary Table S14). Phylogenetic analysis was performed using 296 single-copy orthologous genes from common gene families found by OrthoMCL [43]. We codon-aligned each gene family using Mafft (MAFFT, RRID:SCR_011811) [49] and curated the alignments with Gblocks v0.91b (Gblocks, RRID:SCR_015945) [50]. Phylogeny analysis was performed using RAxML (RAxML, RRID:SCR_006086) v 8.2.11 [51] with the GTRGAMMA model and 100 bootstrap replicates. We then used MCMCTREE as implemented in PAML v4.9e (PAML, RRID:SCR_014932) [52] to estimate the divergence times of *J. sigillata* from the other plants. The parameter settings of MCMCTREE were as follows: clock = 2, RootAge ≤ 1.8, model = 7, BDparas = 110, kappa_gamma = 62, alpha_gamma = 11, rgene_gamma = 25.427, and sigma2_gamma = 11.03. In addition, the divergence times of *V. vinifera* (110–124 million years ago [Mya]) and *A. thaliana* (53–82 Mya) were used for fossil calibrations. The phylogenetic analysis showed that *J. sigillata, J. curcas*, and *B. pendula* diverged from a common ancestor ∼69.41 Mya. The estimated divergence time of *J. sigillata* and *J. regia* was 49.49 Mya (Fig. 2).

### Genes under positive selection

*J. sigillata* is an important cultivated tree that can be found growing on mountain slopes in southern China and in the Yunnan-Guizhou Plateau [53]. To evaluate adaptive evolution in the *J. sigillata* genome, we performed analysis to identify genes that are under positive selection. According to the neutral theory of molecular evolution [54], the ratio of nonsynonymous substitution rate (Ka) and synonymous substitution rate (Ks) of protein-coding genes can be used to identify genes that show signatures of natural selection. We calculated average Ka/Ks values and conducted the branch-site likelihood ratio test using Codeml implemented in the PAML package (PAML, RRID:SCR_014932) [52] to identify positively selected genes in the *J. sigillata* lineage. Twenty-five genes with signatures of positive selection were identified (*P* ≤ 0.05), of which 20 genes could be annotated with potential functions in the Swissprot database (Additional File 2). Gene ontology (GO) analysis using the DAVID program [55] (*P* ≤ 0.05) showed that 6 of these genes were related to chloroplast activity or function, and these 6 genes were ultraviolet-B receptor UVR8 (*UVR8*), carbamoyl-phosphate synthase large chain (*CARB*), PsbP domain-containing protein 6 (*PPD6*), probable N-acetyl-gamma-glutamyl-phosphate reductase (*At2g19940*), β-carotene isomerase D27(*D27*), and omega-amidase (*NLP3*). UVR8 is a photoreceptor for ultraviolet-B. Upon ultraviolet-B irradiation, UVR8 undergoes an immediate switch from homodimer to monomer, which triggers a signaling pathway for ultraviolet protection [56]. CARB is involved in arginine biosynthesis, and required for mesophyll development [57]. PPD6 is an important protein involved in the redox regulation of photosystem II [58]. D27 is an iron-binding protein that localizes in chloroplasts, required for the biosynthesis of strigolactones [59]. NLP3, involved in the metabolism of asparagine, probably also closely coupled with glutamine transamination in the methionine salvage cycle, can use α-ketosuccinamate and α-hydroxysuccinamate as substrates, producing, respectively, oxaloacetate and malate, or α-ketoglutaramate, producing α-ketoglutarate [60]. In conclusion, the functions of these genes ae closely related to systems including chloroplast defense mechanisms, photosynthesis, and amino acid metabolism, which might help *J. sigillata* adapt to the strong ultraviolet and high-altitude environment of the Yunnan plateau.

### Gene family expansion and contraction analysis

**Figure 3:**
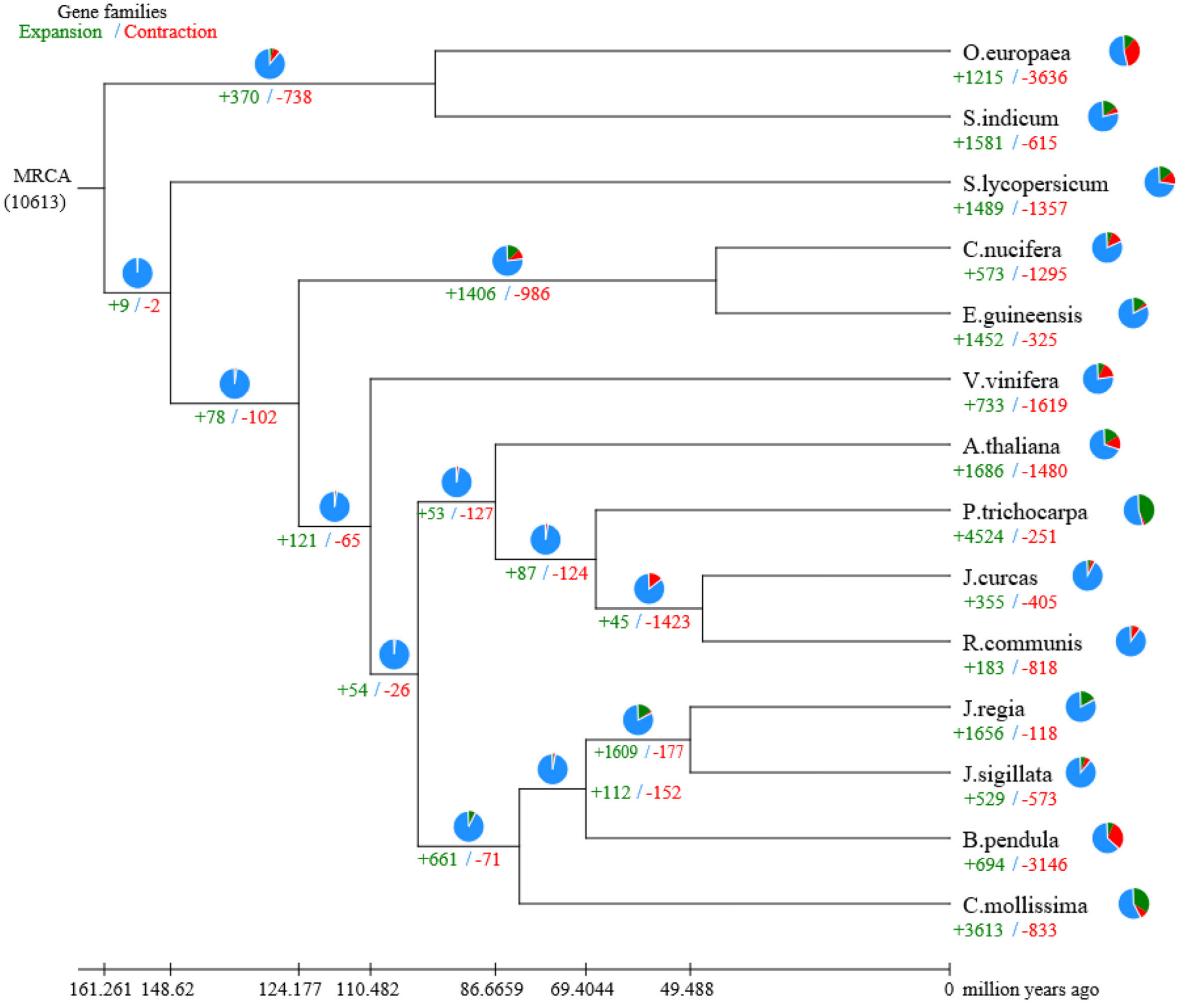
Gene family expansions and contractions in *J. sigillata* and 13 other plants. Pie charts show the proportion of expansion gene families (green), contraction gene families (red), and unaltered gene families (blue).

To understand the relationships of the *J. sigillata* gene families with those of other plants, we performed a systematic comparison of genes among different species. The protein-coding genes of 13 genomes, namely, *A. thaliana, B. pendula, C. nucifera, C. mollissima, E. guineensis, J. curcas, J. regia, O. europaea, P. trichocarpa, R. communis, S. indicum, S. lycopersicum*, and*V. vinifera*, were used for the comparison. Gene loss and gain are among the primary reasons for functional changes. To gain greater insights into the evolutionary dynamics of the genes, we determined the expansion and contraction of the orthologous gene clusters in these 14 species with CAFE software (CAFE, RRID:SCR_005983) [61]. This approach revealed 529 expanded gene families and 573 contracted gene families in the *J. sigillata* lineage (Fig. 3, Additional File 3). Furthermore, the enrichment pipeline software clusterProfiler [62] (clusterProfiler, RRID:SCR_016884) was used to test the statistical enrichment of expanded and contracted gene families in KEGG and GO pathway analysis. Pathways with Q-value < 0.05 (Q-values are the name given to the adjusted *P*-values found using an optimized false discovery rate approach [63]) were considered to be significantly enriched. There were no statistically significant enrichments in KEGG and GO analysis of the contracted gene families (Q-value > 0.05). The expanded gene families were enriched for 87 significant (Q-value < 0.05) GO terms at level 4 (Additional File 3). The significantly enriched KEGG pathways included “plant-pathogen interactions” (65 [12.29%]), “mRNA surveillance pathway” (44 [8.31%]), “Phospholipase D signaling pathway” (31 [5.86%]), “Fc gamma R-mediated phagocytosis” (31 [5.86%]), and “cAMP signaling pathway” (31 [5.86%]) (Additional File 3 and Supplementary Fig. S5).

## Conclusion

This article reports a chromosome-level reference genome sequence of *J. sigillata* using multiple types of sequencing data and assembly technologies. The assembled highly accurate genome will provide a valuable resource for studying the species’ evolutionary history, genetic changes, and associated biological phenomena, such as genetic load and selection pressures that occurred during severe bottlenecks or other unknown historical events. The *J. sigillata* genome lays a solid foundation for additional genomic studies in nut crops and related species, as well providing valuable resources for plant breeders.

## Availability of Supporting Data and Materials

The raw sequence data and *J. sigillata* genome data have been deposited in the Short Read Archive under NCBI BioProject ID PRJNA509030. The genome assembly, annotations, and other supporting data are available via the *GigaScience* database GigaDB [64].

## Additional Files

Additional File 1: The genome survey in the related species.

Additional File 2: The genes under positive selection.

Additional File 3: The KEGG and GO pathway analysis of expanded and contracted gene families.

Supplementary Table S1: Summary of Nanopore sequencing.

Supplementary Table S2: Summary of Illumina sequencing.

Supplementary Table S3: Estimation of genome size based on 17-mer statistics.

Supplementary Table S4: Statistics of initial assembly results.

Supplementary Table S5: Summary of the polished genome assembly.

Supplementary Table S6: Summary of the BioNano optical mapping data.

Supplementary Table S7: Summary of the final genome assembly.

Supplementary Table S8: Summary of BUSCO analysis results.

Supplementary Table S9: Statistics of pseudochromosomes of the *J. sigillata*.

Supplementary Table S10: Repeat annotation of the *J. sigillata* genome assembly.

Supplementary Table S11: Summary of non-protein-coding gene annotations in the *J. sigillata* genome assembly.

Supplementary Table S12: The comparative gene statistics of *J. sigillata* and 5 related species.

Supplementary Table S13: Functional annotation of predicted genes of *J. sigillata*.

Supplementary Table S14: Summary statistics of gene families in 14 plant species.

Supplementary Figure S1: Frequency distribution of the 17-mer graph analysis used to estimate the size of the *J. sigillata* genome.

Supplementary Figure S2: Schematic diagram of simulation curve of *J .sigillata* heterozygosity rate.

Supplementary Figure S3: Trendgram of mean coverage (Illumina short reads) of single-copy genes and duplicated genes.

Supplementary Figure S4: Interaction freqency distribution of Hi-C links among chromosomes.

Supplementary Figure S5: Significantly enriched KEGG pathways of genes in expanded families.

giaa006_GIGA-D-18-00511_Original_Submission

giaa006_GIGA-D-18-00511_Revision_1

giaa006_GIGA-D-18-00511_Revision_2

giaa006_GIGA-D-18-00511_Revision_3

giaa006_Response_to_Reviewer_Comments_Original_Submission

giaa006_Response_to_Reviewer_Comments_Revision_1

giaa006_Response_to_Reviewer_Comments_Revision_2

giaa006_Reviewer_1_Report_Original_Submission Jean-Marc Aury -- 1/24/2019 Reviewed

giaa006_Reviewer_1_Report_Revision_1 Jean-Marc Aury -- 6/7/2019 Reviewed

giaa006_Reviewer_1_Report_Revision_2 Jean-Marc Aury -- 10/21/2019 Reviewed

giaa006_Reviewer_2_Report_Original_Submission Jarkko Salojarvi, DSc (tech) -- 2/27/2019 Reviewed

giaa006_Reviewer_2_Report_Revision_1 Jarkko Salojarvi, DSc (tech) -- 6/28/2019 Reviewed

giaa006_Reviewer_2_Report_Revision_2 Jarkko Salojarvi, DSc (tech) -- 11/3/2019 Reviewed

giaa006_Supplemental_Files

## Abbreviations

BLAST: Basic Local Alignment Search Tool; bp: base pair; BUSCO: Benchmarking Universal Single-Copy Orthologs; BWA: Burrows-Wheeler Aligner; Gb: gigabase pairs; GO: gene ontology; KEGG: Kyoto Encyclopedia of Genes and Genomes; Hi-C: high-throughput chromosome conformation capture; KAAS: KEGG Automatic Annotation Server; kb: kilobase pairs; Mb: megabase pairs; Mya: million years ago; NCBI: National Center for Biotechnology Information; ONT: Oxford Nanopore Technologies; PAML: Phylogenetic Analysis by Maximum Likelihood; RAxML: Randomized Axelerated Maximum Likelihood; rRNA: ribosomal RNA; TE: transposable element; tRNA: transfer RNA.

## Competing Interests

The authors declare that they have no competing interests.

## Funding

This work was financially supported by the Yunnan Provincial Science and Technology Major Project (2018ZG001 and 2018ZG002) and the Science and Technology Innovation Program of Forestry Department of Yunnan Province ([2014]cx01 and [2016]cx03).

## Authors' Contributions

F.C., D.N., and T.W. designed the study and contributed to the project coordination.; L.X., T.W., T.M., W.F., and R.D. collected the sample and extracted the genomic DNA. T.W., L.X., and T.M. performed research and/or analyzed data. T.W. wrote the manuscript. All authors reviewed the manuscript.
