# Supplementary material for: Chromosomal-level assembly of Juglans sigillata genome using Nanopore, BioNano, and Hi-C analysis
Source: Gigascience. 2020 Feb 26;9(2):giaa006. doi: 10.1093/gigascience/giaa006 (PMC7043058; doi:10.1093/gigascience/giaa006)
Supplement: giaa006_GIGA-D-18-00511_Original_Submission [file giaa006_giga-d-18-00511_original_submission.pdf]

GigaScience

Chromosomal-level assembly of Juglans sigillata genome using Nanopore, BioNano and Hi-C analysis

--Manuscript Draft--

**Manuscript Number:**	GIGA-D-18-00511
**Full Title:**	Chromosomal-level assembly of Juglans sigillata genome using Nanopore, BioNano and Hi-C analysis
**Article Type:**	Data Note
**Funding Information:**	Yunnan Provincial Science and Technology Major Project (2018ZG001, 2018ZG002) — Mr. De-Lu Ning<br>the Science and Technology Innovation Program of Forestry Department of Yunnan Province ([2016]cx03) — Dr. Tao Wu<br>Forestry Department of Yunnan Province ([2014]cx01) — Mr. De-Lu Ning
**Abstract:**	Background<br><br>Juglans sigillata (NCBI: txid224355), belonging to Juglandales order, is an economically important tree species in Asia, especially in Yunnan province of China. However, little research has been conducted on J. sigillata at the molecular level, which hinders understanding of its evolution, speciation, and synthesis of secondary metabolites, as well as its wide adaptability to the plateau environment. To address these issues, a high-quality reference genome of J. sigillata would be a very useful resource.<br><br>Findings<br><br>To construct a high-quality reference genome for J. sigillata, we first generated 38.0 Gb short reads and 66.31 Gb long reads using Illumina and Nanopore sequencing platforms, respectively. The sequencing data were assembled into a 536.50 Mb genome assembly with a contig N50 length of 4.31 Mb. Additionally, we applied BioNano technology to identify contacts among contigs, which were then used to assemble contigs into scaffolds, resulting in a genome assembly with scaffold N50 length of 16.43 Mb and contig N50 length of 4.34 Mb. To obtain a chromosome-level genome assembly, we constructed one Hi-C library and sequenced 79.97 Gb raw reads using the Illumina HiSeq platform. We anchored approximately 93% of the scaffold sequences into 16 chromosomes and evaluated the quality of our assembly using the high contact frequency heatmap. Repetitive elements account for 50.06% of the genome, and 30,387 protein-coding genes were predicted from the genome, of which 99.8% have been functionally annotated. The genome-wide phylogenetic tree indicated the divergence time between J. sigillata and J.regia was estimated to be 49 million years ago (Mya) based on single-copy orthologous genes.<br><br>Conclusions<br><br>We provide the first chromosome-level genome for J. sigillata. The genome will lay a valuable foundation for future research on genetic improvement of
**Corresponding Author:**	Tao Wu, Ph.D.<br>Yunnan Academy of Forestry<br>Kunming, Yunnan CHINA
**Corresponding Author Secondary Information:**	
**Corresponding Author's Institution:**	Yunnan Academy of Forestry
**Corresponding Author's Secondary Institution:**	

Powered by Editorial Manager® and ProduXion Manager® from Aries Systems Corporation

**First Author:**	Tao Wu, Ph.D.
**First Author Secondary Information:**	
**Order of Authors:**	Tao Wu, Ph.D.
	Hai-Yun Chen
	Liang-Jun Xiao
	Ting Ma
	Wen-Liang Fang
	Run-Quan Dong
	De-Lu Ning
**Order of Authors Secondary Information:**	
**Additional Information:**	
**Question**	**Response**
Are you submitting this manuscript to a special series or article collection?	No
**Experimental design and statistics**<br><br>Full details of the experimental design and statistical methods used should be given in the Methods section, as detailed in our Minimum Standards Reporting Checklist. Information essential to interpreting the data presented should be made available in the figure legends.<br><br>Have you included all the information requested in your manuscript?	Yes
**Resources**<br><br>A description of all resources used, including antibodies, cell lines, animals and software tools, with enough information to allow them to be uniquely identified, should be included in the Methods section. Authors are strongly encouraged to cite Research Resource Identifiers (RRIDs) for antibodies, model organisms and tools, where possible.<br><br>Have you included the information requested as detailed in our Minimum Standards Reporting Checklist?	Yes

Powered by Editorial Manager® and ProduXion Manager® from Aries Systems Corporation

**Availability of data and materials**<br><br>All datasets and code on which the conclusions of the paper rely must be either included in your submission or deposited in publicly available repositories (where available and ethically appropriate), referencing such data using a unique identifier in the references and in the "Availability of Data and Materials" section of your manuscript.<br><br>Have you have met the above requirement as detailed in our Minimum Standards Reporting Checklist?	Yes

Click here to download Manuscript Chromosomal-level assembly of Juglans sigillata genome.docx

Chromosomal-level assembly of *Juglans sigillata* genome using Nanopore, BioNano and Hi-C analysis

Tao Wu[1,2], Hai-Yun Chen[1], Liang-Jun Xiao[1], Ting Ma[1], Wen-Liang Fang[1], Run-Quan Dong[1], De-Lu Ning[1]*

[1] Institute of Economic Forest, Yunnan Academy of Forestry, Kunming 650201, China

[2] Yunnan Laboratory for Conservation of Rare, Endangered & Endemic Forest Plants, Public Key Laboratory of the State Forestry Administration; Yunnan Provincial Key Laboratory of Cultivation and Exploitation of Forest Plants, Kunming 650201, China

* Corresponding author: ningdelu@163.com

Abstract

Background: *Juglans sigillata* (NCBI: txid224355), belonging to Juglandales order, is an economically important tree species in Asia, especially in Yunnan province of China. However, little research has been conducted on *J. sigillata* at the molecular level, which hinders understanding of its evolution, speciation, and synthesis of secondary metabolites, as well as its wide adaptability to the plateau environment. To address these issues, a high-quality reference genome of *J. sigillata* would be a very useful resource.

Findings: To construct a high-quality reference genome for *J. sigillata*, we first generated 38.0 Gb short reads and 66.31 Gb long reads using Illumina and Nanopore sequencing platforms, respectively. The sequencing data were assembled into a 536.50 Mb genome assembly with a contig N50 length of 4.31 Mb. Additionally, we applied BioNano technology to identify contacts among contigs, which were then used to

assemble contigs into scaffolds, resulting in a genome assembly with scaffold N50 length of 16.43 Mb and contig N50 length of 4.34 Mb. To obtain a chromosome-level genome assembly, we constructed one Hi-C library and sequenced 79.97 Gb raw reads using the Illumina HiSeq platform. We anchored approximately 93% of the scaffold sequences into 16 chromosomes and evaluated the quality of our assembly using the high contact frequency heatmap. Repetitive elements account for 50.06% of the genome, and 30,387 protein-coding genes were predicted from the genome, of which 99.8% have been functionally annotated. The genome-wide phylogenetic tree indicated the divergence time between *J. sigillata* and *J.regia* was estimated to be 49 million years ago (Mya) based on single-copy orthologous genes.

Conclusions: We provide the first chromosome-level genome for *J. sigillata*. The genome will lay a valuable foundation for future research on genetic improvement of *J. sigillata*.

Keywords: *Juglans sigillata*; genome assembly; annotation; evolution

Data Description

Background

Walnut is an important nut fruit with high nutritive value, grown in temperate climate. The two most widely cultivated species of walnuts for commercial nut production in the world are English or Persian walnut (*Juglans regia*) and iron walnut (*J. sigillata*). The former, *J. regia* is the globally cultivated well-known species, but the latter, *J. sigillata* is apparently still unknown in western scientific research despite being grown

for its nuts over the centuries in Yunnan province, China[1,2]. *J. sigillata* is an important edible nut crop. The name refers to the many seal-like depressions (sigillatae) in the shell, and the species has received recognition in China as the "iron walnut"[2]. It is commonly distributed in eastern Himalaya and western China, especially Yunnan, both in the wild and in cultivation. No less than 80 authorized or approved cultivars of *J. sigillata* have been popularized after successful implementation of grafting technology, such as ‘Yangpao’, ‘Santai’, ‘Xixiang’[3]. China is the largest producer of walnuts in the world, producing nearly half of the global walnut supply (FAOSTAT; http://www.fao.org/faostat/en/#data/QC)). Domestically, Yunnan is the nation’s number one walnut producer, its acreage and yield occurring on over 2860000 ha and 945330 t, accounting for one half and one-fourth of whole China[4].

All species of the genus *Juglans* are diploid with 2n = 2x = 32 chromosomes[5]. *J. regia* is sister member of *J. sigillata* in section Dioscaryon Dode, it is native to the mountainous regions of central Asia, but it has become the most widespread tree nut cultivated in the world[6]. Although walnut has been cultivated for centuries, walnut breeding starts recently and only a few systemic molecular studies on walnut have been reported [7]. Because of its commercial value and acreage, far more gene sequences are available for *J. regia* than *J. sigillata* and other members of the same genus. A team from the University of California-Davis sequenced the Persian walnut variety ‘Chandler’ in 2016[8]. Here, the walnut variety ‘Yangpao’ was used for the genome sequencing because it is one of the most famous variety in Yunnan. Walnut genome sequence information obtained here might be beneficial for accelerating its rate of

breeding and variety improvement.

Sampling and sequencing

All samples at the vegetative growth stage were collected from *J. sigillata* at Guangming town Yangbi Yi autonomous county Yunnan province, China. For sequencing on the GridION X5, gDNA was isolated and extracted from leaves of a single plant using the Plant Genomic DNA kit (Qiagen, Hilden) based on the manufacturer's instructions. DNA sample was further purified with the Zymo Genomic DNA Clean and Concentrator-10 column (Zymo Research, Irvine, CA). The purified DNA was then prepared for sequencing following the protocol in the genomic sequencing kit SQK-LSK108 (ONT, Oxford, UK). Single-molecule real-time sequencing of long reads was conducted on a GridION X5 platform (Oxford Nanopore Technology) with 16 Flow cells[9]. A total of 66.31 Gb of raw data（4.14 Gb per cell） with an average pass read length of 15.60 kb was generated after quality filtering, the longest of which is 283kb. (Supplementary Table S1). Compared with other sequencing platforms, Nanopore platform has more advantages in reading length. In addition, a separate paired-end (PE) DNA library with an insert size of 400 bp was constructed and sequenced using the Illumina platform to enable a genome survey and genome accuracy correction, and a total of 37.99 Gb of raw data was collected (Supplementary Table S2).

Genome survey

The genome size of *J. sigillata* was estimated by the K-mer method[10] using sequencing data from the Illumina DNA library. Quality-filtered reads were subjected to 17-mer

frequency distribution analysis using the Jellyfish program[10]. The genome size (G) of *J. sigillata* was estimated using the following formula: G = k-mer number/average k-mer depth, where k-mer number = total k-mers-abnormal k-mers. The count distribution of 17-mers followed a Poisson distribution, with the highest peak occurring at a depth of 51 (Supplementary Table S3 and Figure 1). The estimated genome size was approximately 618,792,510 bp, and the heterozygosity rate of the *J. sigillata* genome was approximately 1.0%.

Genome assembly

ONT long reads were corrected with Canu v1.6[11] and assembled with WTDBG v1.2.8[12], the initial assembly was approximately 531.62 Mb in length, with a Contig N50 size of 4.25 Mb (Supplementary Table S4). Nanopolish calibration uses the BWA default parameter to compare the quality-controlled Nanopore data to the assembled genome[13]. The second-generation data are then compared to the Nanopolish-corrected genome using the BWA default parameter, and the Pilon iteration is used to correct it two times[14], The corrected genome was approximately 536.50 Mb in size，with a Contig N50 size of 4.31 Mb (Supplementary Table S5).

Scaffolding with BioNano optical mapping

The purifed gDNA of *J. sigillata* was embedded in an agarose layer, digested with *Nt. BspQI* enzyme, and labeled. The molecules were counterstained using the protocol provided with the SaphyrPrep Reagent Kit (BioNano Genomics, San Diego, USA). Samples were then loaded into SaphyrChips and imaged on a Saphyr imaging

instrument (BioNano Genomics, San Diego, USA). After fltering using a molecule length cutoff of <150kb, a molecule SNR of <2.75, a label SNR of <2.75, and a label intensity of >0.8, 149.64 Gb of BioNano clean data were obtained, with the N50 size of the labeled single molecules being 264.04 kb (Supplementary Table S6).

A molecular quality report was generated by aligning the BioNano library sequences to the Nanopore genome assembly, yielding a map rate of 80.7%. Using the Nanopore genome assembly data as a reference, a reference genome assembly was conducted based on the clean BioNano data. A genome map consisting of 824 consensus maps was assembled, yielding a genome size of 570.94 Mb with an N50 size of 9.94 Mb. To obtain a longer scaffold, the *de novo* assembly of Nanopore reads was then mapped to the BioNano single-molecule genomic map. After scaffolding, the contig assembly contained 899 scaffolds with a scaffold N50 of 9.94 Mb.

To fill the gaps in the scaffolds, the Blasr pipline[15] was used to map the Nanopore long reads to the genome assembly scaffolding with BioNano optical mapping. The genome was polished using PBJelly 2 (RRID:SCR 012091) one time. Reads from the Illumina DNA library (400bp) were then aligned against the genome assembly using the BWA software (BWA, RRID:SCR 010910) to fill the gaps and correct potential sequencing errors of the assembly, yielding a final draft genome of approximately 574.62 Mb, with contig and scaffold N50 sizes of 4.34 Mb and 16.43 Mb, respectively (SupplementaryTable S7).

Genome quality evaluation

To assess the completeness of the assembled *J. sigillata* genome, we performed

Benchmarking Universal Single-Copy Orthologs (BUSCO) (RRID: SCR_015008) analysis[16] by searching against the embryophyta BUSCO (version 3.0). Among 1,440 total BUSCO groups searched, 1,341 and 19 BUSCO core genes were completed and partially identified, respectively, leading to a total of 93.1% BUSCO genes in *J. sigillata* genome (SupplementaryTable S8).

Chromosome assembly using Hi-C data

To further generate a chromosomal level assembly of the genome, we took advantage of sequencing data from the Hi-C library[17,18]. We performed quality control of Hi-C raw data using HiC-Pro (v. 2.8.0)[19]. First, we used bowtie2 (v. 2.2.5)[20] to compare the raw reads to the draft assembled sequence, and then low-quality reads were filtered out to build raw inter / intra-chromosomal contact maps. Our final valid data set was 21.31 Gb (37.13×), accounting for 28.46% of the total Hi-C sequencing data. We then used LACHESIS pipeline[21] to scaffold *J. sigillata* genome to 16 pseudochromosomes with length ranging from 10.00 Mb to 55.29 Mb. The total length of pseudochromosomes consisted of 93.0% of all genome sequences (Figure 2, SupplementaryTable S9).

Genome annotation

To identify known transposable elements (TEs) in the *J. sigillata* genome, RepeatMasker (RepeatMasker, RRID:SCR 012954)[22] was used to screen the assembled genome against the Repbase (v. 22.11)[23] and Mips-REdat libraries[24]. In addition, *de novo* evolved annotation was performed using RepeatModeler v. 1.0.11 (RepeatModeler, RRID:SCR 015027) [22]. The combined results of the homology-based

and *de novo* predictions indicated that repeated sequences account for 50.06% of the *J. sigillata* genome assembly, with long terminal repeats accounting for the greatest proportion (21.42%) (Supplementary Table S10).

Homology-based ncRNA annotation was performed by mapping plant rRNA, miRNA, and snRNA genes from the Rfam database (release 13.0) [25] to the *J. sigillata* genome using BLASTN[26] (E-value ≤ 1e−5). tRNAscan-SE v1.3.1 (tRNAscan-SE, RRID:SCR 010835) [27] was used (with default parameters for eukaryotes) for tRNA annotation. RNAmmer v1.2[28] was used to predict rRNAs and their subunits. These analyses identified 311 miRNAs, 807 tRNAs, 151 rRNAs, and 1,171 snRNAs (Supplementary Table S11).

To annotate genes in the *J. sigillata* genome, gene prediction was performed with homology-based, de novo, and transcriptome sequencing-based methods. For homology-based predictions, protein sequences from five species (*A. thaliana*, *E.guineensis*, *O.europaea*, *J.regia*, *P.trichocarpa*) were mapped onto the *J. sigillata* genome; the aligned sequences and the corresponding query proteins were then filtered and passed to GeneWise v2.4.1 (GeneWise, RRID:SCR 015054)[29]to search for accurately spliced alignments. For the *de novo* predictions, we first randomly selected 1,000 full-length genes from the homology-based predictions to trainmodel parameters for Augustus v3.0 (Augustus: Gene Prediction, RRID:SCR 008417)[30], Genemark[31], GlimmerHMM (GlimmerHMM, RRID:SC R 002654)[32]. Augustus v3.0[30], Genemark[31] and GlimmerHMM[32], were then used to predict genes based on the training set. We also used NGS transcriptome short reads aligned on *J. sigillata* genome

using the TopHat (TopHat, RRID:SCR_013035) package[33]. Finally, EVidenceModeler v1.1.1[34] was used to integrate the predicted genes and generate a consensus gene set. Genes with TEs were discarded using the TransposonPSI[35] package. Low quality genes consisting of fewer than 50 amino acids and/or exhibiting premature termination were also removed from the gene set, yielding a final set of 30,387genes. The final set's average transcript length, average CDS length, exon number per gene，average exon length and average intron length were 4,687.32 bp, 1,257.18 bp, 5.49, 228.82 bp，and 763.25 bp，respectively (Supplementary Table S12).

The annotations of the predicted genes of *J. sigillata* were screened for homology against the Uniprot and KEGG databases using Blastall[18] and KAAS[33]. Then, the InterProScan (release 5.2–45.0) [36] package was used to annotate the predicted genes using the InterPro (5.21–60.0) database. In total, most (30,339) of the 30,387 genes were annotated by at least one database, representing 99.8% of the total genes (Supplementary Table S13).

Phylogenetic tree construction and divergence time estimation

The detected *J. sigillata* genes were clustered in families using OrthoMCL (v2.0.9) pipeline (OrthoMCL DB: Ortholog Groups of Protein Sequences, RRID:SCR_007839) [37], with an E-value cutoff of 1e-5, and Markov Chain Clustering with a default inflation parameter in an all-to-all BLASTP analysis of entries for 13 species (*A.thaliana*，*B.pendula*，*C.mollissima*，*C.nucifera*，*E.guineensis*，*J.curcas*，*J.regia*，*O.europaea*，*P.trichocarpa*，*R.communis*，

S.indicum，*S.lycopersicum*，*V.vinifera*). The results indicated that Gene family clustering identified 16,438 gene families containing 26,539 genes in *J. sigillata*. Of these, 141 gene families were unique to *J. sigillata* (Supplementary Table S14). Phylogenetic analysis was performed using 296 single-copy orthologous genes from common gene families found by OrthoMCL[37]. We codon-aligned each gene family using Mafft[38] and curated the alignments with Gblocks v0.91b[39]. Phylogeny analysis was performed using RAxML (RAxML, RRID:SCR_006086) v 8.2.11[40] with the GTRGAMMA model and 100 bootstrap replicates. We then used MCMCTREE as implemented in PAML v4.9e (PAML, RRID:SCR_014932) [41] to estimate the divergence times of *J. sigillata* from the other plants. The parameter settings of MCMCTREE were as follows: clock = 2, RootAge ≤ 1.8, model = 7, BDparas = 110, kappa_gamma = 62, alpha_gamma = 11, rgene_gamma = 25.427, and sigma2_gamma = 11.03. In addition, the divergence times of *V. vinifera* (110–124 Mya) and *A. thaliana* (53–82 Mya) were used for fossil calibration. The phylogenetic analysis showed that *J. sigillata*, *J.curcas*, and *B.pendula* diverged from a common ancestor approximately 69.41 million years ago. And the estimated divergence time of *J. sigillata* and *J.curcas* was 49.49 Mya (Figure 3).

Genes under positive selection and gene family expansion analysis

According to the neutral theory of molecular evolution[42], the ratio of nonsynonymous substitution rate (Ka) and synonymous substitution rate (Ks) of protein coding genes can be used to identify genes that show signatures of natural selection. We calculated average Ka/Ks values and conducted the branch-site

likelihood ratio test using Codeml implemented in the PAML package[43] to identify positively selected genes in the *J. sigillata* lineage. These genes might contribute to the adaption to harsh environments. 25 genes with signatures of positive selection were identifed ($P \leq 0.05$), of which 20 genes could be annotated with potential functions in the Swissport database. Meanwhile, the OrthoMCL gene family analysis results were analyzed further by using CAFE[44] to detect expanded gene families. This approach revealed 529 expanded gene families and 573 contracted gene families in *J. sigillata* lineage (Figure 4). These genes may be related to the plateau adaptability and unique traits of *J. sigillata*, which may be helpful for further research.

Whole-genome duplication

We used 4DTv (four-fold synonymous third-codon transversion) [45] estimation and Ks (a measure of the substitutions per synonymous site) distribution[46] to detect WGD events in *J. sigillata* genome. To this end, paralogous sequences of *J. sigillata*, *S.lycopersicum* and *V. vinfira*, was identified with OrthoMCL[37]. Then, protein sequences for each of these plants were aligned against each other with Blastp (using an E-value threshold of ≤1e−5) to identify conserved paralogs in each species. Finally, potential WGD events in each genome were evaluated based on their 4DTv and Ks distribution. The WGD analysis suggested that *J. sigillata* experienced the same WGD events as other Dicotyledons, and that *J. sigillata* and *S.lycopersicum* also went through their recent WGD events (Figure 5).

Conclusion

This paper reports a chromosome-level reference genome sequence of *J. sigillata* using

multiple types of sequencing data and assembly technologies. The assembled precise genome will provide a valuable resource for studying the species' evolutionary history, genetic changes and associated phenomena, such as genetic load and selection pressures that occurred during its severe bottleneck or other unknown historical events. The *J. sigillata* genome laid a solid foundation for additional genomic studies in nut crop and related species.

Availability of supporting data

The raw sequence data have been deposited in the Short Read Archive under NCBI BioProject ID PRJNA509030.

Additional fles

Supplementary file.docx

Abbreviations

BUSCO: Benchmarking Universal Single-Copy Orthologs; Gb: giga base; kb: kilo base; Mb: mega base; bp: base pair; TE: transposable element; GO: gene ontology; Hi-C: high-throughput chromosome conformation capture; 4DTv: four-fold synonymous third-codon transversion.

Competing interests

The authors declare that they have no competing interests.

Funding

This work was financially supported by the Yunnan Provincial Science and Technology Major Project (2018ZG001 and 2018ZG002) and the Science and Technology Innovation Program of Forestry Department of Yunnan Province ([2016]cx03 and

[2014]cx01).

Author contributions

D. N. and T.W. conceived and designed the study; T. W., H. C., L. X., T. M., W. F. and R. D. collected the sample and extracted the genomic DNA. T. W., H. C., L. X., and T. M. performed research and/or analyzed data. T.W. wrote the manuscript. All authors reviewed the manuscript.

References

1. McGranahan G, Leslie C. Walnut. In: Badenes M, Byrne D. Fruit Breeding. Handbook of Plant Breeding, vol. 8. Springer, Boston, MA. 2012. p. 827-46.
2. Lu A, Stone DE, Grauke LJ. Juglandaceae. In: Wu ZY and Raven PH. Flora of China, vol. 4. Missouri Botanical Garden Press, St. Louis, Missouri. 1999. p. 277–85.
3. Zhang Y, Dong RQ, Xi XL. Germplasm Resource of Walnut in Yunnan and Its Exploitation and Utilization. Journal of Northwest Forestry University 2004;19(2):38-40.
4. Ministry of Forestry. China forestry statistical yearbook. Beijing: China Forestry Publishing House; 2017.
5. Woodworth RH. Meiosis of microsporogenesis in the Juglandaceae. Am J Bot 1930; 17(9):863-9.
6. Chen LN , Ma QG , Chen YK, et al. Identification of major walnut cultivars grown in China based on nut phenotypes and SSR markers. Scientia Horticulturae 2014, 168:240-8.
7. Britton MT, Leslie CA, Caboni E, et al. Persian Walnut. In: Chittaranjan K and Timothy CH. Compendium of transgenic crop plants: transgenic temperate fruits and nuts. Wiley-Blackwell, Massachusetts. 2008. p.189-232.
8. MartínezGarcía PJ, Crepeau MW, Puiu D, et al. The walnut (*Juglans regia*) genome sequence reveals diversity in genes coding for the biosynthesis of non-structural polyphenols. Plant Journal, 2016, 87(5):507-32.
9. Senol Cali D, Kim JS, Ghose S, et al. Nanopore sequencing technology and tools for genome assembly: computational analysis of the current state, bottlenecks and future directions. Briefings in bioinformatics. 2018; doi:10.1093/bib/bby017.
10. Marcais G and Kingsford CA. fast, lock-free approach for efficient parallel counting of occurrences of k-mers. Bioinformatics. 2011;27(6):764-70. doi:10.1093/bioinformatics/btr011.
11. Koren S, Walenz BP, Berlin K, et al. Canu: scalable and accurate long-read assembly via adaptive k-mer weighting and repeat separation. Genome research. 2017;27(5):722-36. doi:10.1101/gr.215087.116.
12. WTDBG package：https://github.com/ruanjue/wtdbg. (Accessed 10 Jan 2018).
13. Loman NJ, Quick J and Simpson JT. A complete bacterial genome assembled de novo using only nanopore sequencing data. Nature methods. 2015;12:733. doi:10.1038/nmeth.3444
14. Walker BJ, Abeel T, Shea T, et al. Pilon: an integrated tool for comprehensive microbial variant detection and genome assembly improvement. PloS one. 2014;9(11):e112963. doi:10.1371/journal.pone.0112963.
15. Chaisson MJ and Tesler G. Mapping single molecule sequencing reads using basic local alignment with successive refinement (BLASR): application and theory. BMC bioinformatics. 2012;13:238. doi:10.1186/1471-2105-13-238.
16. Simão FA, Waterhouse RM, Ioannidis P, et al. BUSCO: assessing genome assembly and annotation completeness with single-copy orthologs. Bioinformatics. 2015;31(19):3210-2. doi:10.1093/bioinformatics/btv351.
17. Dudchenko O, Batra SS, Omer AD, et al. De novo assembly of the Aedes aegypti genome using Hi-C yields chromosome-length scaffolds. Science. 2017;356(6333):92.
18. Belton JM, McCord RP, Gibcus JH, et al. Hi-C: a comprehensive technique to capture the conformation of genomes. Methods. 2012;58(3):268-76. doi:10.1016/j.ymeth.2012.05.001.
19. Servant N, Varoquaux N, Lajoie BR, et al. HiC-Pro: an optimized and flexible pipeline for Hi-C data processing. Genome biology. 2015;16:259. doi:10.1186/s13059-015-0831-x.
20. Langmead B and Salzberg SL. Fast gapped-read alignment with Bowtie 2. Nature methods. 2012;9(4):357-9. doi:10.1038/nmeth.1923.
21. Korbel JO and Lee C. Genome assembly and haplotyping with Hi-C. Nature biotechnology.

2013;31(12):1099-101. doi:10.1038/nbt.2764.
22. Tarailograovac M and Chen N. Using RepeatMasker to identify repetitive elements in genomic sequences. John Wiley & Sons, Inc.; 2004.
23. Bao W, Kojima KK and Kohany O. Repbase Update, a database of repetitive elements in eukaryotic genomes. Mobile DNA 2015;6(1):11.
24. Nussbaumer T, Martis MM, Roessner SK, et al. MIPS PlantsDB: a database framework for comparative plant genome research. Nucleic Acids Research. 2013;41 Database issue:D1144-D51.
25. Kalvari I, Argasinska J, Quinones-Olvera N, et al. Rfam 13.0: shifting to a genome-centric resource for non-coding RNA families. Nucleic Acids Res. 2018;46 D1:D335-D42. doi:10.1093/nar/gkx1038.
26. Camacho C, Coulouris G, Avagyan V, et al. BLAST+: architecture and applications. BMC bioinformatics. 2009;10:421. doi:10.1186/1471-2105-10-421.
27. Lowe TM and Eddy SR. tRNAscan-SE: a program for improved detection of transfer RNA genes in genomic sequence. Nucleic acids research. 1997;25(5):955-64.
28. Lagesen K, Hallin P, Rodland EA, et al. RNAmmer: consistent and rapid annotation of ribosomal RNA genes. Nucleic acids research. 2007;35(9):3100-8. doi:10.1093/nar/gkm160.
29. Birney E and Durbin R. Using GeneWise in the Drosophila annotation experiment. Genome research. 2000;10(4):547-8.
30. Stanke M, Steinkamp R, Waack S et al. AUGUSTUS: a web server for gene finding in eukaryotes. Nucleic Acids Res. 2004;32 Web Server issue:W309-12. doi:10.1093/nar/gkh379.
31. Blanco E, Parra G and Guigó R. Using geneid to identify genes. Current Protocols in Bioinformatics. 2007;18(1):Unit 4.3.
32. Majoros WH, Pertea M and Salzberg SL. TigrScan and GlimmerHMM: two open source ab initio eukaryotic gene-finders. Bioinformatics. 2004;20(16):2878-9. doi:10.1093/bioinformatics/bth315.
33. Moriya Y, Itoh M, Okuda S, et al. KAAS: an automatic genome annotation and pathway reconstruction server. Nucleic acids research. 2007;35 Web Server issue:W182-5. doi:10.1093/nar/gkm321.
34. Haas BJ, Salzberg SL, Wei Z, et al. Automated eukaryotic gene structure annotation using EVidenceModeler and the Program to Assemble Spliced Alignments. Genome Biology. 2008;9(1):R7.
35. TransposonPSI: An Application of PSI-Blast to Mine (Retro-)Transposon ORF Homologies. http://transposonpsi.sourceforge.net/, Accessed 18 Mar 2018.
36. Quevillon E, Silventoinen V, Pillai S, et al. InterProScan: protein domains identifier. Nucleic Acids Res. 2005;33 Web Server issue:W116-20. doi:10.1093/nar/gki442.
37. Li L, Stoeckert Jr. CJ, Roos DS. OrthoMCL: identification of ortholog groups for eukaryotic genomes. Genome research. 2003;13(9):2178-89. doi:10.1101/gr.1224503.
38. Katoh K and Standley DM. MAFFT multiple sequence alignment software version 7: improvements in performance and usability. Molecular biology and evolution. 2013;30(4):772-80. doi:10.1093/molbev/mst010.
39. Talavera G and Castresana J. Improvement of phylogenies after removing divergent and ambiguously aligned blocks from protein sequence alignments. Systematic biology. 2007;56(4):564-77. doi:10.1080/10635150701472164.
40. Stamatakis A. RAxML version 8: a tool for phylogenetic analysis and post-analysis of large phylogenies. Bioinformatics. 2014;30(9):1312-3. doi:10.1093/bioinformatics/btu033.
41. Yang Z. Paml 4: phylogenetic analysis by maximum likelihood. Mol Biol Evol 2007;24(8):1586–91.
42. Gillespie JH. The status of the neutral theory: the neutral theory of molecular evolution. Science. 1984;224(4650):732-3. doi:10.1126/science.224.4650.732.
43. Yang Z. PAML 4: phylogenetic analysis by maximum likelihood. Molecular biology and

evolution. 2007;24(8):1586-91. doi:10.1093/molbev/msm088.
44. De Bie T, Cristianini N, Demuth JP et al. CAFE: a computational tool for the study of gene family evolution. Bioinformatics. 2006;22(10):1269-71. doi:10.1093/bioinformatics/btl097.
45. Kimura M. A simple method for estimating evolutionary rates of base substitutions through comparative studies of nucleotide sequences. Journal of molecular evolution. 1980;16 2:111-20.
46. Blanc G and Wolfe KH. Widespread paleopolyploidy in model plant species inferred from age distributions of duplicate genes. The Plant cell. 2004;16(7):1667-78. doi:10.1105/tpc.021345.

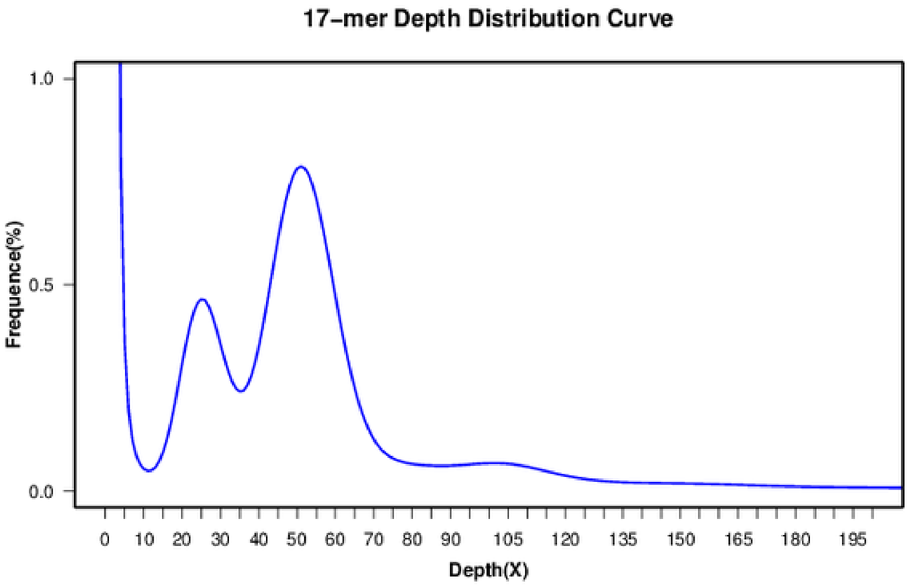

Figure 1. Frequency distribution of the 17-mer graph analysis uesd to estimate the size of the *J. sigillata* genome.

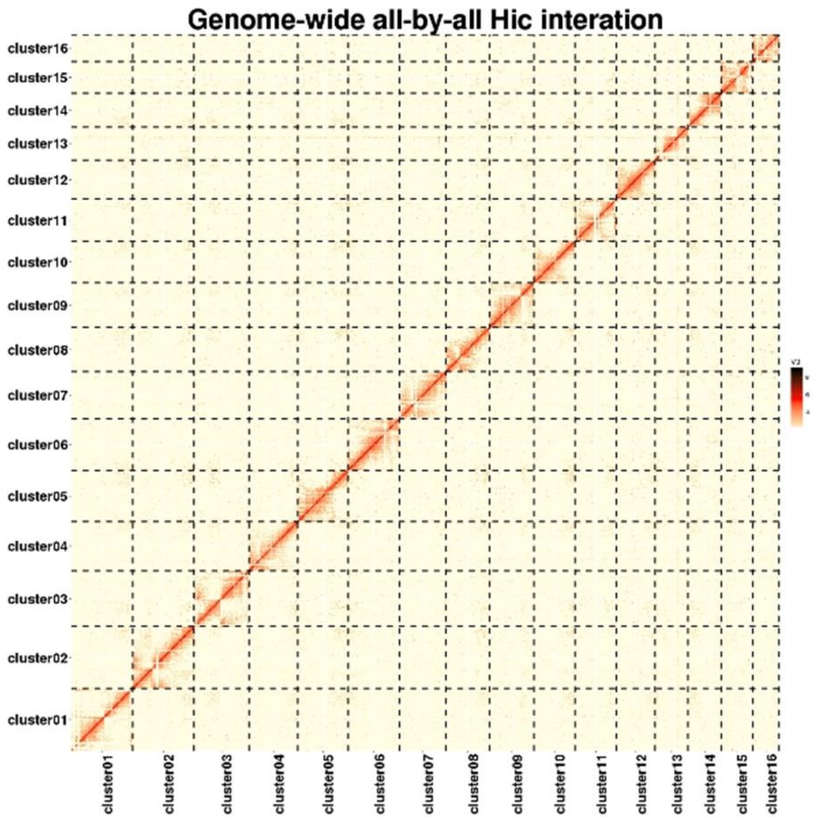

Figure 2. Interaction frequency distribution of Hi-C links among chromosomes.

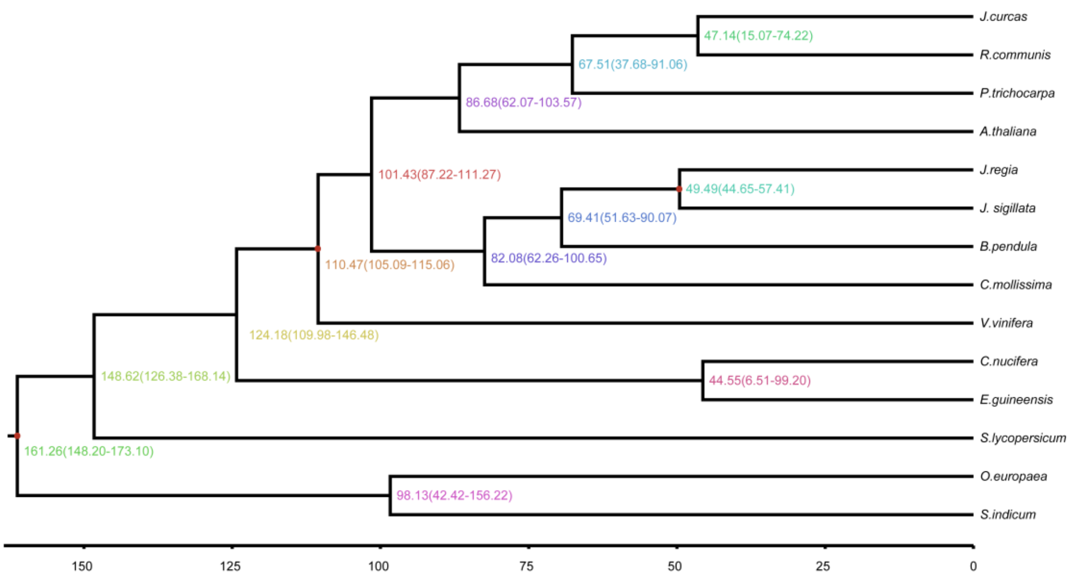

Figure 3. Inferred phylogenetic tree across 14 plant species. The estimated divergence time (Mya) is shown at each node.

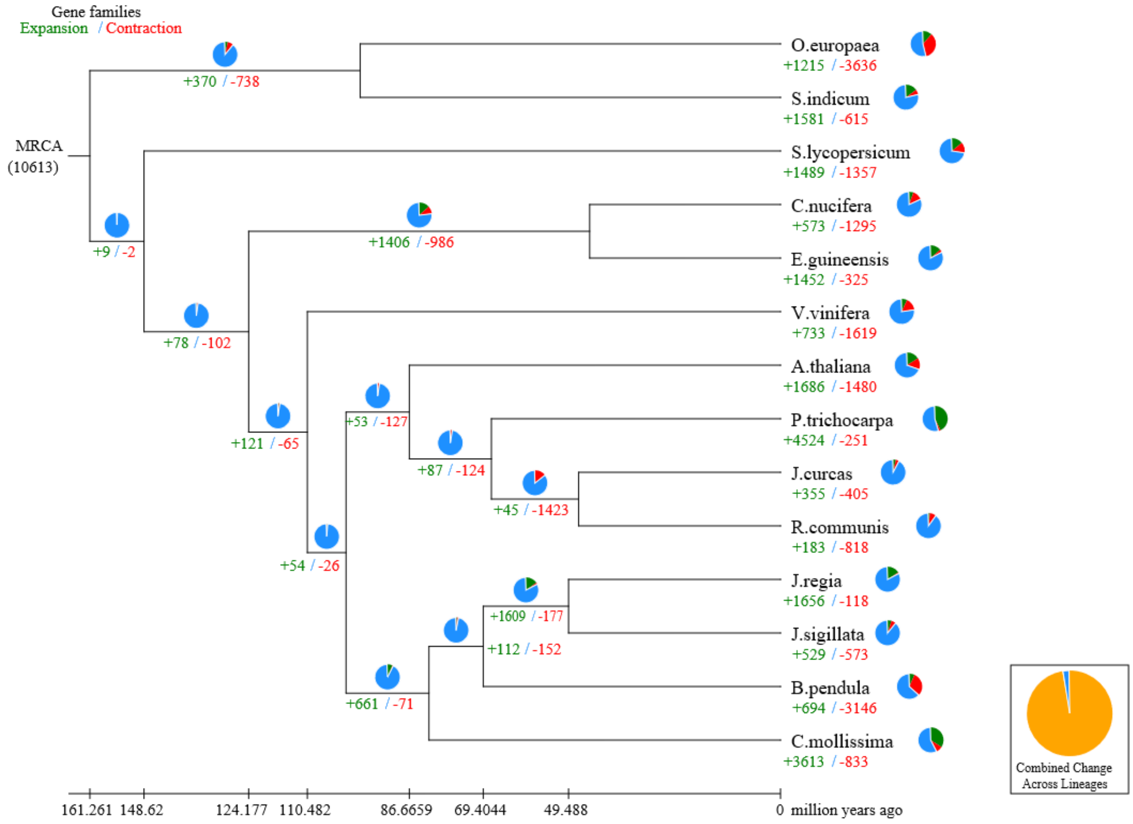

Figure 4. Gene family expansions and contractions in *J. sigillata* and 13 other plants.

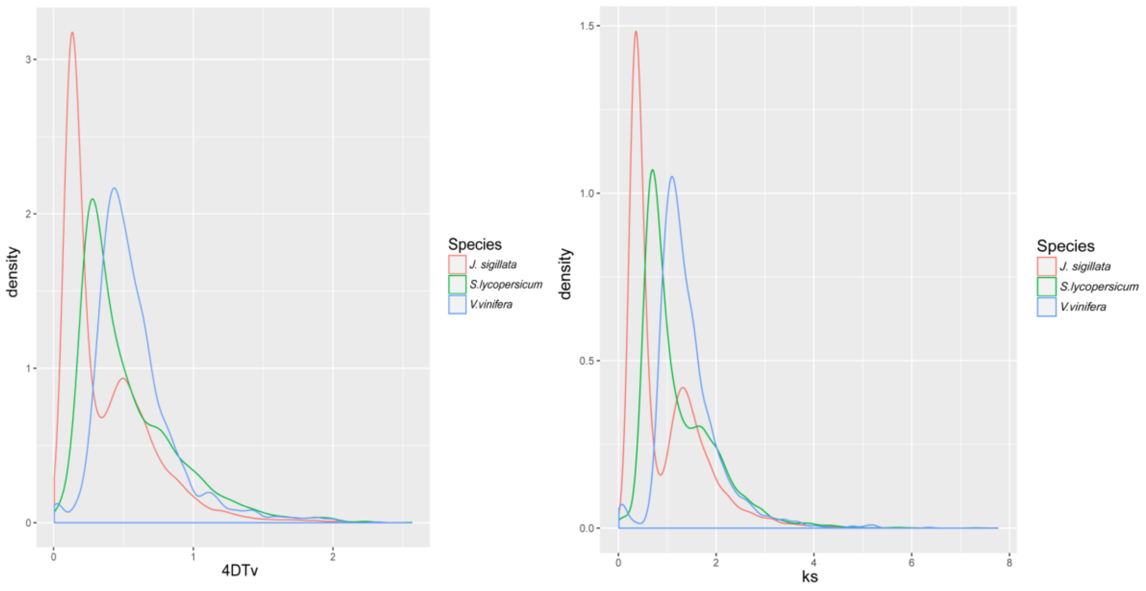

Figure 5. Whole-genome duplication (WGD) events of three plants (*J. sigillata, S. lycopersicum,* and *V. vinifera*) inferred by 4DTv and ks estimations.

Click here to access/download
Supplementary Material
Supplementary file.docx

Personal Cover Click here to download Personal Cover Personal Cover.docx

Chromosomal-level assembly of *Juglans sigillata* genome using Nanopore, BioNano and Hi-C analysis

Tao Wu[1,2], Hai-Yun Chen[1], Liang-Jun Xiao[1], Ting Ma[1], Wen-Liang Fang[1], Run-Quan Dong[1], De-Lu Ning[1]*

[1] Institute of Economic Forest, Yunnan Academy of Forestry, Kunming 650201, China

[2] Yunnan Laboratory for Conservation of Rare, Endangered & Endemic Forest Plants, Public Key Laboratory of the State Forestry Administration; Yunnan Provincial Key Laboratory of Cultivation and Exploitation of Forest Plants, Kunming 650201, China

*** Corresponding author**: ningdelu@163.com

Abstract

Background: *Juglans sigillata* (NCBI: txid224355), belonging to Juglandales order, is an economically important tree species in Asia, especially in Yunnan province of China. However, little research has been conducted on *J. sigillata* at the molecular level, which hinders understanding of its evolution, speciation, and synthesis of secondary metabolites, as well as its wide adaptability to the plateau environment. To address these issues, a high-quality reference genome of *J. sigillata* would be a very useful resource.

Findings: To construct a high-quality reference genome for *J. sigillata*, we first generated 38.0 Gb short reads and 66.31 Gb long reads using Illumina and Nanopore sequencing platforms, respectively. The sequencing data were assembled into a 536.50 Mb genome assembly with a contig N50 length of 4.31 Mb. Additionally, we applied BioNano technology to identify contacts among contigs, which were then used to

assemble contigs into scaffolds, resulting in a genome assembly with scaffold N50 length of 16.43 Mb and contig N50 length of 4.34 Mb. To obtain a chromosome-level genome assembly, we constructed one Hi-C library and sequenced 79.97 Gb raw reads using the Illumina HiSeq platform. We anchored approximately 93% of the scaffold sequences into 16 chromosomes and evaluated the quality of our assembly using the high contact frequency heatmap. Repetitive elements account for 50.06% of the genome, and 30,387 protein-coding genes were predicted from the genome, of which 99.8% have been functionally annotated. The genome-wide phylogenetic tree indicated the divergence time between *J. sigillata* and *J.regia* was estimated to be 49 million years ago (Mya) based on single-copy orthologous genes.

Conclusions: We provide the first chromosome-level genome for *J. sigillata*. The genome will lay a valuable foundation for future research on genetic improvement of *J. sigillata*.

Keywords: *Juglans sigillata*; genome assembly; annotation; evolution
