# Supplementary material for: Chromosomal-level assembly of Juglans sigillata genome using Nanopore, BioNano, and Hi-C analysis
Source: Gigascience. 2020 Feb 26;9(2):giaa006. doi: 10.1093/gigascience/giaa006 (PMC7043058; doi:10.1093/gigascience/giaa006)
Supplement: giaa006_GIGA-D-18-00511_Revision_3 [file giaa006_giga-d-18-00511_revision_3.pdf]

GigaScience

Chromosomal-level assembly of Juglans sigillata genome using Nanopore, BioNano and Hi-C analysis

--Manuscript Draft--

**Manuscript Number:**	GIGA-D-18-00511R3
**Full Title:**	Chromosomal-level assembly of Juglans sigillata genome using Nanopore, BioNano and Hi-C analysis
**Article Type:**	Data Note
**Funding Information:**	Yunnan Provincial Science and Technology Major Project (2018ZG001, 2018ZG002) — Mr. De-Lu Ning<br>the Science and Technology Innovation Program of Forestry Department of Yunnan Province ([2016]cx03) — Dr. Tao Wu<br>the Science and Technology Innovation Program of Forestry Department of Yunnan Province ([2014]cx01) — Mr. De-Lu Ning
**Abstract:**	Background<br>Juglans sigillata, belonging to Juglandales order, is an economically important tree species in Asia, especially in Yunnan province of China. However, little research has been conducted on J. sigillata at the molecular level, which hinders understanding of its evolution, speciation, and synthesis of secondary metabolites, as well as its wide adaptability to the plateau environment. To address these issues, a high-quality reference genome of J. sigillata would be a very useful resource. Findings To construct a high-quality reference genome for J. sigillata, we first generated 38.0 Gb short reads and 66.31 Gb long reads using Illumina and Nanopore sequencing platforms, respectively. The sequencing data were assembled into a 536.50 Mb genome assembly with a contig N50 length of 4.31 Mb. Additionally, we applied BioNano technology to identify contacts among contigs, which were then used to assemble contigs into scaffolds, resulting in a genome assembly with scaffold N50 length of 16.43 Mb and contig N50 length of 4.34 Mb. To obtain a chromosome-level genome assembly, we constructed one Hi-C library and sequenced 79.97 Gb raw reads using the Illumina HiSeq platform. We anchored approximately 93% of the scaffold sequences into 16 chromosomes and evaluated the quality of our assembly using the high contact frequency heatmap. Repetitive elements account for 50.06% of the genome, and 30,387 protein-coding genes were predicted from the genome, of which 99.8% have been functionally annotated. The genome-wide phylogenetic tree indicated the divergence time between J. sigillata and J.regia was estimated to be 49 million years ago (Mya) based on single-copy orthologous genes. Conclusions We provide the first chromosome-level genome for J. sigillata. The genome will lay a valuable foundation for future research on genetic improvement of J. sigillata.
**Corresponding Author:**	Tao Wu, Ph.D.<br>Yunnan Academy of Forestry<br>Kunming, Yunnan CHINA
**Corresponding Author Secondary Information:**	
**Corresponding Author's Institution:**	Yunnan Academy of Forestry
**Corresponding Author's Secondary Institution:**	
**First Author:**	De-Lu Ning
**First Author Secondary Information:**	
**Order of Authors:**	De-Lu Ning<br>Tao Wu, Ph.D.

Powered by Editorial Manager® and ProduXion Manager® from Aries Systems Corporation

	Liang-Jun Xiao
	Ting Ma
	Wen-Liang Fang
	Run-Quan Dong
	Fuliang Cao, Ph. D
**Order of Authors Secondary Information:**	
**Response to Reviewers:**	Reviewer reports:<br>Reviewer #1: The authors present an improved manuscript "Chromosome-level assembly of Juglans sigillata genome using Nanopore, BioNano and Hi-C analysis". However, there are still some issues to be resolved.<br>1) Please describe how the analysis on allelic genes was performed; citing the durian article is not enough. I could not find information in the method section of this article.<br>Response: Thank you! The analysis on allelic genes has been described in detail in lines 156-157. Durian article had also been replaced.<br><br>2) Please add in Table 1 the completion of the selected assemblies: BUSCO scores.<br>Response: Thank you very much for your advice. We have added BUSCO scores in Table 1.<br><br>3) Heterozygosity rates (HR) were not calculated using the same method (Table 1), and I verified that raw sequencing data were available for at least C. illinoinensis, C. cathayensis, Q. lobata, Q. robur, and B. pendula. Please calculate the HR using the same method to support line 102-103 of the article.<br>Response: Considering the reviewer’s suggestion, heterozygosity rates of C. illinoinensis, C. cathayensis, Q. lobata, and B. pendula have been calculated using the same method. The data volume of Q. robur is too little (only 6.1Gb) to calculate the heterozygosity. These results were shown in Additional file 1.<br><br>4) Some sentences must be verified: line 241, line 262-263, line 290-292.<br>Response: Considering the reviewer’s suggestion, we supplement the references in the corresponding text, and delete the farfetched speculative sentences: “This may help J. sigillata adapt to the plateau environment.”<br><br>5) Citation (1) from Table 1 is incorrect, please cite the GigaScience manuscript: https://academic.oup.com/gigascience/article/8/5/giz036/5484800<br>Response: Thank you! The citation has been revised.<br><br><br>Reviewer #2: The authors have addressed my comments from the previous rounds. There are still minor modifications:<br>1.1)l 65: walnut breeding starts recently and -> has started<br>1.2)l 87: reading length -> read length<br>1.3)l 11: quality-controlled of Nanopore sequencing reads (remove 'of')<br>1.4)l 136: Blasr pipline -> pipeline<br>1.5)l 142-143: gaps length accounting -> gap length<br>1.6)l 156-157: The data coverage depth trend of duplicated genes - maybe it would be better to say that "the sequencing coverage of the duplicated genes"<br>1.7)l 175: de novo evolved annotation -> de novo repeat annotation<br>1.8)l 223: The results indicated that Gene family clustering (Remove 'The results indicated that')<br>Response: Thank you for your correction. We have carefully corrected these inappropriate wordings.<br><br>2) l 235-236: Correct the English<br>Response: Thank you for your suggestion, the English has been corrected.<br><br>3) l 251-252: Gene ontology (GO) analysis (P ≤ 0.05) - define which method, additionally, did you use false discovery rate correction?<br>Response: Thanks! The method was added in the line 253, and we corrected it with Benjamini–Hochberg method.

	4) l 281-282: Further, enrichment pipeline software was used to test the statistical enrichment of expanded gene families in KEGG - What is "enrichment pipeline software" - please define.<br>Response: Thanks, we have revised the content to make it easier to understand (line 283-287).<br><br>5) l 281: Did you only use KEGG? The enriched categories sound like GOs.<br>Response: Thanks! We have added the analysis results of GOs, as shown in Additional file 3.<br><br>6) l 282 Q-values - a reference to the method. Give also a short statement what the Q values are.<br>Response: Thanks! Q-values are the name given to the adjusted p-values found using an optimized FDR approach (line 286-287).<br><br>7) The highlighted text l240-292 should still be corrected for English and made more fluent.<br>Response: Thank you for your suggestion. We have carefully revised the English description.
**Additional Information:**	
**Question**	**Response**
Are you submitting this manuscript to a special series or article collection?	No
**Experimental design and statistics**<br><br>Full details of the experimental design and statistical methods used should be given in the Methods section, as detailed in our Minimum Standards Reporting Checklist. Information essential to interpreting the data presented should be made available in the figure legends.<br><br>Have you included all the information requested in your manuscript?	Yes
**Resources**<br><br>A description of all resources used, including antibodies, cell lines, animals and software tools, with enough information to allow them to be uniquely identified, should be included in the Methods section. Authors are strongly encouraged to cite Research Resource Identifiers (RRIDs) for antibodies, model organisms and tools, where possible.<br><br>Have you included the information requested as detailed in our Minimum	Yes

Standards Reporting Checklist?	
**Availability of data and materials**<br><br>All datasets and code on which the conclusions of the paper rely must be either included in your submission or deposited in publicly available repositories (where available and ethically appropriate), referencing such data using a unique identifier in the references and in the "Availability of Data and Materials" section of your manuscript.<br><br>Have you have met the above requirement as detailed in our Minimum Standards Reporting Checklist?	Yes

Powered by Editorial Manager® and ProduXion Manager® from Aries Systems Corporation

Manuscript Click here to access/download;Manuscript;511-HL.docx

Chromosomal-level assembly of *Juglans sigillata* genome using Nanopore, BioNano and Hi-C analysis

De-Lu Ning[1,2,†] Tao Wu[2,3,†] Liang-Jun Xiao[2], Ting Ma[2], Wen-Liang Fang[2], Run-Quan Dong[2], Fu-Liang Cao[4*]

[1] Central South University of Forestry and Technology, Changsha 410004, China

[2] Institute of Economic Forest, Yunnan Academy of Forestry and Grassland, Kunming 650201, China

[3] Yunnan Laboratory for Conservation of Rare, Endangered & Endemic Forest Plants, Public Key Laboratory of the State Forestry Administration; Yunnan Provincial Key Laboratory of Cultivation and Exploitation of Forest Plants, Kunming 650201, China

[4] Co-Innovation Center for the Sustainable Forestry in Southern China, Nanjing Forestry University, Nanjing 210037, China

† These authors contributed equally.

* Corresponding author: CFL1957@qq.com

ORCIDs:

De-Lu Ning, 0000-0001-9152-0172

Tao Wu, 0000-0002-5371-9700

Liang-Jun Xiao, 0000-0003-4851-1574

Ting Ma, 0000-0002-9580-1729

Fu-Liang Cao, 0000-0002-0594-6968

Abstract

Background: *Juglans sigillata* or iron walnut, belonging to the Juglandales order, is an

economically important tree species in Asia, especially in Yunnan province of China. However, little research has been conducted on *J. sigillata* at the molecular level, which hinders understanding of its evolution, speciation, and synthesis of secondary metabolites, as well as its wide adaptability to its plateau environment. To address these issues, a high-quality reference genome of *J. sigillata* would be a very useful resource.

Findings: To construct a high-quality reference genome for *J. sigillata*, we first generated 38.0 Gb short reads and 66.31 Gb long reads using Illumina and Nanopore sequencing platforms, respectively. The sequencing data were assembled into a 536.50 Mb genome assembly with a contig N50 length of 4.31 Mb. Additionally, we applied BioNano technology to identify contacts among contigs, which were then used to assemble contigs into scaffolds, resulting in a genome assembly with scaffold N50 length of 16.43 Mb and contig N50 length of 4.34 Mb. To obtain a chromosome-level genome assembly, we constructed one Hi-C library and sequenced 79.97 Gb raw reads using the Illumina HiSeq platform. We anchored approximately 93% of the scaffold sequences into 16 chromosomes and evaluated the quality of our assembly using the high contact frequency heatmap. Repetitive elements account for 50.06% of the genome, and 30,387 protein-coding genes were predicted from the genome, of which 99.8% have been functionally annotated. The genome-wide phylogenetic tree indicated the divergence time between *J. sigillata* and *J.regia* was estimated to be 49 million years ago (Mya) based on single-copy orthologous genes.

Conclusions: We provide the first chromosome-level genome for *J. sigillata*. The genome will lay a valuable foundation for future research on the genetic improvement

of *J. sigillata*.

Keywords: *Juglans sigillata*; genome assembly; annotation; evolution

Data Description

Background

Walnut is an important nut fruit with high nutritive value, and is grown in temperate climates. The two most widely cultivated species of walnuts for commercial nut production in the world are the English or Persian walnut (*Juglans regia*) and the iron walnut (*J. sigillata*). The former, *J. regia* is the globally cultivated well-known species, but the latter, *J. sigillata* (NCBI: txid224355) is still mostly unknown in Western scientific research despite being grown for its nuts in Yunnan province, China[1,2] for many centuries. In Western China *J. sigillata* is an important edible nut crop and is also cultivated for its wood. The name refers to the many seal-like depressions (sigillatae) in the shell, and with its thick shell the species has been termed the "iron walnut"[2]. It is commonly distributed in eastern Himalayas and Western China, especially Yunnan, both in the wild and in cultivation. No less than 80 authorized or approved cultivars of *J. sigillata* have been produced after successful implementation of grafting technology, such as 'Yangpao', 'Santai', 'Xixiang'[3]. China is the largest producer of walnuts in the world, producing nearly half of the global walnut supply in 2017 (FAOSTAT; http://www.fao.org/faostat/en/#data/QC)). Domestically, Yunnan is the nation's number one walnut producer, its acreage and yield occurring making up over 2,860,000 hectares and 945,330 tones, accounting for one half and one-fourth respectively, of the whole of

China’s crop in 2016[4].

All species of the genus *Juglans* are diploid with 2n = 2x = 32 chromosomes[5]. *J. regia* is a sister member of *J. sigillata* in section Dioscaryon Dode. It is native to the mountainous regions of central Asia, but it has become the most widespread tree nut cultivated in the world[6]. Although walnut has been cultivated for centuries, walnut breeding has only started recently and only a few systemic molecular studies on walnut have been reported[7]. Because of its commercial value and acreage, far more gene sequences are available for *J. regia* than *J. sigillata* and other members of the same genus. A team from the University of California-Davis sequenced the Persian walnut variety ‘Chandler’ in 2016[8]. In this study the iron walnut variety ‘Yangpao’ was used for the genome sequencing because it is one of the most popular varieties in Yunnan. Walnut genome sequence information obtained here might be beneficial for accelerating its rate of breeding and variety improvement.

Sampling and sequencing

All samples at the vegetative growth stage were collected from a *J. sigillata* specimen collected in Guangming town, Yangbi Yi autonomous county, Yunnan province, China. For sequencing on the Oxford Nanopore GridION X5, gDNA was isolated and extracted from leaves of a single plant using the Plant Genomic DNA kit (Qiagen, Hilden) based on the manufacturer's instructions. The DNA sample was further purified using a Zymo Genomic DNA Clean and Concentrator-10 column (Zymo Research, Irvine, CA). The purified DNA was then prepared for sequencing following the protocol

provided with the genomic sequencing kit SQK-LSK108 (Oxford Nanopore Technologies, Oxford, UK). Single-molecule real-time sequencing of long reads was conducted on a GridION X5 platform (Oxford Nanopore Technologies, Oxford, UK) with 16 Flow cells[9]. A total of 66.31 Gb of raw data（4.14 Gb per cell） with an average pass read length of 15.60 kb was generated after quality filtering, the longest of which was 283kb. (Supplementary Table S1). Compared with other sequencing platforms, Nanopore sequencing has more advantages in read length. In addition, a separate paired-end (PE) DNA library with an insert size of 400 bp was constructed and sequenced using the Illumina HiSeq X Ten platform to enable a genome survey and genome accuracy correction, and a total of 37.99 Gb of raw data was produced for these efforts (Supplementary Table S2).

Genome survey

The genome size of *J. sigillata* was estimated by the K-mer method [10] using sequencing data from the Illumina DNA library. Quality-filtered reads were subjected to 17-mer frequency distribution analysis using the Jellyfish program (Jellyfish, RRID:SCR_005491) [10]. The genome size (G) of *J. sigillata* was estimated using the following formula: $G = (N_{k\text{-mer}} – N_{error\_k\text{-mer}}) / D$, where $N_{k\text{-mer}}$ is the number of *k*-mers, $N_{error\_k\text{-mer}}$ is the number of *k*-mers with the depth of 1, and D is the *k*-mer depth. The count distribution of 17-mers followed a Poisson distribution, with the highest peak occurring at a depth of 51 (Supplementary Table S3 and Figure S1). The estimated genome size was approximately 618,792,510 bp. And the heterozygosity of the genome was evaluated using the *Arabidopsis thaliana* genome data fitting method[11, 12]. From

this the heterozygosity rate of the *J.sigillata* genome was estimated to be approximately 1.0% (Supplementary Figure S2), which is a moderate level among the related species (Table 1 and Additional file 1).

Genome assembly

ONT long reads were corrected with Canu v1.6 (Canu, RRID:SCR_015880) [13] (overlapper=mhap utgReAlign=true corMinCoverage=5 minReadLength=2000 minOverlapLength=1000) and assembled with WTDBG v1.2.8 (WTDBG, RRID:SCR_017225) [14] （--tidy-reads 5000 -fo dbg -k 0 -p 21 -S 3 --rescue-low-cov-edges , the initial assembly was approximately 531.62 Mb in length, with a Contig N50 size of 4.25 Mb (Supplementary Table S4). Nanopolish 0.11.0 (Nanopolish, RRID:SCR 016157) used the quality-controlled Nanopore sequencing reads for improving the assembled genome[15]. After that, the assembly contigs were polished twice with Pilon 1.22 (Pilon, RRID:SCR 014731) using Illumina whole-genome shotgun data [16]. After two rounds of Pilon polishing, the corrected genome was approximately 536.50 Mb in size, with a Contig N50 size of 4.31 Mb (Supplementary Table S5).

Scaffolding with BioNano optical mapping

The purifed gDNA of *J. sigillata* was embedded in an agarose layer, digested with *Nt. BspQI* enzyme, and labeled. The molecules were counterstained using the protocol provided with the SaphyrPrep Reagent Kit (BioNano Genomics, San Diego, USA). Samples were then loaded into SaphyrChips and imaged on a Saphyr imaging instrument (BioNano Genomics, San Diego, USA). After filtering using a molecule

length cutoff of <150kb, a molecule SNR of <2.75, a label SNR of <2.75, and a label intensity of >0.8, 149.64 Gb of BioNano clean data were obtained, with the N50 size of the labeled single molecules being 264.04 kb (Supplementary Table S6).

A molecular quality report was generated by aligning the BioNano library sequences to the Nanopore genome assembly, yielding a map rate of 80.7%. Using the Nanopore genome assembly data as a reference, a reference genome assembly was conducted based on the clean BioNano data. A genome map consisting of 824 consensus maps was assembled, yielding a genome size of 570.94 Mb with an N50 size of 9.94 Mb. To obtain a longer scaffold, the *de novo* assembly of Nanopore reads was then mapped to the BioNano single-molecule genomic map using the Bionano Access 1.1.2 and Bionano Solve 3.2 hybrid-scaffolding pipeline with hybrid scaffolding parameters (Non-haplotype without extend and split). After scaffolding, the contig assembly contained 899 scaffolds with a scaffold N50 of 9.94 Mb, gap number was 177, and the proportion of gaps accounted for 6.03% of the whole genome.

To fill the gaps in the scaffolds, the pipline[17] (-minMatch 8 -sdpTupleSize 8 -minPctIdentity 75 -bestn 1 -nCandidates 10 -maxScore -500 －noSplitSubreads) was used to map the Nanopore long reads to the genome assembly scaffolding with BioNano optical mapping. Reads from the Illumina DNA library (400bp) were then aligned against the genome assembly using the BWA (BWA, RRID:SCR 010910) and the genome was polished using Pilon 1.22 once again with default parameters, yielding a final draft genome of approximately 574.62 Mb, with only 164 gaps, gap length for 5.65% of the genome, and contig and scaffold N50 sizes of 4.34 Mb and 16.43 Mb,

respectively (Supplementary Table S7). Because of the advantages of Nanopore sequencing technology and Bionano sequencing technology, the assembly quality of *J. sigillata* genome assembly is currently far superior to reference genomes of its close relatives (Table 1).

Genome quality evaluation

To assess the completeness of the assembled *J. sigillata* genome, we performed Benchmarking Universal Single-Copy Orthologs (BUSCO, RRID: SCR_015008) analysis[18] by searching against the embryophyta BUSCO (version 3.0). Among 1,440 total BUSCO groups searched, 1,341 and 19 BUSCO core genes were completed and partially identified, respectively, leading to a total of 93.1% BUSCO genes in *J. sigillata* genome (Supplementary Table S8). In concert we checked whether the high duplication rate (10.5%) indicated allelic duplications in the assembled genome, using BWA to align and counting up the coverage statistics from the Illumina short reads[19]. The sequencing coverage of the duplicated genes is almost the same as that of single-copy genes (Supplementary Figure S3), showing that these duplicated genes likely exist as independent and distinct copies in the genome.

Chromosome assembly using Hi-C data

To further generate a chromosomal level assembly of the genome, we took advantage of sequencing data from the Hi-C library [20,21]. We performed quality control of Hi-C raw data using HiC-Pro (v. 2.8.0; HiC-Pro, RRID:SCR_017643)[22]. First, we used bowtie2 (v. 2.2.5, Bowtie , RRID:SCR_005476)[23] to compare the raw reads to the draft assembled sequence, and then low-quality reads were filtered out to build raw inter /

intra-chromosomal contact maps. Our final valid data set was 21.31 Gb (37.13×), accounting for 28.46% of the total Hi-C sequencing data. We then used the LACHESIS pipeline (LACHESIS, RRID:SCR_017644) [24] to scaffold *J. sigillata* genome to 16 pseudochromosomes with length ranging from 10.00 Mb to 55.29 Mb. The total length of pseudochromosomes consisted of 93.0% of all genome sequences (Supplementary Figure S4, Supplementary Table S9).

Genome annotation

To identify known transposable elements (TEs) in the *J. sigillata* genome, RepeatMasker (RepeatMasker, RRID:SCR 012954)[25] was used to screen the assembled genome against the Repbase (v. 22.11)[26] and Mips-REdat libraries[27]. In addition, *de novo* repeat annotation was performed using RepeatModeler v. 1.0.11 (RepeatModeler, RRID:SCR 015027) [25]. The combined results of the homology-based and *de novo* predictions indicated that repeated sequences account for 50.06% of the *J. sigillata* genome assembly, with long terminal repeats accounting for the greatest proportion (21.42%) (Supplementary Table S10 and Figure 1).

Homology-based ncRNA annotation was performed by mapping plant rRNA, miRNA, and snRNA genes from the Rfam database (release 13.0) [28] to the *J. sigillata* genome using BLASTN (BLASTN, RRID:SCR_001598) [29] (E-value ≤ 1e−5). tRNAscan-SE v1.3.1 (tRNAscan-SE, RRID:SCR 010835) [30] was used (with default parameters for eukaryotes) for tRNA annotation. RNAmmer v1.2[31] was used to predict rRNAs and their subunits. These analyses identified 311 miRNAs, 807 tRNAs, 151 rRNAs, and 1,171 snRNAs (Supplementary Table S11).

To annotate genes in the *J. sigillata* genome, gene prediction was performed with homology-based, de novo, and transcriptome sequencing-based methods. For homology-based predictions, protein sequences from five species (*A. thaliana*, *E.guineensis*, *O.europaea*, *J.regia*, *P.trichocarpa*) were mapped onto the *J. sigillata* genome using tBLASTn with an E-value of “1e-5”; the aligned sequences and the corresponding query proteins were then filtered and passed to GeneWise v2.4.1 (GeneWise, RRID:SCR 015054)[32] to search for accurately spliced alignments. For the *de novo* predictions, we first randomly selected 1,000 full-length genes from the homology-based predictions to train model parameters for Augustus v3.0 (Augustus: Gene Prediction, RRID:SCR 008417) [33], Genemark[34], GlimmerHMM (GlimmerHMM, RRID:SCR 002654)[35]. Augustus v3.0, Genemark and GlimmerHMM, were then used to predict genes based on the training set. We also used NGS transcriptome short reads aligned on the *J. sigillata* genome using the TopHat (TopHat, RRID:SCR_013035) package[36]. Finally, EVidenceModeler v1.1.1[37] was used to integrate the predicted genes and generate a consensus gene set. Genes with TEs were discarded using the TransposonPSI [38] package. Low quality genes consisting of fewer than 50 amino acids and/or exhibiting premature termination (by aligning codons one by one, the fragments with termination codons in the middle) were also removed from the gene set, yielding a final set of 30,387genes. The final set’s average transcript length, average CDS length, exon number per gene, average exon length and average intron length were 4,687.32 bp, 1,257.18 bp, 5.49, 228.82 bp, and 763.25 bp, respectively (Supplementary Table S12 and Figure 1).

The annotations of the predicted genes of *J. sigillata* were screened for homology against the Uniprot database (accessed 31 January 2018), KEGG database (accessed 87 July 2018) and InterPro database (5.21–60.0) using BLASTX (E value setting of 1e-5, coverage ≥ 50%, and identity ≥ 30% in BLAST v. 2.7.1+)[39], KAAS[40] and InterProScan package (release 5.2–45.0)[41]. In total, most (30,339) of the 30,387 genes were annotated by at least one database, representing 99.8% of the total genes (Supplementary Table S13).

Phylogenetic tree construction and divergence time estimation

The detected *J. sigillata* genes were clustered in families using OrthoMCL (v2.0.9) pipeline (OrthoMCL DB: Ortholog Groups of Protein Sequences, RRID:SCR_007839)[42], with an E-value cutoff of 1e-5, and Markov Chain Clustering with a default inflation parameter in an all-to-all BLASTP analysis of entries for 13 species (*A.thaliana*, *B.pendula*, *C.mollissima*, *C.nucifera*, *E.guineensis*, *J.curcas*, *J.regia*, *O.europaea*, *P.trichocarpa*, *R.communis*, *S.indicum*, *S.lycopersicum*, *V.vinifera*). Gene family clustering identified 16,438 gene families containing 26,539 genes in *J. sigillata*. Of these, 141 gene families were unique to *J. sigillata* (Supplementary Table S14). Phylogenetic analysis was performed using 296 single-copy orthologous genes from common gene families found by OrthoMCL[42]. We codon-aligned each gene family using Mafft (MAFFT, RRID:SCR_011811)[43] and curated the alignments with Gblocks v0.91b (Gblocks, RRID:SCR_015945)[44]. Phylogeny analysis was performed using RAxML (RAxML, RRID:SCR_006086) v 8.2.11[45] with the GTRGAMMA model and 100 bootstrap replicates. We then used

MCMCTREE as implemented in PAML v4.9e (PAML, RRID:SCR_014932) [46] to estimate the divergence times of *J. sigillata* from the other plants. The parameter settings of MCMCTREE were as follows: clock = 2, RootAge ≤ 1.8, model = 7, BDparas = 110, kappa_gamma = 62, alpha_gamma = 11, rgene_gamma = 25.427, and sigma2_gamma = 11.03. In addition, the divergence times of *Vitis vinifera* (110–124 Mya) and *Arabidopsis thaliana* (53–82 Mya) were used for fossil calibrations. The phylogenetic analysis showed that *J. sigillata*, *J.curcas*, and *B.pendula* diverged from a common ancestor approximately 69.41 million years ago. And the estimated divergence time of *J. sigillata* and *J. regia* was 49.49 Mya (Figure 2).

Genes under positive selection

J. sigillata is an important cultivated tree that could be found growing on mountain slopes in Southern China and in the Yunnan-Guizhou Plateau[47]. To evaluate adaptive evolution in the *J. sigillata* genome, we performed analysis to identify genes that are under positive selection. According to the neutral theory of molecular evolution[48], the ratio of nonsynonymous substitution rate (Ka) and synonymous substitution rate (Ks) of protein coding genes can be used to identify genes that show signatures of natural selection. We calculated average Ka/Ks values and conducted the branch-site likelihood ratio test using Codeml implemented in the PAML package (PAML , RRID:SCR_014932)[49] to identify positively selected genes in the *J. sigillata* lineage. Twenty-five genes with signatures of positive selection were identified ($P \leq 0.05$), of which 20 genes could be annotated with potential functions in the Swissprot database (Additional file 2). Gene ontology (GO) analysis using DAVID program

(https://david.ncifcrf.gov/) ($P \leq 0.05$) showed that six of these genes were related to chloroplast activity or function, and these six genes were Ultraviolet-B receptor UVR8 (*UVR8*), Carbamoyl-phosphate synthase large chain (*CARB*), PsbP domain-containing protein 6 (*PPD6*), Probable N-acetyl-gamma-glutamyl-phosphate reductase (*At2g19940*), Beta-carotene isomerase D27(*D27*) and Omega-amidase (*NLP3*). UVR8 was a photoreceptor for ultraviolet-B. Upon ultraviolet-B irradiation, UVR8 underwent an immediate switch from homodimer to monomer, which triggered a signaling pathway for ultraviolet protection[50]. CARB is involved in arginine biosynthesis, and required for mesophyll development[51]. PPD6 is an important protein involved in the redox regulation of photosystem II[52]. D27 was an iron binding protein that localizes in chloroplasts, required for the biosynthesis of strigolactones[53]. NLP3 involved in the metabolism of asparagine, probably also closely coupled with glutamine transamination in the methionine salvage cycle, can use alpha-ketosuccinamate and alpha-hydroxysuccinamate as substrates, producing respectively oxaloacetate and malate, or alpha-ketoglutaramate, producing alpha-ketoglutarate[54]. In conclusion, the functions of these genes were closely related to systems including chloroplast defense mechanisms, photosynthesis, amino acid metabolism, which might help *J. sigillata* adapt to the strong ultraviolet and high-altitude environment of the Yunnan plateau.

Gene family expansion and contraction analysis

To understand the relationships of the *J. sigillata* gene families with those of other plants, we performed a systematic comparison of genes among different species. The

protein-coding genes of 13 genomes, namely, *A. thaliana*, *B. pendula*, *C. nucifera*, *C. mollissima*, *E. guineensis*, *J. curcas*, *J. regia*, *O. europaea*, *P. trichocarpa*, *R. communis*, *S. indicum*, *S. lycopersicum*, *V. vinifera*, were used for the comparison. Gene loss and gain are among the primary reasons for functional changes. To gain greater insights into the evolutionary dynamics of the genes, we determined the expansion and contraction of the orthologous gene clusters in these 14 species with CAFE software (CAFE, RRID: SCR_005983)[55]. This approach revealed 529 expanded gene families and 573 contracted gene families in *J. sigillata* lineage (Figure 3, Additional file 3). Further, enrichment pipeline software clusterProfiler[56] (clusterProfiler, RRID:SCR_016884) was used to test the statistical enrichment of expanded and contracted gene families in KEGG and GO pathway analysis. Pathways with Q-value < 0.05 (Q-values are the name given to the adjusted P-values found using an optimized FDR approach[57]) were considered to be significantly enriched. There were no statistically significant enrichments in KEGG and GO analysis of the contracted gene families (Q-value > 0.05). The expanded gene families were enriched for 87 significant (Q-value <0.05) GO terms at level 4 (Additional file 3). The significantly enriched KEGG pathways included ‘plant-pathogen interactions’ (65, 12.29%), ‘mRNA surveillance pathway’ (44, 8.31%), ‘Phospholipase D signaling pathway’ (31, 5.86%), ‘Fc gamma R-mediated phagocytosis’ (31, 5.86%) and ‘cAMP signaling pathway’ (31, 5.86%) (Additional file 3 and Supplementary Figure S5).

Conclusion

This paper reports a chromosome-level reference genome sequence of *J. sigillata* using

multiple types of sequencing data and assembly technologies. The assembled highly accurate genome will provide a valuable resource for studying the species' evolutionary history, genetic changes and associated biological phenomena, such as genetic load and selection pressures that occurred during severe bottlenecks or other unknown historical events. The *J. sigillata* genome lays a solid foundation for additional genomic studies in nut crops and related species, as well providing valuable resources for plant breeders.

Availability of supporting data

The raw sequence data and *J. sigillata* genome data have been deposited in the Short Read Archive under NCBI BioProject ID PRJNA509030. The genome assembly, annotations, and other supporting data are available via the *GigaScience* database GigaDB [58].

Additional files

Additional file 1: The genome survey in the related species.

Additional file 2: The genes under positive selection.

Additional file 3: The KEGG and GO pathway analysis of expanded and contracted gene families.

Supplementary file: A Word file with Tables S1-S14 and Fig. S1–S5.

Abbreviations

bp: base pair; BLAST: Basic Local Alignment Search Tool; BUSCO: Benchmarking Universal Single-Copy Orthologs; Gb: giga base; GO: gene ontology; KEGG: Kyoto Encyclopedia of Genes and Genomes; Hi-C: high-throughput chromosome

conformation capture; KAAS: KEGG Automatic Annotation Server; kb: kilo base; Mb: mega base; TE: transposable element.

Competing interests

The authors declare that they have no competing interests.

Funding

This work was financially supported by the Yunnan Provincial Science and Technology Major Project (2018ZG001 and 2018ZG002), the Science and Technology Innovation Program of Forestry Department of Yunnan Province ([2014]cx01 and [2016]cx03).

Author contributions

F. C., D. N., and T. W. designed the study and contributed to the project coordination.; L. X., T. W., T. M., W. F. and R. D. collected the sample and extracted the genomic DNA. T. W., L. X., and T. M. performed research and/or analyzed data. T. W. wrote the manuscript. All authors reviewed the manuscript.

Acknowledgements

We are grateful to Nextomics Biosciences Institute (Wuhan, Hubei, China) for providing Genome sequencing, assembly and annotation, and thank Mingfei Zhu and Zongyi Sun for their help revising the manuscript.

References

1. McGranahan G, Leslie C. Walnut. In: Badenes M, Byrne D. Fruit Breeding. Handbook of Plant Breeding, vol. 8. Springer, Boston, MA. 2012. p. 827-46.
2. Lu A, Stone DE, Grauke LJ. Juglandaceae. In: Wu ZY and Raven PH. Flora of China, vol. 4. Missouri Botanical Garden Press, St. Louis, Missouri. 1999. p. 277–85.
3. Zhang Y, Dong RQ, Xi XL. Germplasm Resource of Walnut in Yunnan and Its Exploitation and Utilization. Journal of Northwest Forestry University 2004;19(2):38-40.
4. Ministry of Forestry. China forestry statistical yearbook. Beijing: China Forestry Publishing House; 2017. p. 85-91.

5. Woodworth RH. Meiosis of microsporogenesis in the Juglandaceae. Am J Bot 1930; 17(9):863-9.
6. Chen LN , Ma QG , Chen YK, et al. Identification of major walnut cultivars grown in China based on nut phenotypes and SSR markers. Sci Hortic 2014;168:240-8.
7. Britton MT, Leslie CA, Caboni E, et al. Persian Walnut. In: Chittaranjan K and Timothy CH. Compendium of transgenic crop plants: transgenic temperate fruits and nuts. Wiley-Blackwell, Massachusetts. 2008. p.189-232.
8. MartínezGarcía PJ, Crepeau MW, Puiu D, et al. The walnut (*Juglans regia*) genome sequence reveals diversity in genes coding for the biosynthesis of non-structural polyphenols. Plant J 2016, 87(5):507-32.
9. Senol Cali D, Kim JS, Ghose S, et al. Nanopore sequencing technology and tools for genome assembly: computational analysis of the current state, bottlenecks and future directions. Brief Bioinform 2018:1-18; doi:10.1093/bib/bby017.
10. Marcais G and Kingsford C. A fast, lock-free approach for efficient parallel counting of occurrences of k-mers. Bioinformatics 2011;27(6):764-70.
11 Liu MJ, Zhao J, Cai QL, et al. The complex jujube genome provides insights into fruit tree biology. Nat Commun 2014;5:5315.
12 Kajitani R, Toshimoto K, Noguchi H, et al. Efficient de novo assembly of highly heterozygous genomes from whole-genome shotgun short reads. Genome Res 2014;24(8):1384-95.
13. Koren S, Walenz BP, Berlin K, et al. Canu: scalable and accurate long-read assembly via adaptive k-mer weighting and repeat separation. Genome Res 2017; 27(5):722-36.
14. WTDBG package：https://github.com/ruanjue/wtdbg. (Accessed 10 Jan 2018).
15. Loman NJ, Quick J and Simpson JT. A complete bacterial genome assembled de novo using only nanopore sequencing data. Nat Methods 2015;12:733.
16. Walker BJ, Abeel T, Shea T, et al. Pilon: an integrated tool for comprehensive microbial variant detection and genome assembly improvement. PloS one 2014;9(11): e112963.
17. Chaisson MJ and Tesler G. Mapping single molecule sequencing reads using basic local alignment with successive refinement (BLASR): application and theory. BMC Bioinformatics 2012;13:238.
18. Simão FA, Waterhouse RM, Ioannidis P, et al. BUSCO: assessing genome assembly and annotation completeness with single-copy orthologs. Bioinformatics 2015;31(19):3210-2.
19 Kang K, Bergdahl B, Machado D, et al. Linking genetic, metabolic, and phenotypic diversity among *Saccharomyces cerevisiae* strains using multi-omics associations. GigaScience 2019;8(4):giz015. https://academic.oup.com/gigascience/article-abstract/8/4/giz015/5304885
20. Dudchenko O, Batra SS, Omer AD, et al. De novo assembly of the *Aedes aegypti* genome using Hi-C yields chromosome-length scaffolds. Science 2017;356(6333):92-5.
21. Belton JM, McCord RP, Gibcus JH, et al. Hi-C: a comprehensive technique to capture the conformation of genomes. Methods 2012;58(3):268-76.
22. Servant N, Varoquaux N, Lajoie BR, et al. HiC-Pro: an optimized and flexible pipeline for Hi-C data processing. Genome Biol 2015;16:259.
23. Langmead B and Salzberg SL. Fast gapped-read alignment with Bowtie 2. Nat Methods 2012;9(4): 357-9.
24. Korbel JO and Lee C. Genome assembly and haplotyping with Hi-C. Nat Biotechno 2013;31(12):1099-101.
25. Tarailo-Graovac M and Chen N. Using RepeatMasker to identify repetitive elements in genomic sequences. Curr Protoc Bioinformatics 2009;25(1):4.10.1–4.10.14.
26. Bao W, Kojima KK and Kohany O. Repbase Update, a database of repetitive elements in eukaryotic genomes. Mobile DNA 2015;6(1):11.
27. Nussbaumer T, Martis MM, Roessner SK, et al. MIPS PlantsDB: a database framework for

comparative plant genome research. Nucleic Acids Res 2013;41 Database issue:D1144-D51.
28. Kalvari I, Argasinska J, Quinones-Olvera N, et al. Rfam 13.0: shifting to a genome-centric resource for non-coding RNA families. Nucleic Acids Res 2018;46 D1:D335-D42.
29. Camacho C, Coulouris G, Avagyan V, et al. BLAST+: architecture and applications. BMC Bioinformatics 2009;10:421.
30. Lowe TM and Eddy SR. tRNAscan-SE: a program for improved detection of transfer RNA genes in genomic sequence. Nucleic Acids Res 1997;25(5):955-64.
31. Lagesen K, Hallin P, Rodland EA, et al. RNAmmer: consistent and rapid annotation of ribosomal RNA genes. Nucleic Acids Res 2007;35(9):3100-8.
32. Birney E and Durbin R. Using GeneWise in the Drosophila annotation experiment. Genome Res 2000;10(4):547-8.
33. Stanke M, Steinkamp R, Waack S et al. AUGUSTUS: a web server for gene finding in eukaryotes. Nucleic Acids Res 2004;32 Web Server issue:W309-12.
34. Blanco E, Parra G and Guigó R. Using geneid to identify genes. Curr Protoc Bioinformatics 2007;18(1): 4.3.1-4.3.28.
35. Majoros WH, Pertea M and Salzberg SL. TigrScan and GlimmerHMM: two open source ab initio eukaryotic gene-finders. Bioinformatics 2004;20(16):2878-9.
36. Trapnell C, Pachter L, Salzberg SL. TopHat: discovering splice junctions with RNA-Seq. Bioinformatics 2009; 25(9): 1105-1111.
37. Haas BJ, Salzberg SL, Wei Z, et al. Automated eukaryotic gene structure annotation using EVidenceModeler and the Program to Assemble Spliced Alignments. Genome Biol 2008;9(1):R7.
38. TransposonPSI: An Application of PSI-Blast to Mine (Retro-)Transposon ORF Homologies. http://transposonpsi.sourceforge.net/, Accessed 18 Mar 2018.
39 Altschul, SF, Gish W, Miller W, et al. Basic Local Alignment Search Tool. J Mol Biol 2008; 215(3):403-10.
40 Moriya Y, Itoh M, Okuda S, et al. KAAS: an automatic genome annotation and pathway reconstruction server. Nucleic Acids Res 2007;35 Web Server issue:W182-5.
41. Quevillon E, Silventoinen V, Pillai S, et al. InterProScan: protein domains identifier. Nucleic Acids Res 2005;33 Web Server issue:W116-20.
42. Li L, Stoeckert Jr. CJ, Roos DS. OrthoMCL: identification of ortholog groups for eukaryotic genomes. Genome Res 2003;13(9):2178-89.
43. Katoh K and Standley DM. MAFFT multiple sequence alignment software version 7: improvements in performance and usability. Mol Biol Evol 2013;30(4):772-80.
44. Talavera G and Castresana J. Improvement of phylogenies after removing divergent and ambiguously aligned blocks from protein sequence alignments. Syst Biol 2007;56(4):564-77.
45. Stamatakis A. RAxML version 8: a tool for phylogenetic analysis and post-analysis of large phylogenies. Bioinformatics 2014;30(9):1312-3.
46. Yang Z. Paml 4: phylogenetic analysis by maximum likelihood. Mol Biol Evol 2007;24(8):1586–91.
47 Gunn BF, Aradhya M, Salick JM, et al. Genetic variation in walnuts (*Juglans regia* and *J. sigillata*; Juglandaceae): Species distinctions, human impacts, and the conservation of agrobiodiversity in Yunnan, China. Am J Bot 2010; 97(4): 660–71.
48. Gillespie JH. The status of the neutral theory: the neutral theory of molecular evolution. Science 1984;224(4650):732-3.
49. Yang Z. PAML 4: phylogenetic analysis by maximum likelihood. Mol Biol Evol 2007;24(8):1586-91.
50 Wu D, Hu Q, Yan Z, et al. Structural basis of ultraviolet-B perception by UVR8. Nature 2012;484 7393:214-9.
51 Mollá-Morales A, Sarmiento-Mañús R, Robles P, et al. Analysis of ven3 and ven6 reticulate mutants reveals the importance of arginine biosynthesis in Arabidopsis leaf development. Plant J

2011; 65(3):335-45.
52 Hall M, Mata-Cabana A, Åkerlund H-E, et al. Thioredoxin targets of the plant chloroplast lumen and their implications for plastid function. Proteomics 2010;10(5): 987-1001.
53 Lin H, Wang R, Qian Q, et al. DWARF27, an iron-containing protein required for the biosynthesis of strigolactones, regulates rice tiller bud outgrowth. Plant Cell 2009;21 5:1512-25.
54 Zhang Q and Marsolais F. Identification and characterization of omega-amidase as an enzyme metabolically linked to asparagine transamination in Arabidopsis. Phytochemistry 2014; 99:36-43..
55. De Bie T, Cristianini N, Demuth JP et al. CAFE: a computational tool for the study of gene family evolution. Bioinformatics 2006; 22(10):1269-71.
56 Yu G, Wang L, Han Y and He Q. clusterProfiler: an R package for comparing biological themes among gene clusters. OMICS: Integr Biol 2012;16(5):284-7.
57 Storey JD. A direct approach to false discovery rates. J R Stat Soc Ser B-Stat Methodol 2002; 64(3):479-98.
58 Ning D; Wu T; Xiao L; Ma T; Fang W; Dong R; Cao F (2020): Supporting data for "Chromosomal-level assembly of Juglans sigillata genome using Nanopore, BioNano and Hi-C analysis" GigaScience Database. http://dx.doi.org/10.5524/100693.

Table 1. Genome summary of *J. sigillata* and closely related species.

Parameter	*Carya illinoinensis* [1]	*Carya cathayensis* [1]	*Quercus lobata*[2]	*Betula pendula*[3]	*Juglans regia*[4]	*Juglans Microcarpa* [4]	*Quercus robur*[5]	*Juglans sigillata*
Estimated genome size (Mb)	649.75	721.33	730	440	---	--	736	618.79
heterozygosity rate	1.46	0.77	1.25	--	--	--	1.52	1.0
Total assembly (Mb)	651.31	706.43	1170	436	534.67	572.90	750	574.62
Contig N50 (Kb)	77.23	101.58	24.31	49.45	15,066.22	11,553.27	69.35	4,336.69
Scaffold N50 (Mb)	1.08	1.22	278.07	0.24	35.20	35.63	1.34	16.43
Contigs / Scaffolds	61,935/43,503	53,100/40,425	-/94,394	27,582/5,644	127/73	208/154	22,615/1,409	913/749
proportion of gaps	--	--	--	--	--	--	2.94	5.65
rate of the anchored assemblies(%)	--	--	--	89	99	99	96	93
Protein-coding genes	31,075	32,907	61,773	28,153	31,425	29,496	25,808	30,387
Repeat sequence (%)	50.43	53.67	52	49.23	44.15	43.88	53.30	50.06
BUSCO (%)	90.5	91.3	88.9	89.5	96.0	95.2	89.2	93.1

-- represents the parameter not reported.

BUSCO v3 was used to assess genome assembly completeness. And datasets based on embryophyta_odb9 (1440 single-copy orthologs).

[1] Huang Y, Xiao L, Zhang Z, et al. The genomes of pecan and Chinese hickory provide insights into *Carya* evolution and nut nutrition. GigaScience 2019;8(5): giz036. https://academic.oup.com/gigascience/article/8/5/giz036/5484800

[2] Sork VL, Fitz-Gibbon ST, Puiu D, et al. First Draft Assembly and Annotation of the Genome of a California Endemic Oak *Quercus lobata* Nee (Fagaceae). G3-Genes Genomes Genetics 2016; 6(11): 3485-

95.
[3] Salojarvi J, Smolander OP, Nieminen K, et al. Genome sequencing and population genomic analyses provide insights into the adaptive landscape of silver birch. Nature Genetics 2017;49(6):904-12.
[4] Zhu T, Wang L, You FM, et al. Sequencing a *Juglans regia* x *J. microcarpa* hybrid yields high-quality genome assemblies of parental species. Horticulture research 2019;6:55.
[5] Plomion C, Aury JM, Amselem J, Leroy T, Murat F, Duplessis S, et al. Oak genome reveals facets of long lifespan. Nature plants 2018;4(7):440-52.

Figure Click here to access/download;Figure;figure.doc

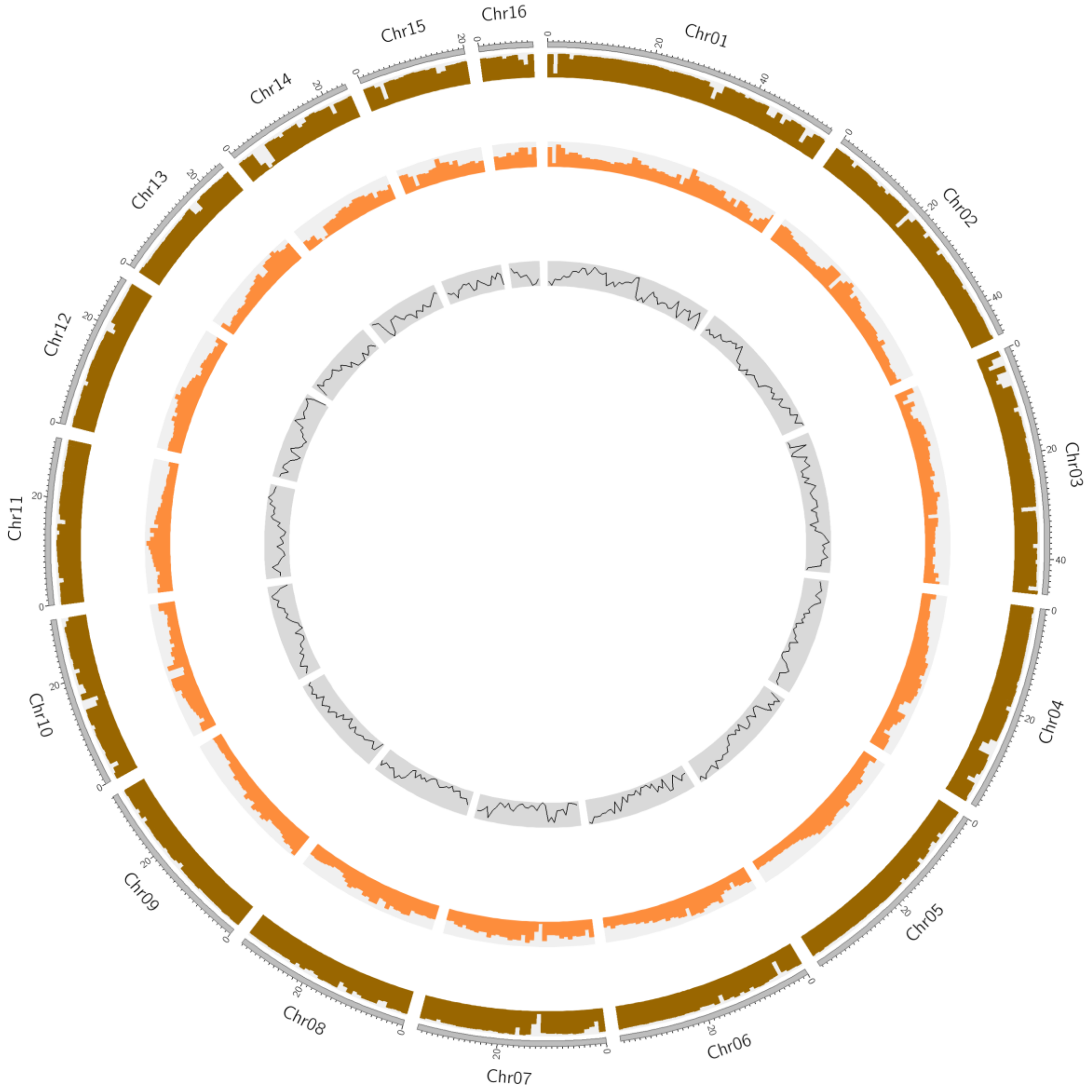

Figure 1. Circular diagram depicting the characteristics of the *J. sigillata* genome. The tracks from outer to inner circles indicate the following: GC density, repeat density and gene density.

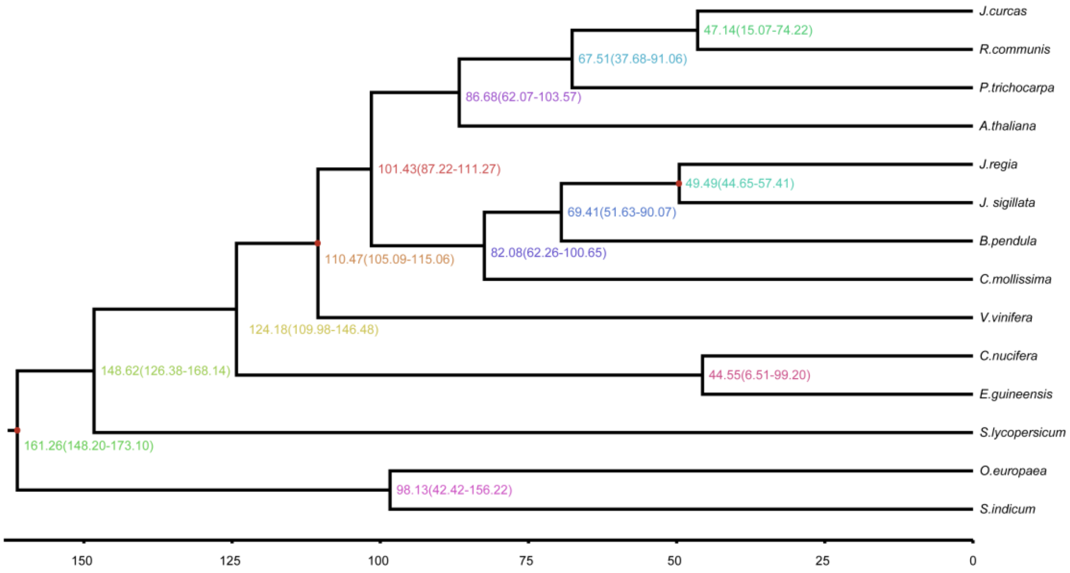

Figure 2. Inferred phylogenetic tree across 14 plant species. The estimated divergence time (Mya) is shown at each node.

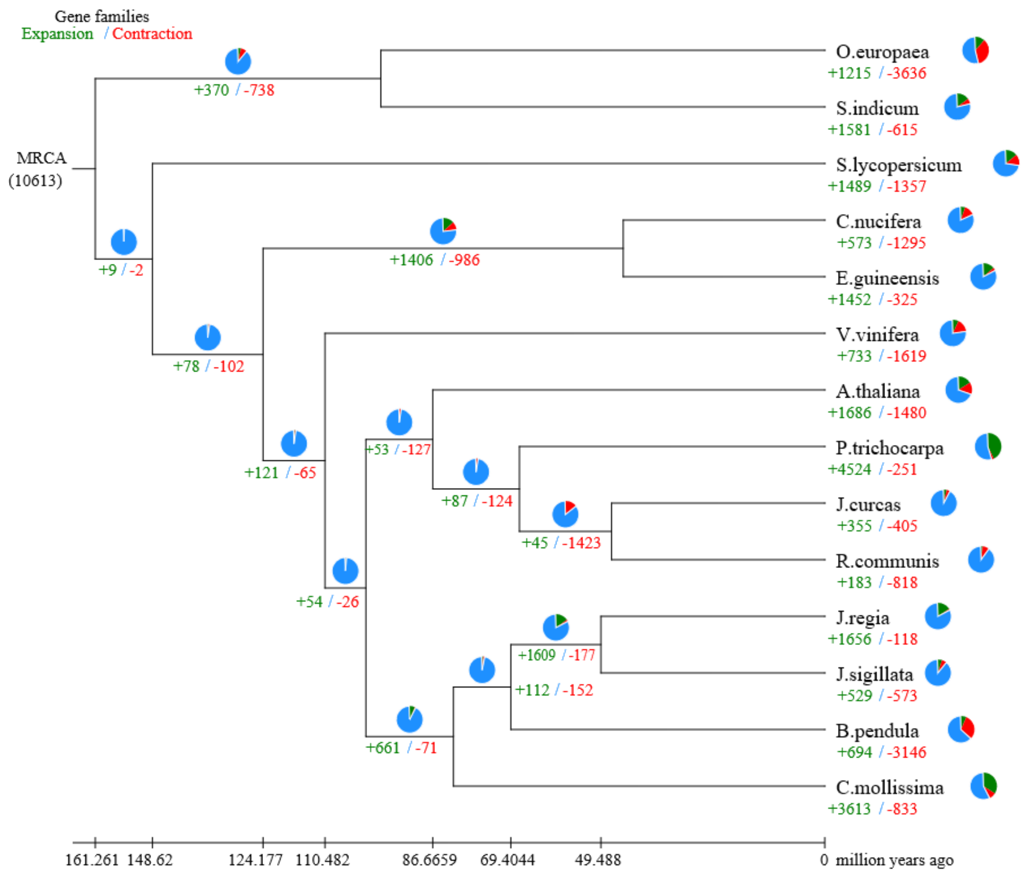

Figure 3. Gene family expansions and contractions in *J. sigillata* and 13 other plants. The pie chart shows the proportion of gene families, expansion gene families (green), contraction gene families (red), and unaltered gene families (blue).

Click here to access/download
Supplementary Material
Supplementary file.docx

Additional file 1

Click here to access/download
Supplementary Material
renamed_f25b4.docx

Additional file 2

Click here to access/download
Supplementary Material
renamed_9fcf1.xlsx

Additional file 3

Click here to access/download
Supplementary Material
renamed_079ea.xlsx

Reviewer reports

Click here to access/download
Supplementary Material
Reviewer reports.docx

Personal Cover

Click here to access/download;Personal Cover;Personal Cover.docx

Chromosomal-level assembly of *Juglans sigillata* genome using Nanopore, BioNano and Hi-C analysis

De-Lu Ning[1,2],† Tao Wu[2,3],† Liang-Jun Xiao[2], Ting Ma[2], Wen-Liang Fang[2], Run-Quan Dong[2], Fuliang Cao[4]*

[1] Central South University of Forestry and Technology, Changsha 410004, China

[2] Institute of Economic Forest, Yunnan Academy of Forestry and Grassland, Kunming 650201, China

[3] Yunnan Laboratory for Conservation of Rare, Endangered & Endemic Forest Plants, Public Key Laboratory of the State Forestry Administration; Yunnan Provincial Key Laboratory of Cultivation and Exploitation of Forest Plants, Kunming 650201, China

[4] Co-Innovation Center for the Sustainable Forestry in Southern China, Nanjing Forestry University, Nanjing 210037, China

* Corresponding author: CFL1957@qq.com

† These authors contributed equally.

De-Lu Ning Email: ningdelu@163.com, ORCID identifier: https://orcid.org/0000-0001-9152-0172

Tao Wu Email: ynafwt@126.com, ORCID identifier: https://orcid.org/0000-0002-5371-9700

Fuliang Cao Email: CFL1957@qq.com, ORCID identifier: https://orcid.org/0000-0002-0594-6968

Abstract

Background: *Juglans sigillata* (NCBI: txid224355), belonging to Juglandales order, is an economically important tree species in Asia, especially in Yunnan province of China. However, little research has been conducted on *J. sigillata* at the molecular level, which hinders understanding of its evolution, speciation, and synthesis of secondary metabolites, as well as its wide adaptability to the plateau environment. To address these issues, a high-quality reference genome of *J. sigillata* would be a very useful resource.

Findings: To construct a high-quality reference genome for *J. sigillata*, we first generated 38.0 Gb short reads and 66.31 Gb long reads using Illumina and Nanopore sequencing platforms, respectively. The sequencing data were assembled into a 536.50 Mb genome assembly with a contig N50 length of 4.31 Mb. Additionally, we applied BioNano technology to identify contacts among contigs, which were then used to assemble contigs into scaffolds, resulting in a genome assembly with scaffold N50 length of 16.43 Mb and contig N50 length of 4.34 Mb. To obtain a chromosome-level genome assembly, we constructed one Hi-C library and sequenced 79.97 Gb raw reads using the Illumina HiSeq platform. We anchored approximately 93% of the scaffold sequences into 16 chromosomes and evaluated the quality of our assembly using the high contact frequency heatmap. Repetitive elements account for 50.06% of the genome, and 30,387 protein-coding genes were predicted from the genome, of which 99.8% have been functionally annotated. The genome-wide phylogenetic tree indicated the divergence time between *J. sigillata* and *J.regia* was estimated to be 49 million years ago (Mya) based on single-copy orthologous genes.

Conclusions: We provide the first chromosome-level genome for *J. sigillata*. The genome will lay a valuable foundation for future research on genetic improvement of *J. sigillata*.

Keywords: *Juglans sigillata*; genome assembly; annotation; evolution
