# Supplementary material for: Chromosomal-level assembly of Juglans sigillata genome using Nanopore, BioNano, and Hi-C analysis
Source: Gigascience. 2020 Feb 26;9(2):giaa006. doi: 10.1093/gigascience/giaa006 (PMC7043058; doi:10.1093/gigascience/giaa006)
Supplement: giaa006_Supplemental_Files [file giaa006_supplemental_files.zip › renamed_f25b4.docx]

| *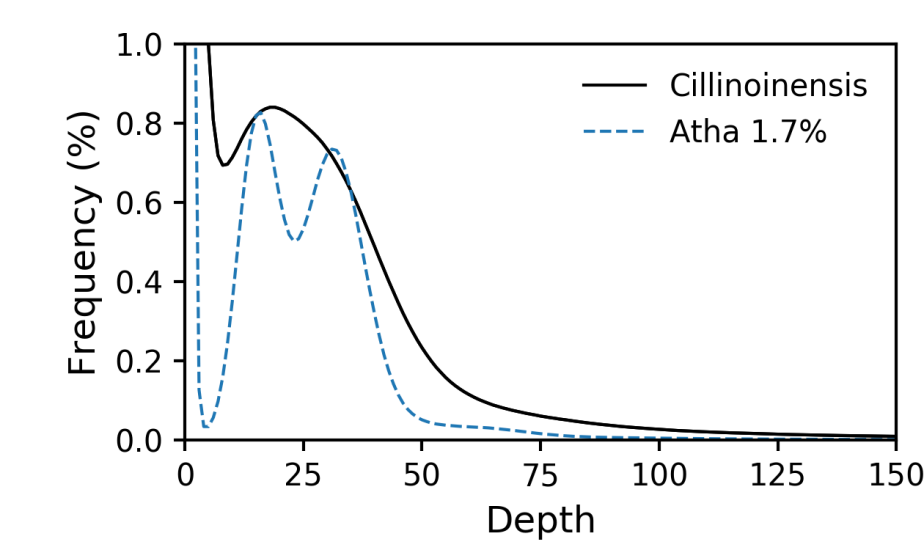* | *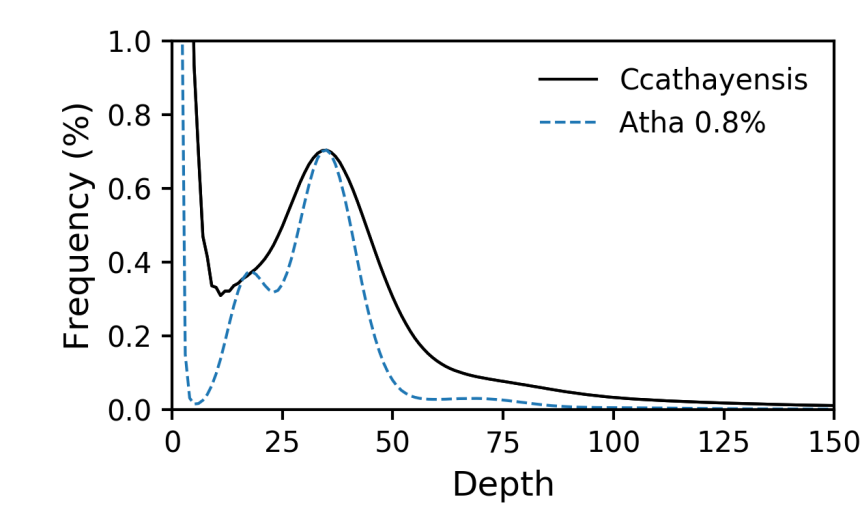* |
| --- | --- |
| (A) *C. illinoinensis* | (B) *C. cathayensis* |
| 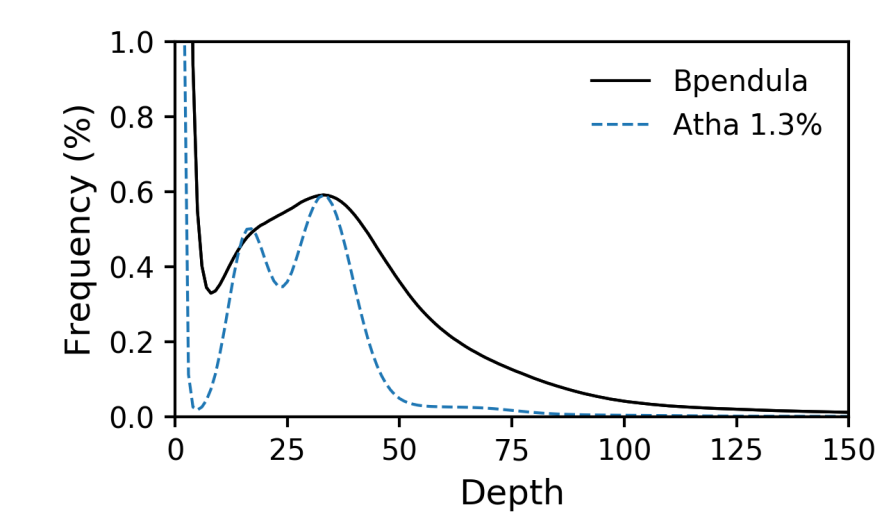 | 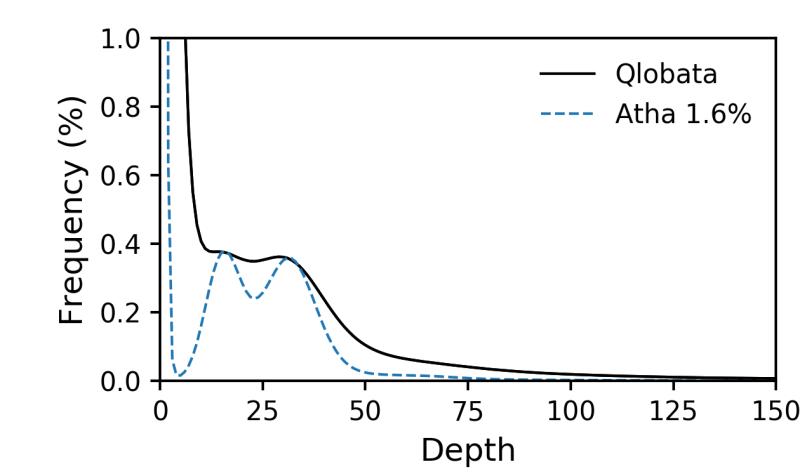 |
| (C) *B. pendula* | (D) *Q. lobata* |

The simulation curve of heterozygosity rate by analyzing kmer frequency

Genome survey of *C. illinoinensis*, *C. cathayensis*, *B. pendula* and *Q. lobate*

| Species | kmer | kmer_num | kmer_depth | genome_size (bp) | Heterozygosity (%) |
| --- | --- | --- | --- | --- | --- |
| *C. illinoinensis* | 17 | 20,119,371,846 | 30 | 670,645,728 | 1.70 |
| *C. cathayensis* | 17 | 31,166,452,128 | 35 | 890,470,060 | 0.80 |
| *B. pendula* | 17 | 19,883,761,917 | 34 | 584,816,526 | 1.30 |
| *Q. lobata* | 17 | 27,121,370,267 | 31 | 874,882,911 | 1.60 |
