# Supplementary material for: Chromosomal-level assembly of Juglans sigillata genome using Nanopore, BioNano, and Hi-C analysis
Source: Gigascience. 2020 Feb 26;9(2):giaa006. doi: 10.1093/gigascience/giaa006 (PMC7043058; doi:10.1093/gigascience/giaa006)
Supplement: giaa006_Supplemental_Files [file giaa006_supplemental_files.zip › Supplementary file.docx]

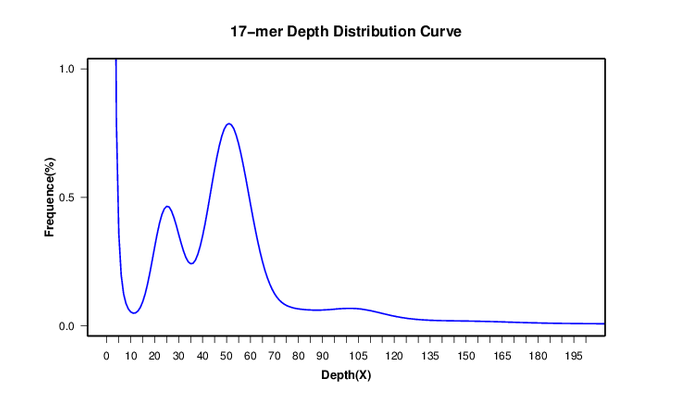


**Figure S1. Frequency distribution of the 17-mer graph analysis uesd to estimate the size of the** ***J. sigillata* genome.**

**
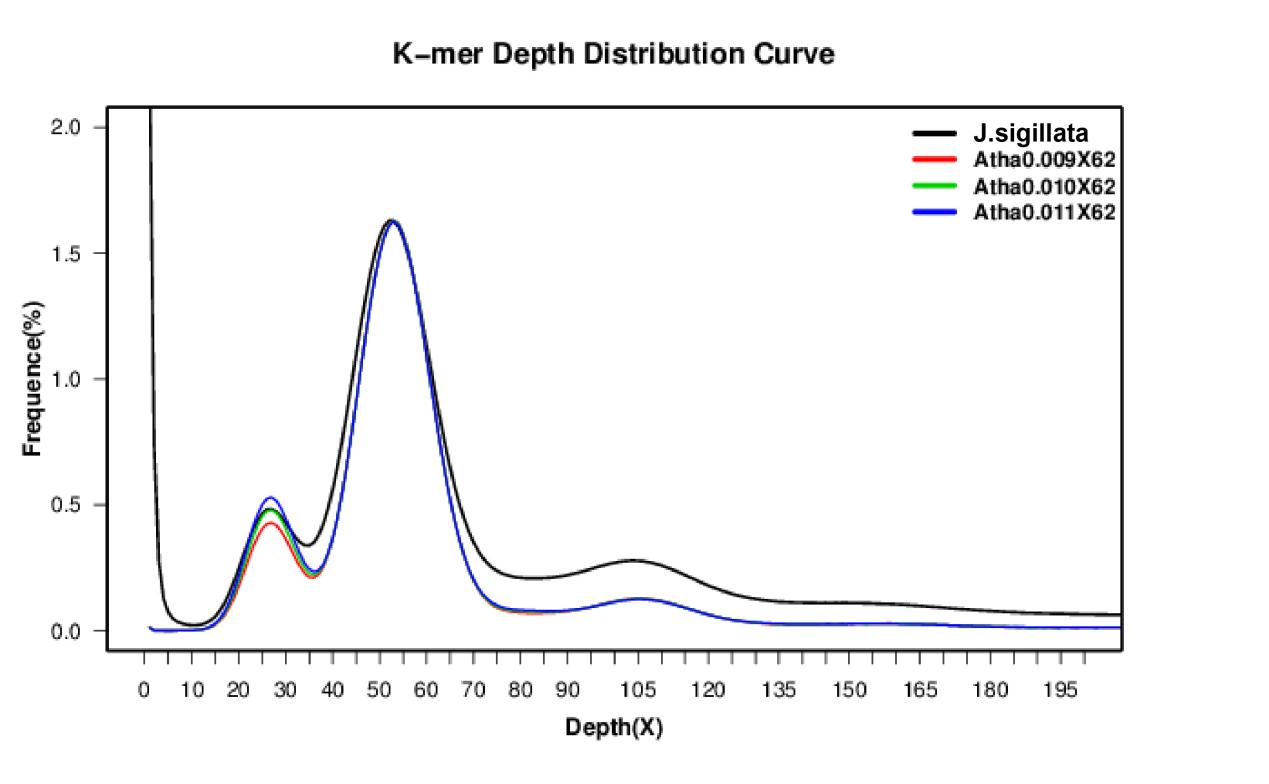
**

**Figure S2. Schematic diagram of simulation curve of *J. sigillata* heterozygosity rate. Atha0.010X62 means *Arabidopsis thaliana* depth heterozygosity (H) 1.0%, (X) depth 62, and so on.**

**
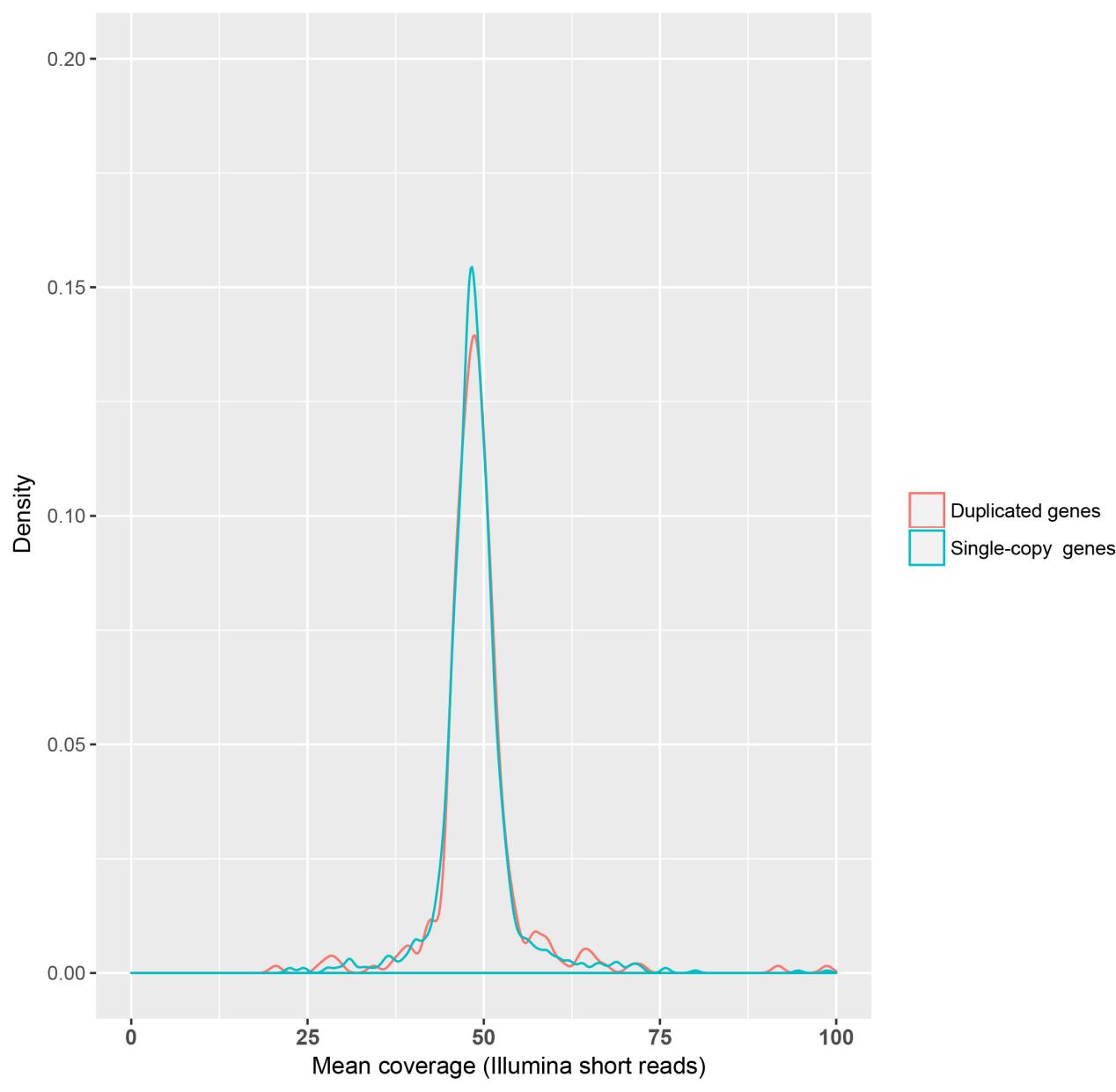
**

**Figure S3. Trendgram of mean coverage (Illumina short reads) of single-copy genes (blue) and duplicated genes (red).**


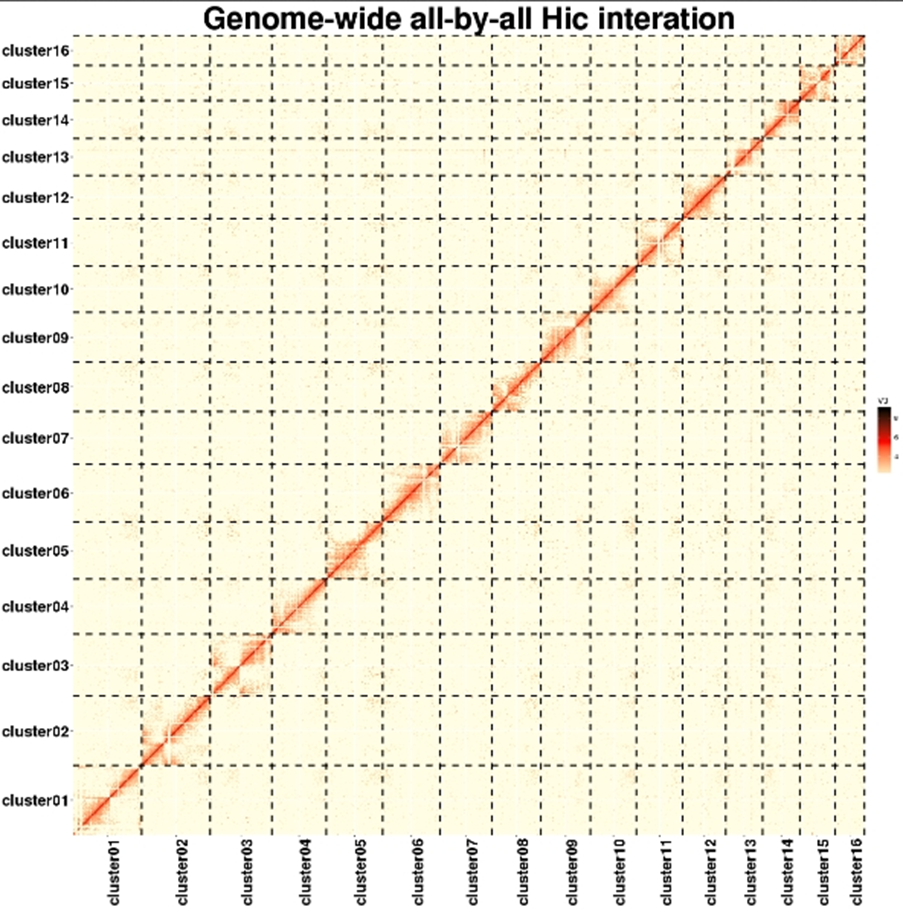


**Figure S4. Interaction frequency distribution of Hi-C links among chromosomes.**

**
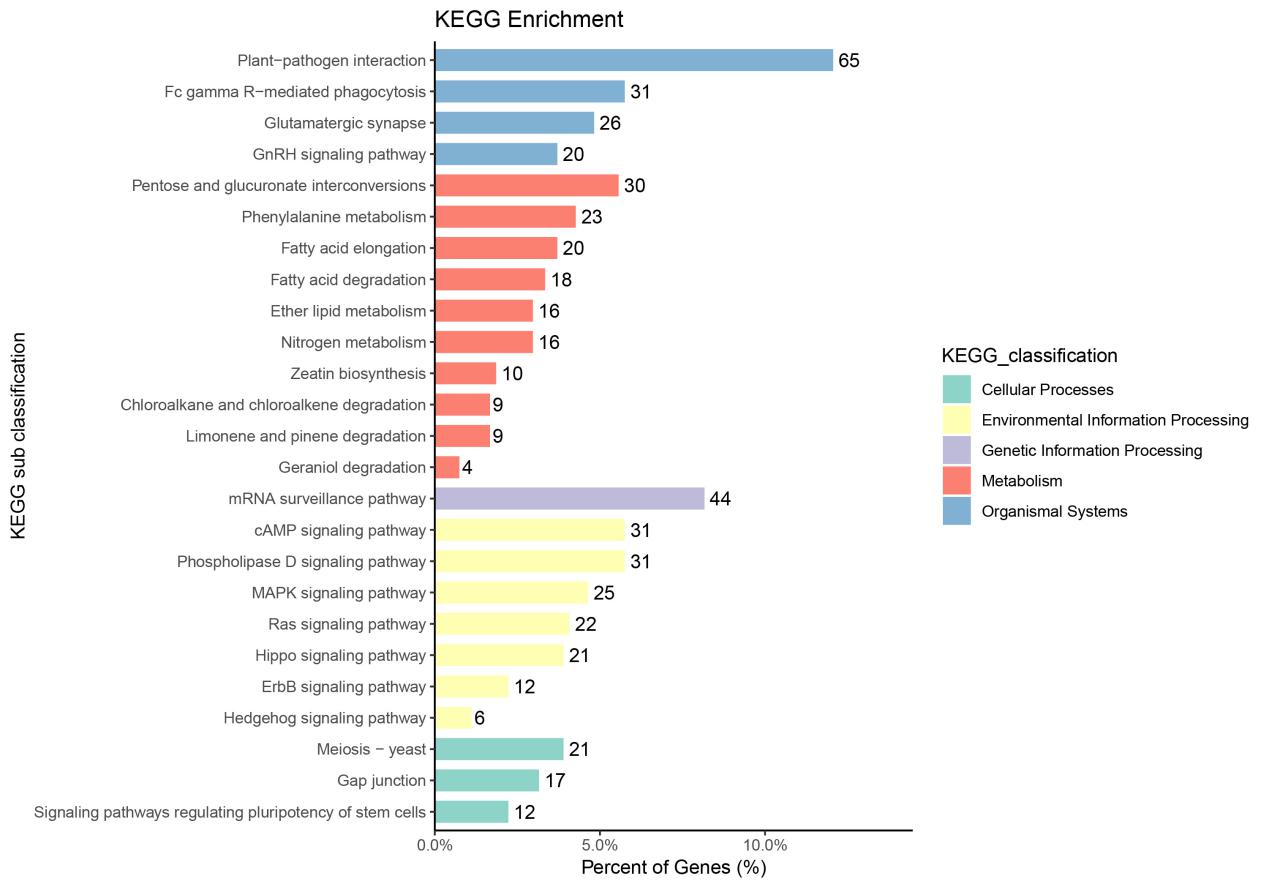
**

**Figure S5. Significantly enriched KEGG pathways of genes in expanded families.**

**Table S1. Summary of Nanopore sequencing**

| **Cells** | **Pass reads Mean** **Length(Kb)** | **Pass reads N50 length(Kb)** | **Pass reads max Length (Kb)** | **raw reads Base(Gb)** | **Coverage** |
| --- | --- | --- | --- | --- | --- |
| 16 | 15.60 | 22.70 | 283 | 66.31 | 115X |

| **Table S2. Summary of Illumina sequencing** | | | | |  |  |
| --- | --- | --- | --- | --- | --- | --- |
| **Reads** | **Bases(bp)** | **Insert_Size(bp)** | **Read_Length(bp)** | **Q30** | **GC** | **Coverage** |
| 253,263,884 | 37,989,582,600 | 400 | 150.00 | 0.93 | 0.36 | 66X |

**Table S3. Estimation of genome size based on 17-mer statistics**

| **kmer** | **kmer_num** | **kmer_depth** | **genome_size(bp)** | **Heterozygosity Rate (%)** |
| --- | --- | --- | --- | --- |
| 17 | 31,558,418,010 | 51 | 618,792,510 | 1.0 |

| **Table S4.** **Statistics of initial assembly results**   \| **Stat Type** \| **Contig Length（bp）** \| **Contig Number** \| \| --- \| --- \| --- \| \| N50 \| 4,257,962 \| 41 \| \| N60 \| 3,337,774 \| 55 \| \| N70 \| 2,376,532 \| 74 \| \| N80 \| 1,692,656 \| 101 \| \| N90 \| 695,042 \| 146 \| \| Longest \| 15,170,801 \| 1 \| \| Total \| 531,624,529 \| 956 \| \| Length>=1kb \| 531,624,529 \| 956 \| \| Length>=2kb \| 531,624,529 \| 956 \| \| Length>=5kb \| 531,590,627 \| 949 \|   **Table S5. Summary of the polished genome assembly**   \| **Stat Type** \| **Contig Length（bp）** \| **Contig Number** \| \| --- \| --- \| --- \| \| N50 \| 4,307,655 \| 41 \| \| N60 \| 3,377,944 \| 55 \| \| N70 \| 2,398,862 \| 74 \| \| N80 \| 1,712,567 \| 101 \| \| N90 \| 692,841 \| 147 \| \| Longest \| 14,795,211 \| 1 \| \| Total \| 536,502,447 \| 956 \| \| Length>=1kb \| 536,502,447 \| 956 \| \| Length>=2kb \| 536,502,447 \| 956 \| \| Length>=5kb \| 536,493,475 \| 954 \|   **Table S6. Summary of the BioNano optical mapping data** | | |  |
| --- | --- | --- | --- | --- | --- | --- | --- | --- | --- | --- | --- | --- | --- | --- | --- | --- | --- | --- | --- | --- | --- | --- | --- | --- | --- | --- | --- | --- | --- | --- | --- | --- | --- | --- | --- | --- | --- | --- | --- | --- | --- | --- | --- | --- | --- | --- | --- | --- | --- | --- | --- | --- | --- | --- | --- | --- | --- | --- | --- | --- | --- | --- | --- | --- | --- | --- | --- | --- | --- |
| **Clean Data Quantity (Gb)** | **Clean Data Average Label (/100Kb)** | **Clean Data N50 (Kb)** | **Coverage** |
| 149.64 | 7.95 | 264.04 | 260X |

**Table S7. Summary of the final genome assembly**

| **StatType** | **Scaffold length** | **Scaffold number** | **Contig Length** | **Contig number** | **Gap**  **length** | **Gap number** |
| --- | --- | --- | --- | --- | --- | --- |
| N50 | 16434359 | 13 | 4336690 | 40 | 486160 | 20 |
| N60 | 10819456 | 18 | 3098050 | 55 | 375994 | 28 |
| N70 | 8762071 | 24 | 2271033 | 75 | 295064 | 37 |
| N80 | 6510981 | 31 | 1574156 | 103 | 193851 | 52 |
| N90 | 1729665 | 48 | 518691 | 159 | 113409 | 72 |
| Longest | 33527859 | 1 | 14795211 | 1 | 2532649 | 1 |
| Total | 574618720 | 749 | 542130050 | 913 | 32488670 | 164 |
| Length>=1kb | 574618720 | 749 | 542130050 | 913 | 32487129 | 134 |
| Length>=2kb | 574618720 | 749 | 542130050 | 913 | 32484162 | 132 |
| Length>=5kb | 574609748 | 747 | 542121078 | 911 | 32481337 | 131 |

**Table S8. Summary of BUSCO analysis results**

| **Type** | **Number** | **Percent(%)** |
| --- | --- | --- |
| Complete BUSCOs (C) | 1341 | 93.1% |
| Complete and single-copy BUSCOs (S) | 1190 | 82.6% |
| Complete and duplicated BUSCOs (D) | 151 | 10.5% |
| Fragmented BUSCOs (F) | 19 | 1.3% |
| Missing BUSCOs (M) | 80 | 5.6% |
| Total BUSCO groups searched | 1440 | 100% |

**Table S9. Statistics of pseudochromosomes of the *J. sigillata***

| **Chr** | **Size** | **Scaf Num** |
| --- | --- | --- |
| cluster1 | 46,466,594 | 16 |
| cluster2 | 46,482,851 | 14 |
| cluster3 | 55,285,816 | 12 |
| cluster4 | 36,570,044 | 13 |
| cluster5 | 38,410,721 | 8 |
| cluster6 | 38,547,420 | 16 |
| cluster7 | 34,920,256 | 22 |
| cluster8 | 33,169,255 | 7 |
| cluster9 | 33,274,365 | 17 |
| cluster10 | 30,933,901 | 11 |
| cluster11 | 31,730,032 | 8 |
| cluster12 | 29,041,193 | 9 |
| cluster13 | 24,559,460 | 17 |
| cluster14 | 24,994,787 | 11 |
| cluster15 | 99,97,359 | 13 |
| cluster16 | 20,013,179 | 8 |
| Total | 574,618,720 | 203 |

**Table S10. Repeat annotation of the** ***J. sigillata* genome assembly**

| Type | Repbase TEs | | Mips-REdat TEs | | TE proteins | | RepeatModeler | | Combined TEs | | |
| --- | --- | --- | --- | --- | --- | --- | --- | --- | --- | --- | --- |
|  | Length  (Mb) | % in  genome | Length  (Mb) | %in  genome | Length  (Mb) | % in  Genome | Length  (Mb) | % in  genome | Length  (Mb) | | % in  genome |
| DNA | 10.2 | 1.89 | 4.7 | 0.87 | 10.8 | 1.99 | 21.6 | 3.99 | 28.4 | | 5.23 |
| LINE | 15.3 | 2.82 | 6.8 | 1.26 | 35.1 | 6.48 | 29.5 | 5.44 | 40.8 | | 7.53 |
| LTR | 48.8 | 9 | 56.7 | 10.46 | 53.4 | 9.85 | 60.9 | 11.23 | 11.6 | | 21.42 |
| SINE | 0 | 0 | 0 | 0 | 0 | 0 | 0.3 | 0.05 | 0.3 | | 0.05 |
| Other | 2.5 | 0.47 | 0.7 | 0.12 | 0.6 | 0.11 | 1 | 0.18 | 34 | | 6.26 |
| Unknown | 0.03 | 0.01 | 0 | 0 | 0 | 0 | 0.08 | 15.01 | 51.9 | | 9.57 |
| Total | 76.9 | 14.19 | 68.9 | 12.71 | 99.9 | 18.43 | 194.6 | 35.9 | 271.4 | | 50.06 |
| DNA, DNA transposon; LINE, long interspersed nuclear element; TEs, transposable elements; | | | | | | | | | |  |  |
| SINE, short interspersed nuclear element; LTR, long terminal repeat. | | | | | | | | | |  |  |

**Table S11. Summary of non-protein-coding gene annotations in the *J. sigillata* genome assembly**

| Type | Copy Number | Average Length (bp) | Total Length (bp) | Percentage of Genome (%) |
| --- | --- | --- | --- | --- |
| **rRNA**  18S  28S  5.8S  5S | **151** | **1,081.01** | **163,233** | **0.02** |
|  | 27 | 1,626.48 | 43,915 | 0.006 |
|  | 20 | 5,343.65 | 106,873 | 0.01 |
|  | 19 | 148.74 | 2,826 | 0.0004 |
|  | 85 | 113.16 | 9,619 | 0.001 |
| **snRNA**  CD-box  HACA-box  splicing | **1,171** | **106.98** | **125,278** | **0.02** |
|  | 903 | 101.41 | 91,569 | 0.01 |
|  | 134 | 114.76 | 15,378 | 0.002 |
|  | 134 | 136.8 | 18,331 | 0.002 |
| **miRNA** | **311** | **124.2** | **38,626** | **0.005** |
| **tRNA** | **807** | **74.69** | **60,274** | **0.008** |

**Table S12. Comparative gene statistics**

| Specie | Total number of gene | Average transcript length(bp) | Average CDS length(bp) | Average exons number per gene | Average exon length(bp) | Average intron length(bp) |
| --- | --- | --- | --- | --- | --- | --- |
| *J. sigillata* | 30,387 | 4,687.32 | 1,257.18 | 5.49 | 228.82 | 763.25 |
| *A.thaliana* | 27,444 | 1,857.35 | 1,205.78 | 5.09 | 236.78 | 159.22 |
| *E.guineensis* | 26,258 | 7,842.84 | 1,322.93 | 5.53 | 239.25 | 1,439.43 |
| *O.europaea* | 40,041 | 3,698.0 | 1,163.69 | 4.78 | 243.41 | 670.31 |
| *J.regia* | 36,861 | 4,091.9 | 1,221.74 | 4.86 | 251.17 | 742.75 |
| *P.trichocarpa* | 31,543 | 3,395.84 | 1,386.57 | 5.55 | 249.85 | 441.63 |

**Table S13. Functional annotation of predicted genes of *J. sigillata***

| **Type** | | **Number** | **Percent (%)** |
| --- | --- | --- | --- |
| **Annotation** | **Swissprot** | 23,844 | 78.5 |
|  | **Trembl** | 28,217 | 92.9 |
|  | **Kegg** | 10,171 | 33.5 |
|  | **GO** | 16,933 | 55.7 |
|  | **KOG** | 16,163 | 53.2 |
|  | **InterProscan** | 24,579 | 80.9 |
| **Total** | **Annotated** | 30,339 | 99.8 |
|  | **Unannotated** | 48 | 0.2 |
|  | **Gene** | 30,387 | - |

**Table S14. Summary statistics of gene families in 14 plant species**

| Species | Genes Number | Genes number in  families | Unclustered genes number | Family number | Unique  Families number | Average genes number per family |
| --- | --- | --- | --- | --- | --- | --- |
| *A.thaliana* | 27,417 | 23,261 | 4,156 | 12,810 | 740 | 1.82 |
| *B.pendula* | 23,950 | 19,067 | 4,883 | 12,695 | 373 | 1.50 |
| *C.mollissima* | 95,748 | 71,436 | 24,312 | 18,795 | 4,085 | 3.80 |
| *C.nucifera* | 28,039 | 22,304 | 5,735 | 13,528 | 291 | 1.65 |
| *E.guineensis* | 25,636 | 23,149 | 2,487 | 13,942 | 132 | 1.66 |
| *J.curcas* | 21,529 | 20,307 | 1,222 | 14,091 | 177 | 1.44 |
| *J.regia* | 36,204 | 32,021 | 4,183 | 17,169 | 457 | 1.87 |
| *O.europaea* | 21,190 | 18,374 | 2,816 | 10,128 | 332 | 1.81 |
| *P.trichocarpa* | 31,415 | 29,525 | 1,890 | 13,852 | 257 | 2.13 |
| *R.communis* | 20,055 | 18,568 | 1,487 | 13,845 | 99 | 1.34 |
| *S.indicum* | 23,490 | 21,818 | 1,672 | 12,956 | 184 | 1.68 |
| *S.lycopersicum* | 34,726 | 25,819 | 8,907 | 14,081 | 963 | 1.83 |
| *V.vinifera* | 25,382 | 23,327 | 2,055 | 13,957 | 336 | 1.67 |
| *J. sigillata* | 30,387 | 26,539 | 3,848 | 16,438 | 141 | 1.61 |
