# Supplementary material for: Chromosomal-level assembly of Juglans sigillata genome using Nanopore, BioNano, and Hi-C analysis
Source: Gigascience. 2020 Feb 26;9(2):giaa006. doi: 10.1093/gigascience/giaa006 (PMC7043058; doi:10.1093/gigascience/giaa006)
Supplement: giaa006_Response_to_Reviewer_Comments_Original_Submission [file giaa006_response_to_reviewer_comments_original_submission.pdf]

Author's Response To Reviewer Comments

Close

Reviewer #1: The article describes the sequencing, assembly and annotation of the iron walnut tree (Juglans sigillata) as well as gene family-based analyzes.
1) My first point concerns the availability of the assembly and the annotation. There are available through the GigaScience repository, but they should be made available through the NCBI database, to be widely used and accessed.
Response: Thank you for your suggestion. Genome-related data has been submitted to the NCBI in the Short Read Archive under NCBI BioProject ID PRJNA509030 and will be made public after publication.

2) In addition, bioinformatics pipelines are poorly described in several sections, such as sections related to genome assembly and gene prediction.
Response: Thank you very much for your advice. We have added a more detailed description of bioinformatics pipelines.

3) Moreover, it should be interesting for the community to show how this new assembly of a Fagales genome compares with existing genome assemblies: Fagus, Quercus, Betula, Castanea… Contiguity and quality of genome assemblies is still heterogeneous and the methods used here allow generating a high quality assembly.
Response: Thank you! we compared the assembly differences between J. sigillata and related species, Seeing table 1.

4) Finally, the whole-genome duplication section is not rigorous and should be modified or removed.
Response: Thank you. Considering the Reviewer's suggestion, the whole-genome duplication section was removed after we carefully considered.

5) In addition, few points need to be addressed.
Genome Survey:
- Line 91: The formula seems incomplete and poorly formatted.
- Line 95: Authors should describe how the heterozygosity rate was estimated and how it compares to others Fagales.
Response:
The formula has been described in detail in lines 94-96.
The estimate of heterozygosity is described in detail in lines 99-100. And comparison of heterozygosity with other Fagales is shown in table 1.

6) Genome assembly:
- The parameters of Canu and wtdbg are not mentioned.
Scaffolding with Bionano optical mapping:
- Line 122: It is weird that the reported scaffold N50 is exactly the same than the one obtained for the optical map assembly.
- Line 122: Please specify the tools and parameters used to perform the hybrid scaffolding.
- Line 127: The gap-filling process must be described more precisely and the proportion of N's in each assembly must be reported.
Response: The parameters of Canu and wtdbg have been added in "Genome assembly" (line104 to line107).
It's a coincidence. Scaffold N50 of the optical map is 9.941 Mb, and scaffold N50 of the assembly genome is 9.942 Mb.
The tools and parameters used to perform the hybrid scaffolding have been added in "Scaffolding with BioNano optical mapping" (line130 to line132).
The gap-filling process has be described precisely and the proportion of N's has be reported in line 132-147.

7) Genome quality evaluation:
- Since the genome is heterozygous, the assembly must therefore contain allelic duplications. This

seems to be the case because the percentage of duplicated genes using BUSCO is high, around 10%. I think the authors should detect these allelic duplications and estimate the proportion of the assembly that is concerned.
Response: We have verified the problem of allelic duplications, as shown in line154-159.

8) Genome annotation
- The authors identify the transposable elements using RepeatMasker and Repbase, but it has been shown* that these tools can mask resistance genes. Did the authors take care of these R-genes ?
* https://www.nature.com/articles/s41477-018-0264-0
- Line 168: Please specify how the protein sequences were mapped on the J. sigillata genome.
- Line 181: Please specify how genes containing a premature termination were detected.
- Line 215: Replace J. curcas by J. regia.
Response: We did not deliberately focus on the R gene, but we did not screen out repetitive sequences in the annotation process to ensure the integrity of the annotation results.
-We added the mapping method in line 191.
-The detection method is added in line 203-204.
-We've replaced J. curcas by J. regia.

9) Genes under positive selection and gene family expansion analysis
- Line 225: Please provide more information on the functional annotation of genes under positive selection.
- Line 228-229: Please remove this sentence or provide additional information on the functional annotation of these gene families.
Response: The detailed description of the positive selection genes is shown in Additional file1 and lines 249-266.
-The additional information on the functional annotation of these gene families is provided in Additional file2, lines 269-277 and Supplementary Figure S5.

10) Whole-genome duplication
- The identification of WGD events cannot be done solely on the basis of 4DTv and Ks distributions. Authors must change the identification method or remove this section. The following manuscript* cites useful resources and contains tools that can be used to detect WGD events.
*https://academic.oup.com/bioinformatics/advance-article/doi/10.1093/bioinformatics/bty915/5162749
- Line 234: Typo V. vinifera.
Response:
Thank you! According to your suggestion, we have temporarily deleted the WGD section after much deliberation.
-Thank you! “V. vinifera” has been corrected.

11) Figures
- Figure 1, 2 and 5 have limited interest, I suggest moving these three figures in the Supplementary data. For a 'genome paper', I was expecting better figures comparing your genomes with other references and/or a global view of the J. sigillata genome.
- Figure 4: please add the color meaning of the pie charts.
Response: We have followed your wise advice, these figures were moved in the Supplementary data. and a global view of the J. sigillata genome is shown in figure 1.
-The color meaning of the pie charts is added.

12) Supplementary Material
- Please add more metrics to present the dataset and the assembly (Tables S1, S2, S6 and S7, such as N50, % of gaps, coverage…).
Response: According to your suggestions, we have added more detailed information in table S1, S2, S6, S7.

Reviewer #2: Wu et al. present a chromosome-level assembly of Juglans sigillata that has been assembled using second generation (Illumina) and third generation (Oxford nanopore) sequencing platforms, as well as optical mapping and Hi-C technologies. They further on carry out genome annotation using homology from related species as well as RNA-sequencing data.
After genome assembly and annotation they estimate genefamilies computationally using OrthoMCL, and estimate species tree and divergence times using single-copy orthologs. Finally, they identify the expanded orthogroups and genes under positive selection with kn/ks analysis, as well as the presence of

whole genome duplication events in the genome based on 4DTv sites.
1) While I find the paper technically correct, it completely lacks biological insights. The 529 expanded gene families/orthogroups are not studied at all, the functional enrichments are not assessed, and no link to biology or the evolution of the species is attempted.
Response: The additional information on the expanded gene families/orthogroups was provided in Additional file2, lines 269-277 and Supplementary Figure S5.

2) The same goes for the genes under positive selection, now it is mainly stated that 25 genes were identified with 20 having annotation in Swissprot. What were the functions? The genes should be listed, with information on their GO categories, and their feasibility should be discussed with literature review on putative orthologs in other species.
Response: The detailed description of the genes under positive selection was shown in Additional file1 and lines 249-266.

3) Whole genome duplication (WGD) was identified in J. regia at 55-66 Mya (Luo et al. BMC genomics 2015), how does the result presented here with J. sigillata relate to the earlier results?
Response: Thank you for your very wise advice. J. regia and J. sigillata diverged at about 49.49 Mya. The time that we calculated for J. sigillata to undergo WGD is about 60 Mya. Therefore, we hypothesize that the ancestors of J. regia and J. sigillata underwent this WGD and evolved in different directions to produce J. regia and J. sigillata species. However, only these evidences are still weak. Therefore, we delete WGD for the time being, so as to conduct further research.

4) Some parts of the text are very poorly written, for example p7, lines 99-103 describing the genome polishing after Nanopore assembly. Hence my recommendation is to carry out a major revision by including the biological interpretations of the results, as well as discussion comparing the results to the earlier Juglans paper, as well as possibly to other Fagales that have been sequenced, depending on the results. After revision the paper will need a complete new review.
Response: Based on your suggestions, we have carefully revised the paper, added biological interpretations, and compared the genome assembly of Fagales species.

Close
