# Supplementary material for: Chromosomal-level assembly of Juglans sigillata genome using Nanopore, BioNano, and Hi-C analysis
Source: Gigascience. 2020 Feb 26;9(2):giaa006. doi: 10.1093/gigascience/giaa006 (PMC7043058; doi:10.1093/gigascience/giaa006)
Supplement: giaa006_Response_to_Reviewer_Comments_Revision_1 [file giaa006_response_to_reviewer_comments_revision_1.pdf]

Author's Response To Reviewer Comments

Close

Reviewer reports:
Reviewer #1: The authors present an improved manuscript "Chromosome-level assembly of Juglans sigillata genome using Nanopore, BioNano and Hi-C analysis" and my major compulsory revisions were taken into account.

1) However, the manuscript still needs to be improved before acceptation. Moreover, the English language needs to be edited (such as sections on genes under positive selection).
Response: Thank you for your suggestion. We have corrected the text as suggested.

2) The authors verified that the depth of coverage of duplicated genes is almost identical to that of single-copy genes, but they have not described their alignment strategy: tool, parameters and filtering parameters. Moreover, I think a KAT analysis (https://github.com/TGAC/KAT) is more robust because it concern the whole genome and will show if the actual assembly contains duplicate kmers as well as the level of heterozygosity.
Response: Thanks. For the analysis, we refer to the durian (Durio zibethinus) genome article method, and the corresponding reference has been added to the article.

3) The authors did not describe how they computed the heterozygosity rates in Table 1, except for the studied genome. However I have doubts about the consistency of their method. Please use Genomescope (http://qb.cshl.edu/genomescope/) and kmer counting with the same parameters for all genome assemblies. The heterozygosity rate for Betula pendula is not reported, authors should indicate the reason.
Response: So sorry, it's our fault. The heterozygosity of the related species used for comparison was derived from the corresponding references, some of which did not provide sequencing data and therefore could not be recomputed using the same method. In order to be more rigorous, we added references uniformly (table 1 for details).

4.1) I think Table1 assemblies are not a faithful view of existing tree genome assemblies. The following review (Tuskan GA et al, Hardwood Tree Genomics: Unlocking Woody Plant Biology. Front Plant Sci. 2018 Dec 17) describes several resources that can be used here. For example, the Populus trichocarpa is a high-quality assembly, Quercus robur (Plomion C. et al, Oak genome reveals facets of long lifespan. Nat Plants. 2018 Jul) is of higher quality then Quercus lobata and high-quality Juglans genome assemblies are available in the NCBI database (1).
1- https://www.ncbi.nlm.nih.gov/assembly/organism/2249226/latest/
Response: Thank you for your advice. When sorting out the materials, we found that there were differences between the NCBI website and the corresponding reference reports. In order to present more information, we unified the data reported in the reference reports. Now, according to your suggestion, we have made optimization.

4.2) In addition, the metrics reported are different from those found on the NCBI website, for example, the contig N50 of the Betula pendula assembly is 49Kb at NCBI (2) but 240Kb in Table1, please check that the metrics are in accordance with the NCBI website.
2- https://www.ncbi.nlm.nih.gov/assembly/GCA_900184695.1
Response: Thanks! The metrics of the Betula pendula were modified according to the reference.

5) Table 1 must show the chromosome-level status of the assembly. Authors should add contig and scaffold number, proportion of gaps, metrics of the anchored assemblies as well as completion of the assemblies reported.
Response: As you suggested, we added some parameters to the table 1 that we could confirm.

6) The added content of the section "Genes under positive selection and gene family expansion analysis" is of poor quality, both in form and substance. There is a lack of references and the conclusions are too strong, especially the link between adaptation and the function of genes (l. 265 and 277).

Response: Thank you! According to your advice, we have made some modifications (line 240 to line 292).

Reviewer #2: In this second round of review the paper has improved and now attempts to address also biology. However, the results still need work.
1) For example, it is unclear whether the genes associated with chloroplast are truly significantly overrepresented and whether these six genes have any functional link with each other. The results overall should be put into context and the related literature should be discussed. Similarly, the paper reports an expansion in plant-pathogen interactions. This is a very broad category and it should be explored more to show which gene families are expanded and what do we know about similar expansions in other species. For example are these R genes?
Response: Thank you for your advice. The six genes associated with chloroplast activity or function are less studied. We tried to speculate their function by consulting published plant articles, but unfortunately, on the one hand, due to the lack of plant species closely related to J sigillata in similar plateau environment, on the other hand, researchers focused on different issues, so we could not find useful evidence or clues. However, we try to list the functions of these six genes found in the literature. To understand the relationships of the J. sigillata gene families with those of other plants, we performed the gene family expansion and contraction analysis. Among 529 expanded gene families, the largest proportion of genes was involved in the 'plant-pathogen interactions' (65, 12.29%), followed by the 'mRNA surveillance pathway' (44, 8.31%), 'Phospholipase D signaling pathway' (31, 5.86%), 'Fc gamma R-mediated phagocytosis' (31, 5.86%) and 'cAMP signaling pathway' (31, 5.86%). We have made a more detailed description in the revised article.

2.1) Additional remarks:
Line 100: "the heterozygosity of the genome was evaluated by Arabidopsis thaliana genome data fitting method.. " - a reference please.
Response: According to your suggestion, we have made corresponding modifications.

2.2) Line 110: "Nanopolish calibration uses the BWA default parameter to compare the quality-controlled Nanopore data to the assembled genome[13]. The second-generation data are then compared to the Nanopolish-corrected genome using the BWA default parameter, and the Pilon iteration is used to correct it two times" - still need to be written more clearly, please as a native English speaker to correct this.
Response: Thank you. Considering your suggestion, we have carefully revised the section (line110 to line115).

2.3) Line 138: "The genome was polished using BWA (BWA, RRID:SCR 010910) one time with default parameters. Reads from the Illumina DNA library (400bp) were then aligned against the genome assembly using the Pilon 1.22 " -
BWA is an aligner and Pilon a polisher. Please correct.
Response: Thank you. We have made modifications according to the suggestions (line139 to line142).

2.4) Line 142: "with gaps only 164" -> "with only 164 gaps"
Response: Thank you. We have corrected the mistake.

2.5) Line 246: "These genes might contribute to the adaption to harsh environments." - How does this come about? Some justification for this sentence, please.
Response: This is just a speculation based on the growing environment of J. sigillata, which may not be rigorous enough. Therefore, we temporarily remove this sentence.

2.6) Line 249: "Swissport" - fix typo.
Response: Thank you! The error has been corrected.

2.7) Line 249: "What's really amazing was that" - rephrase/remove. Non-scientific.
Response: According to the recommendation, the sentence has been removed.

2.8) Line 249-250: "What's really amazing was that six of these genes were involved in chloroplast function" - was this functional category ("chloroplast function") significantly enriched?
Response: Gene ontology (GO) analysis was performed with the condition P-value ≤ 0.05, so the functional category of pathway ("chloroplast function") is significantly enriched.

2.9) Lines 250-264: Please add references when discussing genes.

Response: Sorry, this is our negligence, now we have added the corresponding references in the revised article.

Close
