# Supplementary material for: Chromosomal-level assembly of Juglans sigillata genome using Nanopore, BioNano, and Hi-C analysis
Source: Gigascience. 2020 Feb 26;9(2):giaa006. doi: 10.1093/gigascience/giaa006 (PMC7043058; doi:10.1093/gigascience/giaa006)
Supplement: giaa006_Response_to_Reviewer_Comments_Revision_2 [file giaa006_response_to_reviewer_comments_revision_2.pdf]

Author's Response To Reviewer Comments

Close

Reviewer reports:
Reviewer #1: The authors present an improved manuscript "Chromosome-level assembly of Juglans sigillata genome using Nanopore, BioNano and Hi-C analysis". However, there are still some issues to be resolved.
1) Please describe how the analysis on allelic genes was performed; citing the durian article is not enough. I could not find information in the method section of this article.
Response: Thank you! The analysis on allelic genes has been described in detail in lines 156-157. Durian article had also been replaced.

2) Please add in Table 1 the completion of the selected assemblies: BUSCO scores.
Response: Thank you very much for your advice. We have added BUSCO scores in Table 1.

3) Heterozygosity rates (HR) were not calculated using the same method (Table 1), and I verified that raw sequencing data were available for at least C. illinoinensis, C. cathayensis, Q. lobata, Q. robur, and B. pendula. Please calculate the HR using the same method to support line 102-103 of the article.
Response: Considering the reviewer's suggestion, heterozygosity rates of C. illinoinensis, C. cathayensis, Q. lobata, and B. pendula have been calculated using the same method. The data volume of Q. robur is too little (only 6.1Gb) to calculate the heterozygosity. These results were shown in Additional file 1.

4) Some sentences must be verified: line 241, line 262-263, line 290-292.
Response: Considering the reviewer's suggestion, we supplement the references in the corresponding text, and delete the farfetched speculative sentences: "This may help J. sigillata adapt to the plateau environment."

5) Citation (1) from Table 1 is incorrect, please cite the GigaScience manuscript:
https://academic.oup.com/gigascience/article/8/5/giz036/5484800
Response: Thank you! The citation has been revised.

Reviewer #2: The authors have addressed my comments from the previous rounds. There are still minor modifications:
1.1) l 65: walnut breeding starts recently and -> has started
1.2) l 87: reading length -> read length
1.3) l 11: quality-controlled of Nanopore sequencing reads (remove 'of')
1.4) l 136: Blasr pipline -> pipeline
1.5) l 142-143: gaps length accounting -> gap length
1.6) l 156-157: The data coverage depth trend of duplicated genes - maybe it would be better to say that "the sequencing coverage of the duplicated genes"
1.7) l 175: de novo evolved annotation -> de novo repeat annotation
1.8) l 223: The results indicated that Gene family clustering (Remove 'The results indicated that')
Response: Thank you for your correction. We have carefully corrected these inappropriate wordings.

2) l 235-236: Correct the English
Response: Thank you for your suggestion, the English has been corrected.

3) l 251-252: Gene ontology (GO) analysis ($P \leq 0.05$) - define which method, additionally, did you use false discovery rate correction?
Response: Thanks! The method was added in the line 253, and we corrected it with Benjamini–Hochberg method.

4) l 281-282: Further, enrichment pipeline software was used to test the statistical enrichment of expanded gene families in KEGG - What is "enrichment pipeline software" - please define.
Response: Thanks, we have revised the content to make it easier to understand (line 283-287).

5) l 281: Did you only use KEGG? The enriched categories sound like GOs.
Response: Thanks! We have added the analysis results of GOs, as shown in Additional file 3.

6) l 282 Q-values - a reference to the method. Give also a short statement what the Q values are.
Response: Thanks! Q-values are the name given to the adjusted p-values found using an optimized FDR approach (line 286-287).

7) The highlighted text l240-292 should still be corrected for English and made more fluent.
Response: Thank you for your suggestion. We have carefully revised the English description.

Close
