# Supplementary material for: Chromosomal-level assembly of Juglans sigillata genome using Nanopore, BioNano, and Hi-C analysis
Source: Gigascience. 2020 Feb 26;9(2):giaa006. doi: 10.1093/gigascience/giaa006 (PMC7043058; doi:10.1093/gigascience/giaa006)
Supplement: giaa006_Reviewer_1_Report_Original_Submission — Jean-Marc Aury -- 1/24/2019 Reviewed [file giaa006_reviewer_1_report_original_submission.pdf]

Reviewer Report

Title: Chromosomal-level assembly of Juglans sigillata genome using Nanopore, BioNano and Hi-C analysis

Version: Original Submission **Date:** 1/24/2019

Reviewer name: Jean-Marc Aury

Reviewer Comments to Author:

The article describes the sequencing, assembly and annotation of the iron walnut tree (Juglans sigillata) as well as gene family-based analyzes. My first point concerns the availability of the assembly and the annotation. There are available through the GigaScience repository, but they should be made available through the NCBI database, to be widely used and accessed. In addition, bioinformatics pipelines are poorly described in several sections, such as sections related to genome assembly and gene prediction. Moreover, it should be interesting for the community to show how this new assembly of a Fagales genome compares with existing genome assemblies: Fagus, Quercus, Betula, Castanea… Contiguity and quality of genome assemblies is still heterogeneous and the methods used here allow generating a high quality assembly. Finally, the whole-genome duplication section is not rigorous and should be modified or removed. In addition, few points need to be addressed.

Genome Survey:

- Line 91: The formula seems incomplete and poorly formatted.
- Line 95: Authors should describe how the heterozygosity rate was estimated and how it compares to others Fagales.

Genome assembly:

- The parameters of Canu and wtdbg are not mentioned.

Scaffolding with Bionano optical mapping:

- Line 122: It is weird that the reported scaffold N50 is exactly the same than the one obtained for the optical map assembly.
- Line 122: Please specify the tools and parameters used to perform the hybrid scaffolding.
- Line 127: The gap-filling process must be described more precisely and the proportion of N's in each assembly must be reported.

Genome quality evaluation:

- Since the genome is heterozygous, the assembly must therefore contain allelic duplications. This seems to be the case because the percentage of duplicated genes using BUSCO is high, around 10%. I think the authors should detect these allelic duplications and estimate the proportion of the assembly that is concerned.

Genome annotation

- The authors identify the transposable elements using RepeatMasker and Repbase, but it has been shown* that these tools can mask resistance genes. Did the authors take care of these R-genes ?

* https://www.nature.com/articles/s41477-018-0264-0

- Line 168: Please specify how the protein sequences were mapped on the J. sigillata genome.

- Line 181: Please specify how genes containing a premature termination were detected.
- Line 215: Replace J. curcas by J. regia.

Genes under positive selection and gene family expansion analysis

- Line 225: Please provide more information on the functional annotation of genes under positive selection.
- Line 228-229: Please remove this sentence or provide additional information on the functional annotation of these gene families.

Whole-genome duplication

- The identification of WGD events cannot be done solely on the basis of 4DTv and Ks distributions. Authors must change the identification method or remove this section. The following manuscript* cites useful resources and contains tools that can be used to detect WGD events.

*https://academic.oup.com/bioinformatics/advance-article/doi/10.1093/bioinformatics/bty915/5162749

- Line 234: Typo V. vinifera.

Figures

- Figure 1, 2 and 5 have limited interest, I suggest moving these three figures in the Supplementary data. For a 'genome paper', I was expecting better figures comparing your genomes with other references and/or a global view of the J. sigillata genome.
- Figure 4: please add the color meaning of the pie charts.

Supplementary Material

- Please add more metrics to present the dataset and the assembly (Tables S1, S2, S6 and S7, such as N50, % of gaps, coverage…).

Level of Interest

Please indicate how interesting you found the manuscript: Choose an item.

Quality of Written English

Please indicate the quality of language in the manuscript: Choose an item.

Declaration of Competing Interests

Please complete a declaration of competing interests, considering the following questions:

- Have you in the past five years received reimbursements, fees, funding, or salary from an organisation that may in any way gain or lose financially from the publication of this manuscript, either now or in the future?
- Do you hold any stocks or shares in an organisation that may in any way gain or lose financially from the publication of this manuscript, either now or in the future?

- Do you hold or are you currently applying for any patents relating to the content of the manuscript?
- Have you received reimbursements, fees, funding, or salary from an organization that holds or has applied for patents relating to the content of the manuscript?
- Do you have any other financial competing interests?
- Do you have any non-financial competing interests in relation to this paper?

If you can answer no to all of the above, write 'I declare that I have no competing interests' below. If your reply is yes to any, please give details below.

I declare that I have no competing interests; however I should mention that I am part of the MinIONÂ® Access Programme (MAP) and I received travel and accommodation expenses to speak at Oxford Nanopore Technology conferences.

I agree to the open peer review policy of the journal. I understand that my name will be included on my report to the authors and, if the manuscript is accepted for publication, my named report including any attachments I upload will be posted on the website along with the authors' responses. I agree for my report to be made available under an Open Access Creative Commons CC-BY license (http://creativecommons.org/licenses/by/4.0/). I understand that any comments which I do not wish to be included in my named report can be included as confidential comments to the editors, which will not be published.

Choose an item.

To further support our reviewers, we have joined with Publons, where you can gain additional credit to further highlight your hard work (see: https://publons.com/journal/530/gigascience). On publication of this paper, your review will be automatically added to Publons, you can then choose whether or not to claim your Publons credit. I understand this statement.

Yes Choose an item.
