# Supplementary material for: Chromosomal-level assembly of Juglans sigillata genome using Nanopore, BioNano, and Hi-C analysis
Source: Gigascience. 2020 Feb 26;9(2):giaa006. doi: 10.1093/gigascience/giaa006 (PMC7043058; doi:10.1093/gigascience/giaa006)
Supplement: giaa006_Reviewer_1_Report_Revision_1 — Jean-Marc Aury -- 6/7/2019 Reviewed [file giaa006_reviewer_1_report_revision_1.pdf]

Reviewer Report

Title: Chromosomal-level assembly of Juglans sigillata genome using Nanopore, BioNano and Hi-C analysis

Version: Revision 1 **Date:** 6/7/2019

Reviewer name: Jean-Marc Aury

Reviewer Comments to Author:

The authors present an improved manuscript "Chromosome-level assembly of Juglans sigillata genome using Nanopore, BioNano and Hi-C analysis" and my major compulsory revisions were taken into account. However the manuscript still needs to be improved before acceptation. Moreover, the English language needs to be edited (such as sections on genes under positive selection).

- The authors verified that the depth of coverage of duplicated genes is almost identical to that of single-copy genes, but they have not described their alignment strategy: tool, parameters and filtering parameters. Moreover, I think a KAT analysis (https://github.com/TGAC/KAT) is more robust because it concern the whole genome and will show if the actual assembly contains duplicate kmers as well as the level of heterozygosity.
- The authors did not describe how they computed the heterozygosity rates in Table 1, except for the studied genome. However I have doubts about the consistency of their method. Please use Genomescope (http://qb.cshl.edu/genomescope/) and kmer counting with the same parameters for all genome assemblies. The heterozygosity rate for Betula pendula is not reported, authors should indicate the reason.
- I think Table1 assemblies are not a faithful view of existing tree genome assemblies. The following review (Tuskan GA et al, Hardwood Tree Genomics: Unlocking Woody Plant Biology. Front Plant Sci. 2018 Dec 17) describes several resources that can be used here. For example the Populus trichocarpa is a high-quality assembly, Quercus robur (Plomion C. et al, Oak genome reveals facets of long lifespan. Nat Plants. 2018 Jul) is of higher quality then Quercus lobata and high-quality Juglans genome assemblies are available in the NCBI database (1). In addition, the metrics reported are different from those found on the NCBI website, for example, the contig N50 of the Betula pendula assembly is 49Kb at NCBI (2) but 240Kb in Table1, please check that the metrics are in accordance with the NCBI website.

1- https://www.ncbi.nlm.nih.gov/assembly/organism/2249226/latest/

2- https://www.ncbi.nlm.nih.gov/assembly/GCA_900184695.1

- Table 1 must show the chromosome-level status of the assembly. Authors should add contig and scaffold number, proportion of gaps, metrics of the anchored assemblies as well as completion of the assemblies reported.
- The added content of the section "Genes under positive selection and gene family expansion analysis" is of poor quality, both in form and substance. There is a lack of references and the conclusions are too strong, especially the link between adaptation and the function of genes (l. 265 and 277).

Level of Interest

Please indicate how interesting you found the manuscript: Choose an item.

Quality of Written English

Please indicate the quality of language in the manuscript: Choose an item.

Declaration of Competing Interests

Please complete a declaration of competing interests, considering the following questions:

- Have you in the past five years received reimbursements, fees, funding, or salary from an organisation that may in any way gain or lose financially from the publication of this manuscript, either now or in the future?
- Do you hold any stocks or shares in an organisation that may in any way gain or lose financially from the publication of this manuscript, either now or in the future?
- Do you hold or are you currently applying for any patents relating to the content of the manuscript?
- Have you received reimbursements, fees, funding, or salary from an organization that holds or has applied for patents relating to the content of the manuscript?
- Do you have any other financial competing interests?
- Do you have any non-financial competing interests in relation to this paper?

If you can answer no to all of the above, write 'I declare that I have no competing interests' below. If your reply is yes to any, please give details below.

I declare no competing interests, however I received travel and accommodation expenses to speak at Oxford Nanopore Technology conferences.

I agree to the open peer review policy of the journal. I understand that my name will be included on my report to the authors and, if the manuscript is accepted for publication, my named report including any attachments I upload will be posted on the website along with the authors' responses. I agree for my report to be made available under an Open Access Creative Commons CC-BY license (http://creativecommons.org/licenses/by/4.0/). I understand that any comments which I do not wish to be included in my named report can be included as confidential comments to the editors, which will not be published.

Choose an item.

To further support our reviewers, we have joined with Publons, where you can gain additional credit to further highlight your hard work (see: https://publons.com/journal/530/gigascience). On publication of this paper, your review will be automatically added to Publons, you can then choose whether or not to claim your Publons credit. I understand this statement.

Yes Choose an item.
