# Supplementary material for: Chromosomal-level assembly of Juglans sigillata genome using Nanopore, BioNano, and Hi-C analysis
Source: Gigascience. 2020 Feb 26;9(2):giaa006. doi: 10.1093/gigascience/giaa006 (PMC7043058; doi:10.1093/gigascience/giaa006)
Supplement: giaa006_Reviewer_2_Report_Original_Submission — Jarkko Salojarvi, DSc (tech) -- 2/27/2019 Reviewed [file giaa006_reviewer_2_report_original_submission.pdf]

Reviewer Report

Title: Chromosomal-level assembly of Juglans sigillata genome using Nanopore, BioNano and Hi-C analysis

Version: Original Submission **Date:** 2/27/2019

Reviewer name: Jarkko Salojarvi, DSc (tech)

Reviewer Comments to Author:

Wu et al. present a chromosome-level assembly of Juglans sigillata that has been assembled using second generation (Illumina) and third generation (Oxford nanopore) sequencing platforms, as well as optical mapping and Hi-C technologies. They further on carry out genome annotation using homology from related species as well as RNA-sequencing data.
After genome assembly and annotation they estimate genefamilies computationally using OrthoMCL, and estimate species tree and divergence times using single-copy orthologs. Finally, they identify the expanded orthogroups and genes under positive selection with kn/ks analysis, as well as the presence of whole genome duplication events in the genome based on 4DTv sites.
While I find the paper technically correct, it completely lacks biological insights. The 529 expanded gene families/orthogroups are not studied at all, the functional enrichments are not assessed, and no link to biology or the evolution of the species is attempted. The same goes for the genes under positive selection, now it is mainly stated that 25 genes were identified with 20 having annotation in Swissprot. What were the functions? The genes should be listed, with information on their GO categories, and their feasibility should be discussed with literature review on putative orthologs in other species.
Whole genome duplication (WGD) was identified in J. regia at 55-66 Mya (Luo et al. BMC genomics 2015), how does the result presented here with J. sigillata relate to the earlier results?
Some parts of the text are very poorly written, for example p7, lines 99-103 describing the genome polishing after Nanopore assembly. Hence my recommendation is to carry out a major revision by including the biological interpretations of the results, as well as discussion comparing the results to the earlier Juglans paper, as well as possibly to other Fagales that have been sequenced, depending on the results. After revision the paper will need a complete new review.

Level of Interest

Please indicate how interesting you found the manuscript: Choose an item.

Quality of Written English

Please indicate the quality of language in the manuscript: Choose an item.

Declaration of Competing Interests

Please complete a declaration of competing interests, considering the following questions:

- Have you in the past five years received reimbursements, fees, funding, or salary from an organisation that may in any way gain or lose financially from the publication of this manuscript, either now or in the future?
- Do you hold any stocks or shares in an organisation that may in any way gain or lose financially from the publication of this manuscript, either now or in the future?
- Do you hold or are you currently applying for any patents relating to the content of the manuscript?
- Have you received reimbursements, fees, funding, or salary from an organization that holds or has applied for patents relating to the content of the manuscript?
- Do you have any other financial competing interests?
- Do you have any non-financial competing interests in relation to this paper?

If you can answer no to all of the above, write 'I declare that I have no competing interests' below. If your reply is yes to any, please give details below.

I declare that I have no competing interests.

I agree to the open peer review policy of the journal. I understand that my name will be included on my report to the authors and, if the manuscript is accepted for publication, my named report including any attachments I upload will be posted on the website along with the authors' responses. I agree for my report to be made available under an Open Access Creative Commons CC-BY license (http://creativecommons.org/licenses/by/4.0/). I understand that any comments which I do not wish to be included in my named report can be included as confidential comments to the editors, which will not be published.

Choose an item.

To further support our reviewers, we have joined with Publons, where you can gain additional credit to further highlight your hard work (see: https://publons.com/journal/530/gigascience). On publication of this paper, your review will be automatically added to Publons, you can then choose whether or not to claim your Publons credit. I understand this statement.

Yes Choose an item.
