# Supplementary material for: Chromosomal-level assembly of Juglans sigillata genome using Nanopore, BioNano, and Hi-C analysis
Source: Gigascience. 2020 Feb 26;9(2):giaa006. doi: 10.1093/gigascience/giaa006 (PMC7043058; doi:10.1093/gigascience/giaa006)
Supplement: giaa006_Reviewer_2_Report_Revision_1 — Jarkko Salojarvi, DSc (tech) -- 6/28/2019 Reviewed [file giaa006_reviewer_2_report_revision_1.pdf]

Reviewer Report

Title: Chromosomal-level assembly of Juglans sigillata genome using Nanopore, BioNano and Hi-C analysis

Version: Revision 1 **Date:** 6/28/2019

Reviewer name: Jarkko Salojarvi, DSc (tech)

Reviewer Comments to Author:

In this second round of review the paper has improved and now attempts to address also biology. However, the results still need work. For example, it is unclear whether the genes associated with chloroplast are truly significantly overrepresented and whether these six genes have any functional link with each other. The results overall should be put into context and the related literature should be discussed. Similarly, the paper reports an expansion in plant-pathogen interactions. This is a very broad category and it should be explored more to show which gene families are expanded and what do we know about similar expansions in other species. For example are these R genes?
Additional remarks:
Line 100: "the heterozygosity of the genome was evaluated by Arabidopsis thaliana genome data fitting method.. " - a reference please.
Line 110: "Nanopolish calibration uses the BWA default parameter
to compare the quality-controlled Nanopore data to the assembled genome[13]. The
second-generation data are then compared to the Nanopolish-corrected genome
using the BWA default parameter, and the Pilon iteration is used to correct it two
times" - still need to be written more clearly, please as a native English speaker to correct this
Line 138: "The genome was polished using BWA (BWA, RRID:SCR 010910) one time with default parameters. Reads from the Illumina DNA library (400bp) were then aligned against the genome assembly using the Pilon 1.22 " - BWA is an aligner and Pilon a polisher. Please correct.
Line 142: "with gaps only 164" -> "with only 164 gaps"
Line 246: "These genes might contribute to the adaption to harsh environments." - How does this come about? Some justification for this sentence, please.
Line 249: "Swissport" - fix typo.
Line 249: "What's really amazing was that" - rephrase/remove. Non-scientific.
Line 249-250: "What's really amazing was that six of these genes were involved in chloroplast function" - was this functional category ("chloroplast function") significantly enriched?
Lines 250-264: Please add references when discussing genes.

Level of Interest

Please indicate how interesting you found the manuscript: Choose an item.

Quality of Written English

Please indicate the quality of language in the manuscript: Choose an item.

Declaration of Competing Interests

Please complete a declaration of competing interests, considering the following questions:

- Have you in the past five years received reimbursements, fees, funding, or salary from an organisation that may in any way gain or lose financially from the publication of this manuscript, either now or in the future?
- Do you hold any stocks or shares in an organisation that may in any way gain or lose financially from the publication of this manuscript, either now or in the future?
- Do you hold or are you currently applying for any patents relating to the content of the manuscript?
- Have you received reimbursements, fees, funding, or salary from an organization that holds or has applied for patents relating to the content of the manuscript?
- Do you have any other financial competing interests?
- Do you have any non-financial competing interests in relation to this paper?

If you can answer no to all of the above, write 'I declare that I have no competing interests' below. If your reply is yes to any, please give details below.

I declare that I have no competing interests

I agree to the open peer review policy of the journal. I understand that my name will be included on my report to the authors and, if the manuscript is accepted for publication, my named report including any attachments I upload will be posted on the website along with the authors' responses. I agree for my report to be made available under an Open Access Creative Commons CC-BY license (http://creativecommons.org/licenses/by/4.0/). I understand that any comments which I do not wish to be included in my named report can be included as confidential comments to the editors, which will not be published.

Choose an item.

To further support our reviewers, we have joined with Publons, where you can gain additional credit to further highlight your hard work (see: https://publons.com/journal/530/gigascience). On publication of this paper, your review will be automatically added to Publons, you can then choose whether or not to claim your Publons credit. I understand this statement.

Yes Choose an item.
